# Supplementary figures and images for: Activation of goblet-cell stress sensor IRE1β is controlled by the mucin chaperone AGR2 (part 1 of 2)
Source: EMBO J. 2023 Dec 20;43(5):3. doi: 10.1038/s44318-023-00015-y (PMC10907643; doi:10.1038/s44318-023-00015-y)

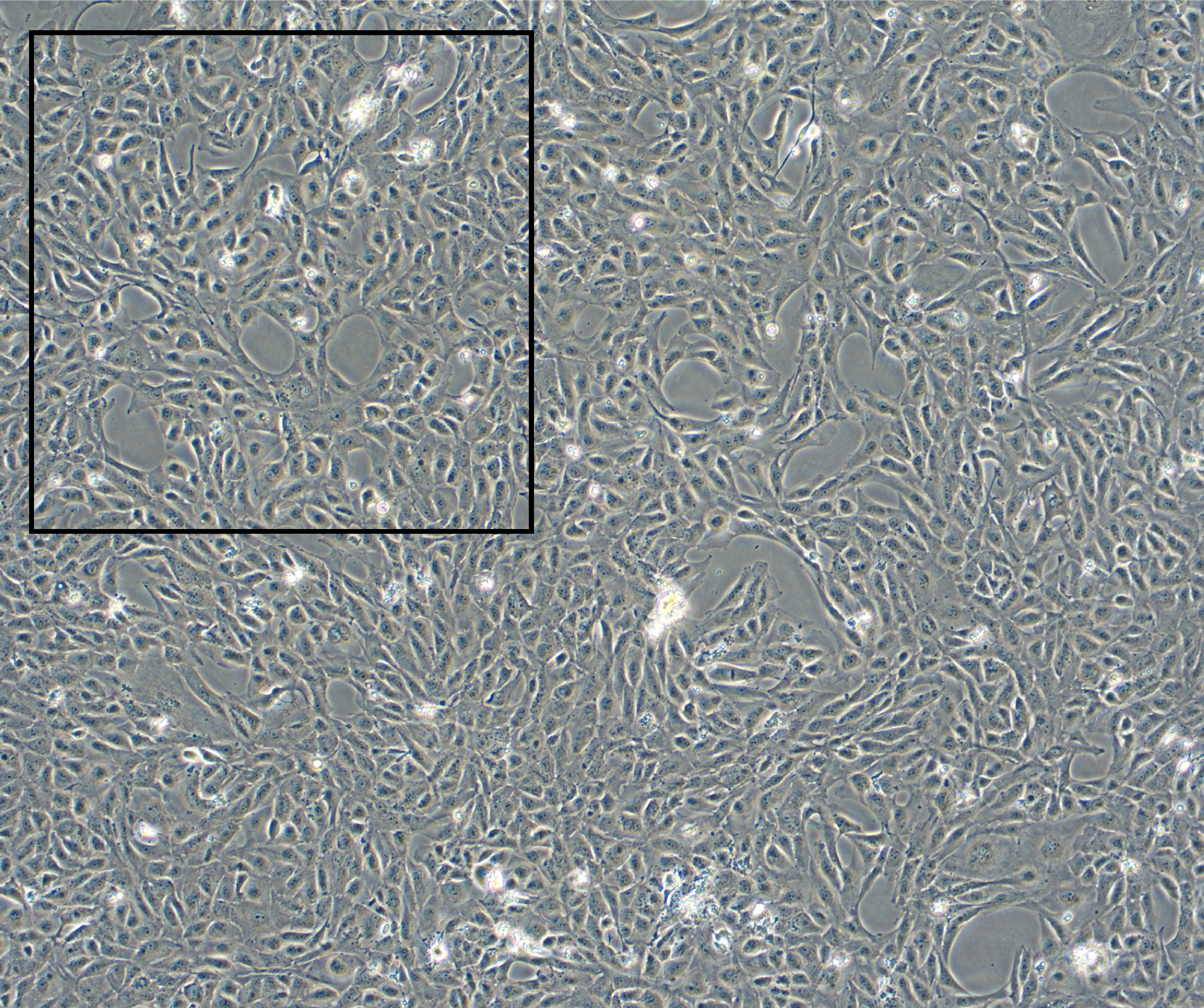

Supplement: Supplementary file 3 — Source Data Fig. 1 [file 44318_2023_15_MOESM3_ESM.zip › Figure 1/1C/Calu-1 - doxycycline and 4u8C.tif]

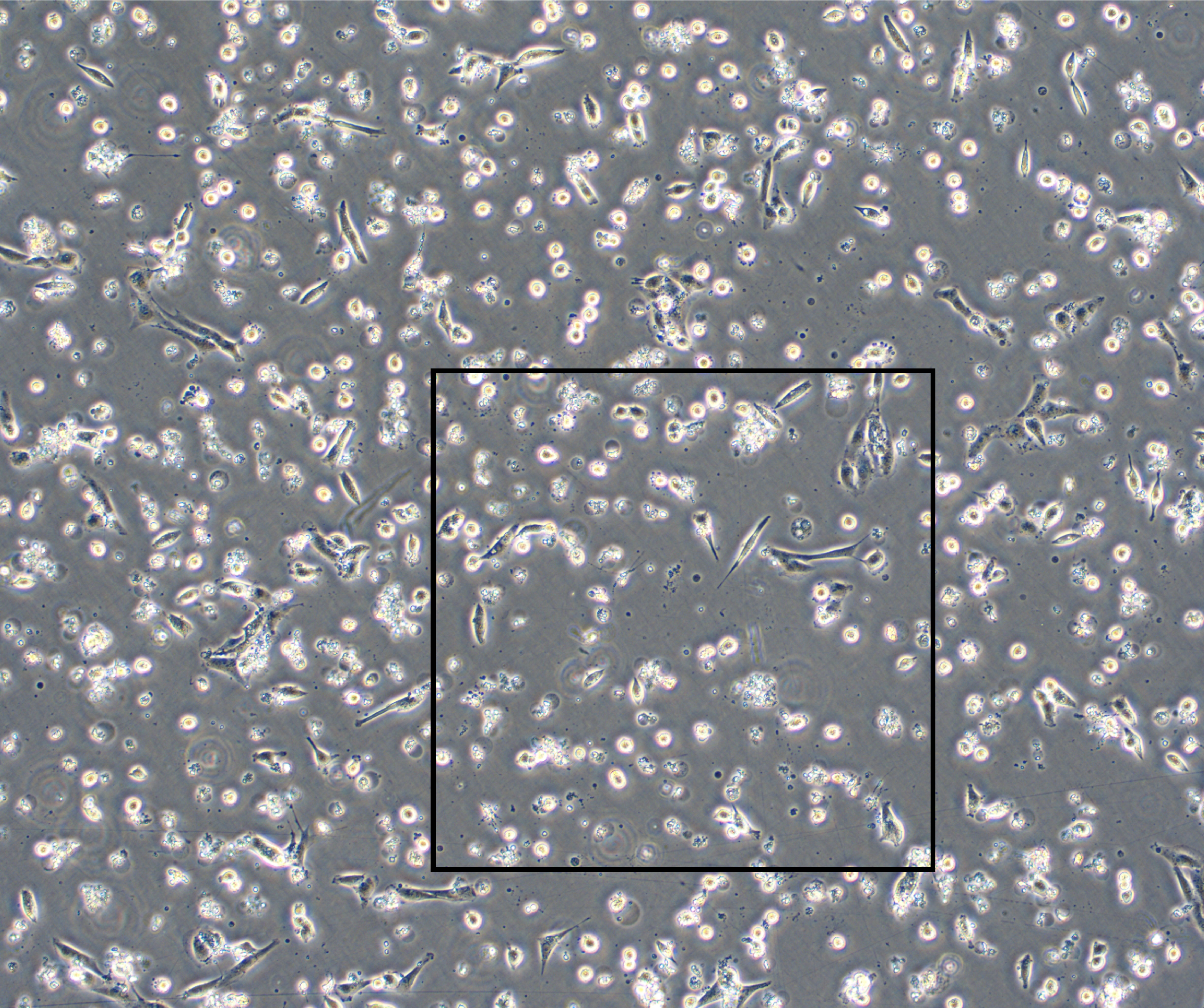

Supplement: Supplementary file 3 — Source Data Fig. 1 [file 44318_2023_15_MOESM3_ESM.zip › Figure 1/1C/Calu-1 - doxycycline.tif]

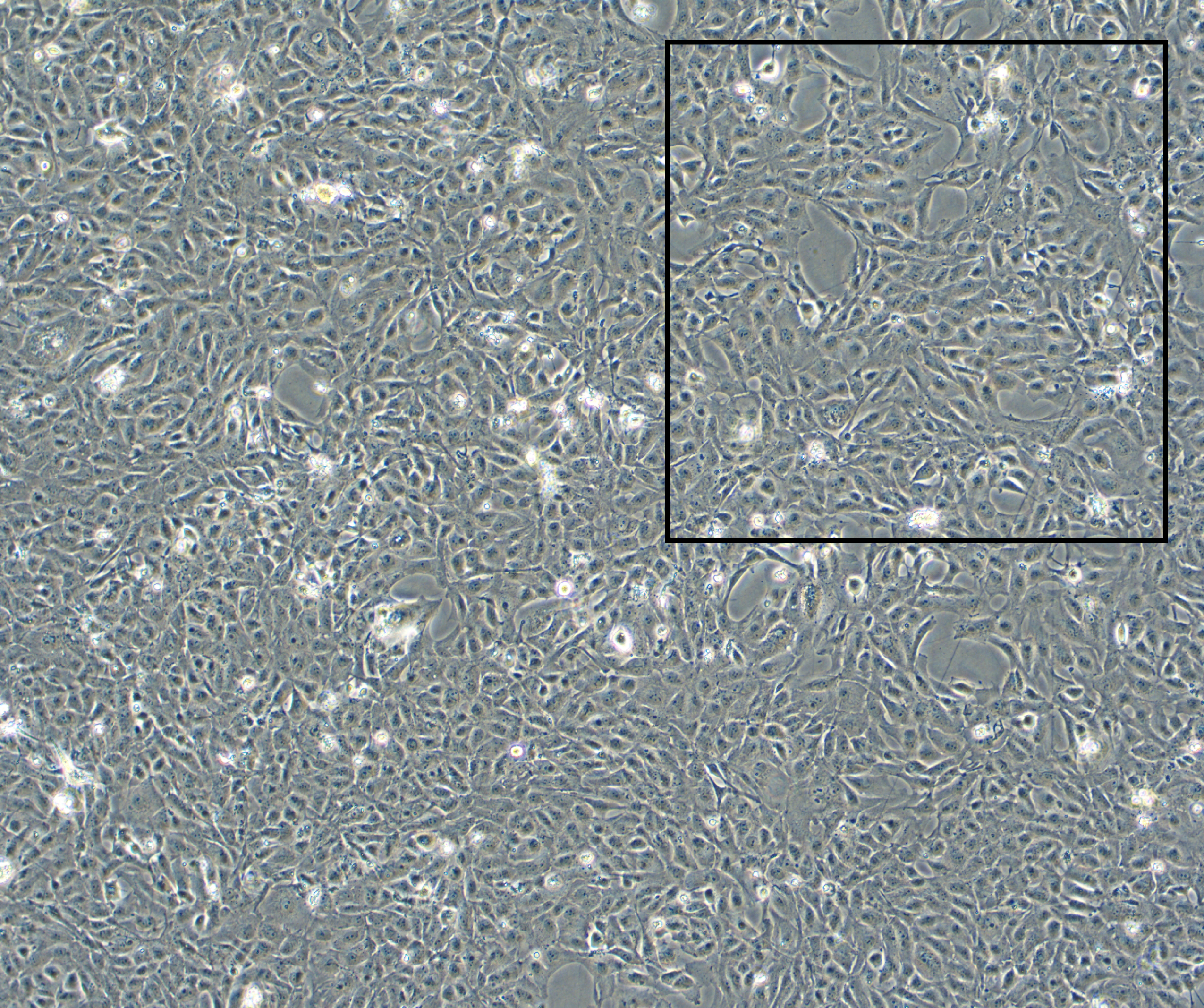

Supplement: Supplementary file 3 — Source Data Fig. 1 [file 44318_2023_15_MOESM3_ESM.zip › Figure 1/1C/Calu-1 - Untreated.tif]

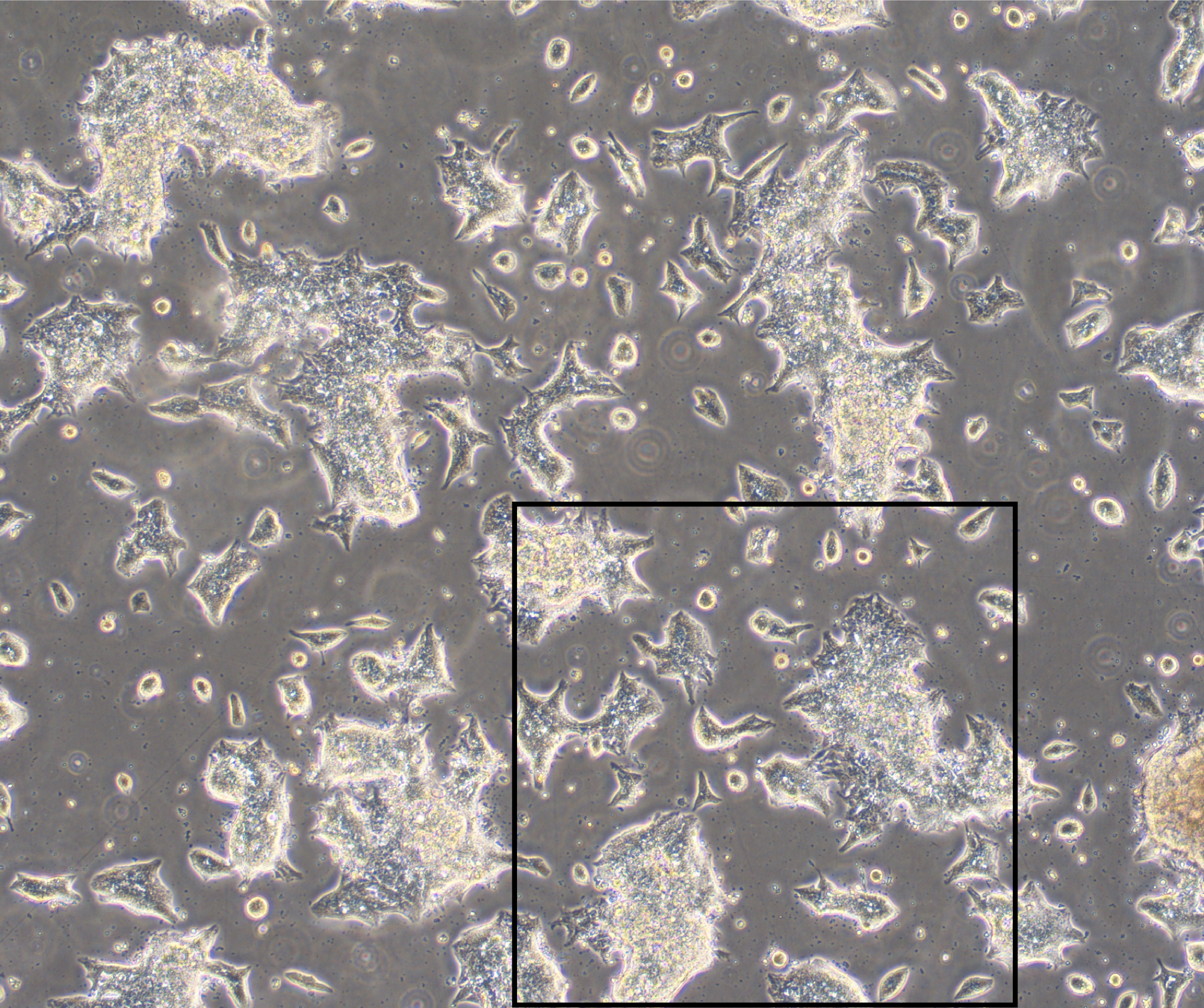

Supplement: Supplementary file 3 — Source Data Fig. 1 [file 44318_2023_15_MOESM3_ESM.zip › Figure 1/1C/LS174T - doxycycline and 4u8C.tif]

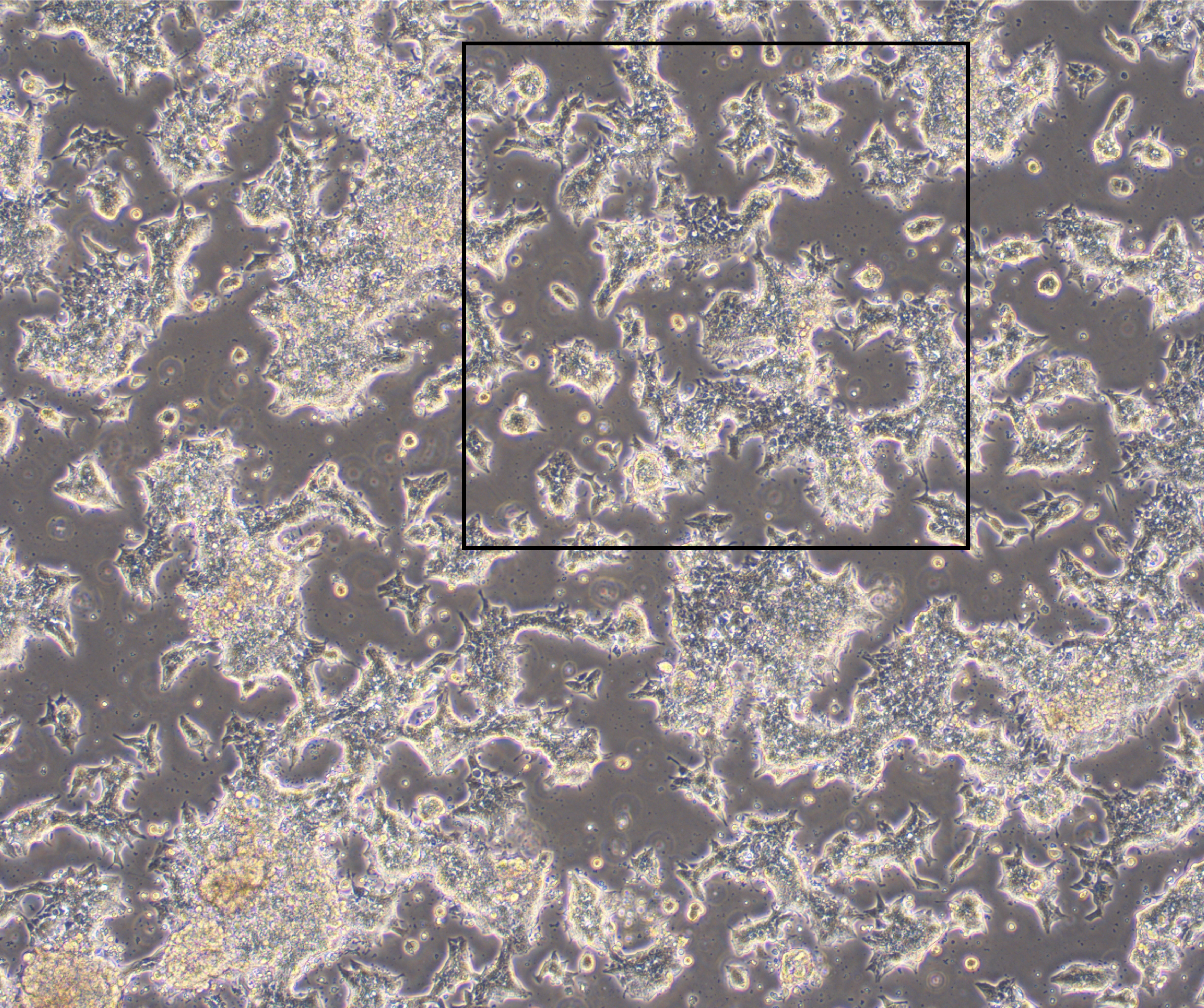

Supplement: Supplementary file 3 — Source Data Fig. 1 [file 44318_2023_15_MOESM3_ESM.zip › Figure 1/1C/LS174T - doxycycline.tif]

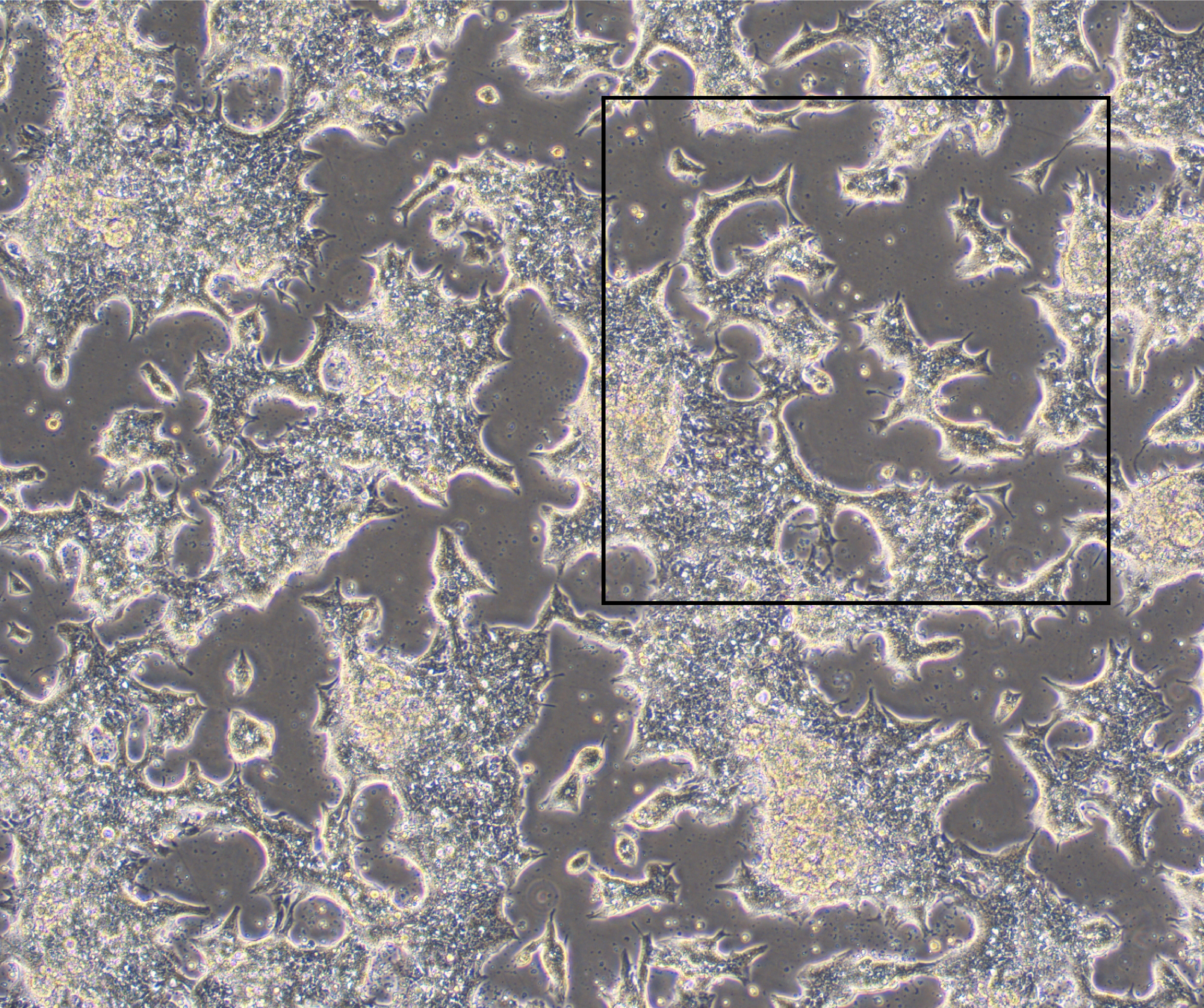

Supplement: Supplementary file 3 — Source Data Fig. 1 [file 44318_2023_15_MOESM3_ESM.zip › Figure 1/1C/LS174T - Untreated.tif]

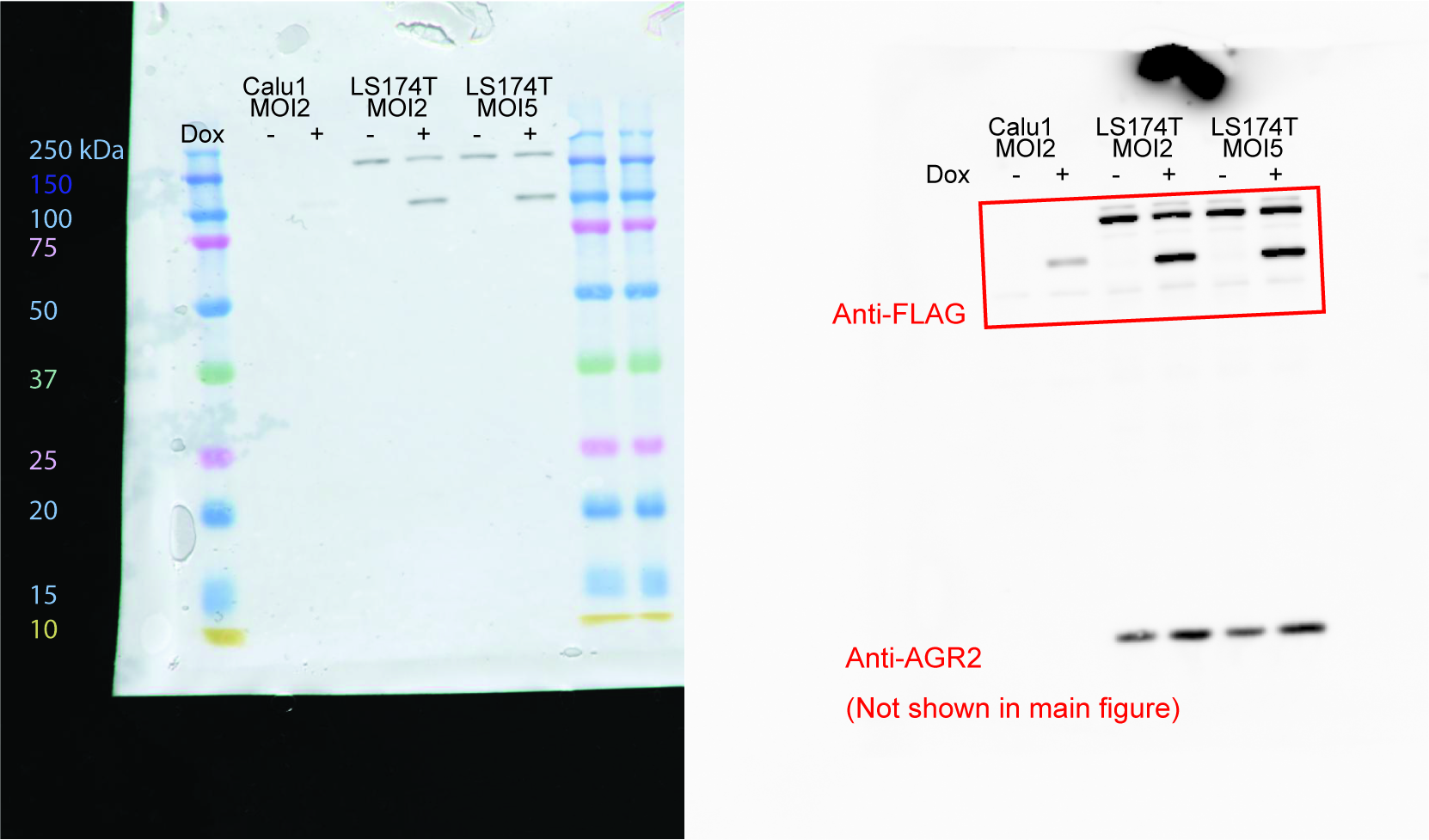

Supplement: Supplementary file 3 — Source Data Fig. 1 [file 44318_2023_15_MOESM3_ESM.zip › Figure 1/1D/western FLAG.tif]

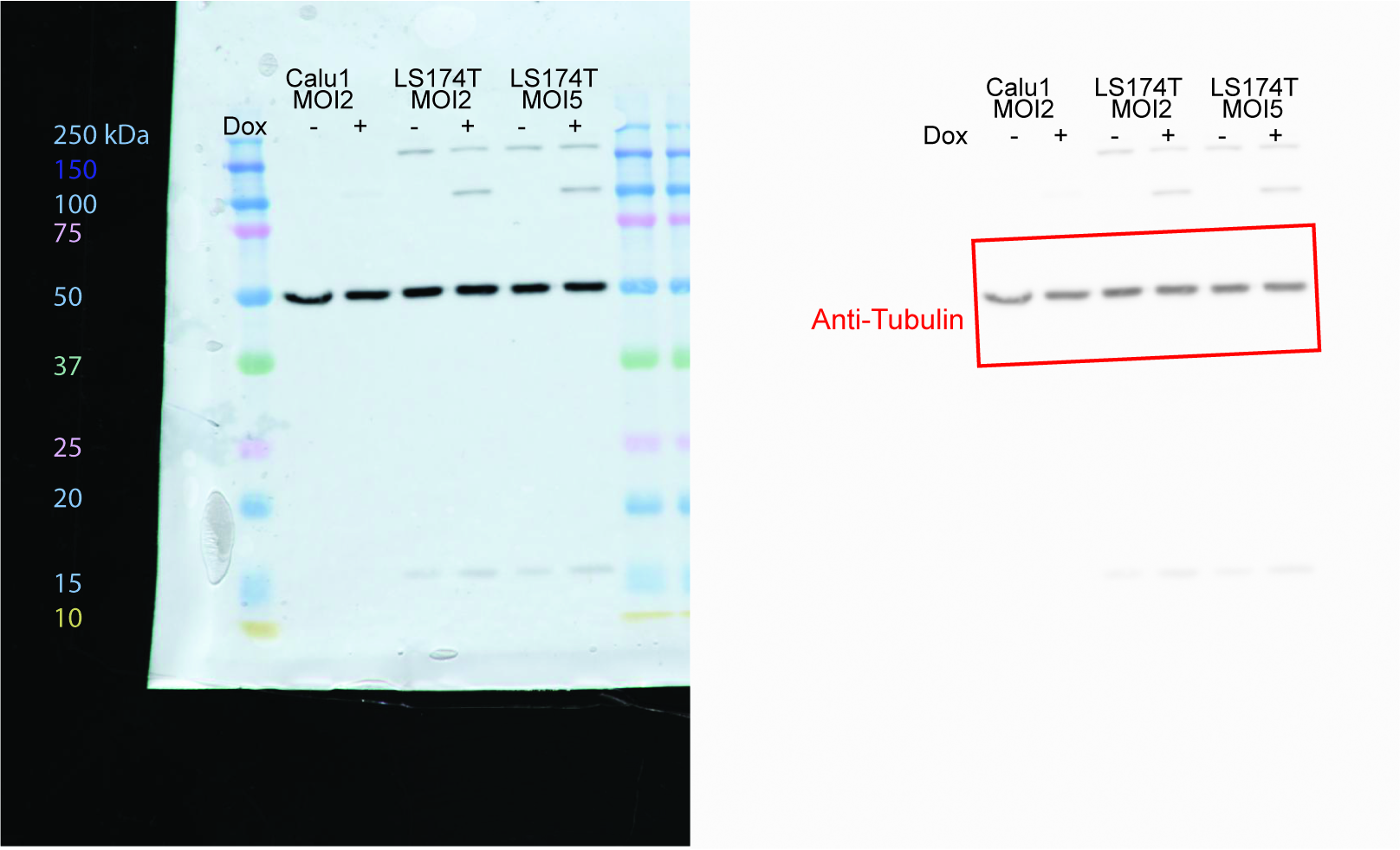

Supplement: Supplementary file 3 — Source Data Fig. 1 [file 44318_2023_15_MOESM3_ESM.zip › Figure 1/1D/western tubulin.tif]

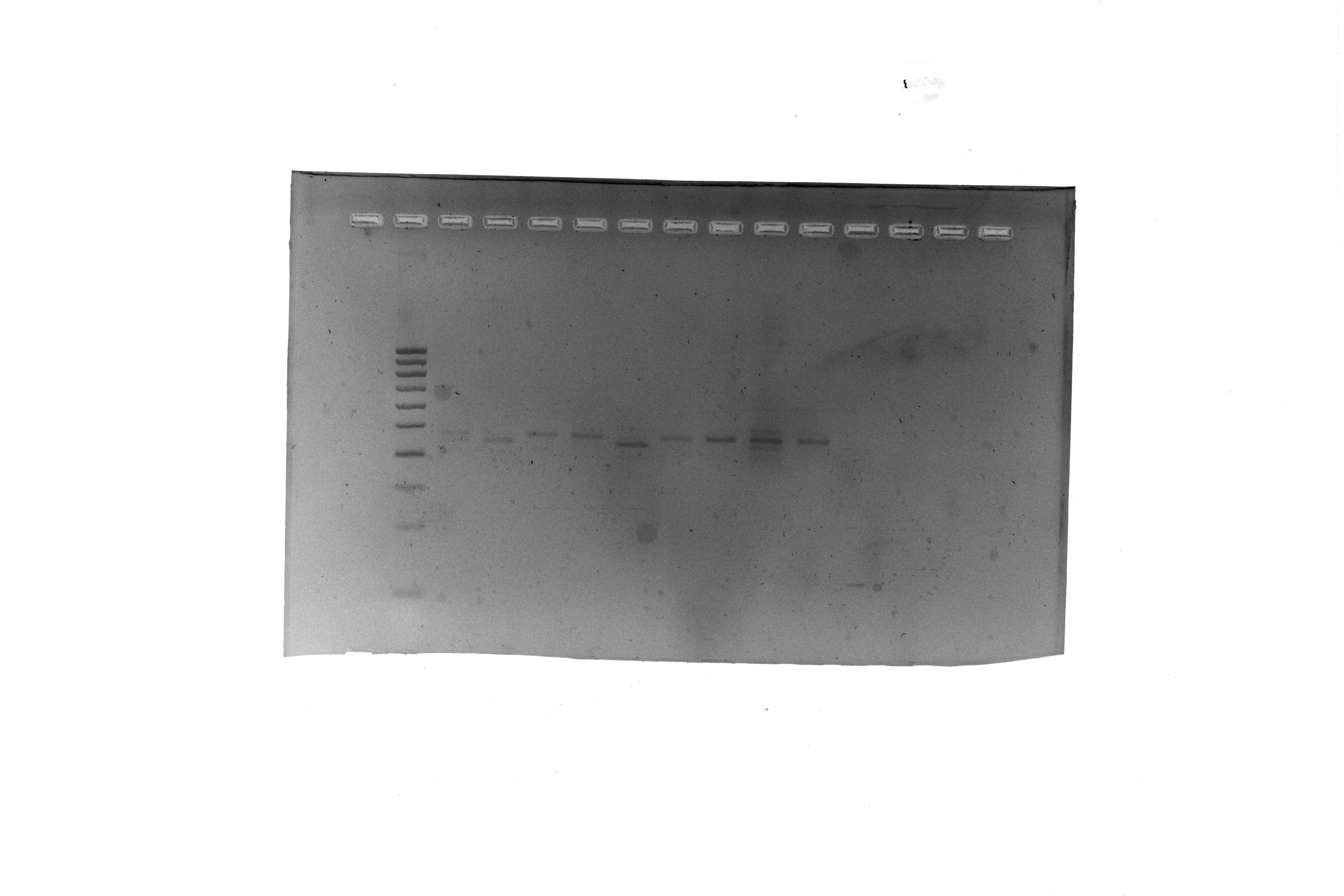

Supplement: Supplementary file 3 — Source Data Fig. 1 [file 44318_2023_15_MOESM3_ESM.zip › Figure 1/1E/Replicate/PCR gel XBP1S repl1.TIFF]

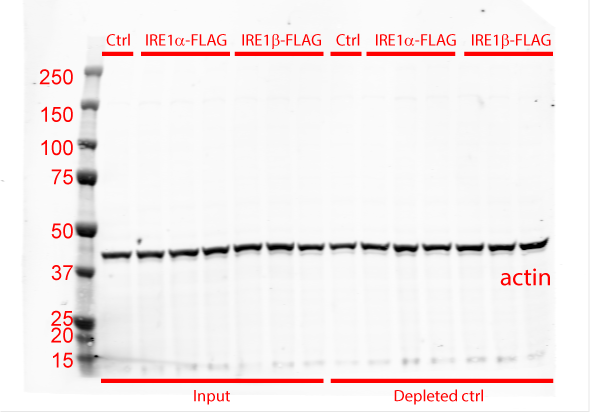

Supplement: Supplementary file 4 — Source Data Fig. 2 [file 44318_2023_15_MOESM4_ESM.zip › Figure 2/2A/western input actin.tif]

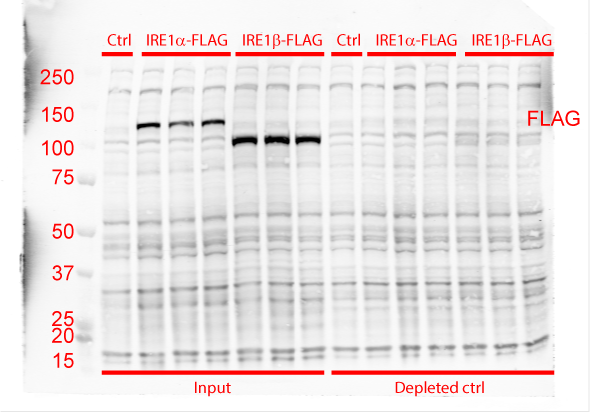

Supplement: Supplementary file 4 — Source Data Fig. 2 [file 44318_2023_15_MOESM4_ESM.zip › Figure 2/2A/western input FLAG.tif]

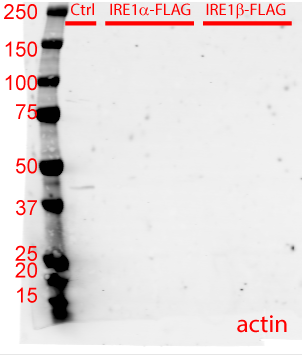

Supplement: Supplementary file 4 — Source Data Fig. 2 [file 44318_2023_15_MOESM4_ESM.zip › Figure 2/2A/western IP actin.tif]

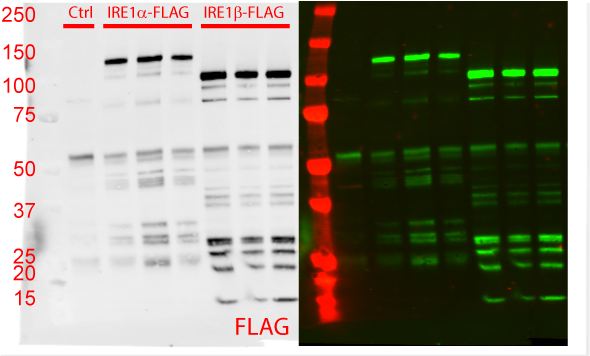

Supplement: Supplementary file 4 — Source Data Fig. 2 [file 44318_2023_15_MOESM4_ESM.zip › Figure 2/2A/western IP FLAG.tif]

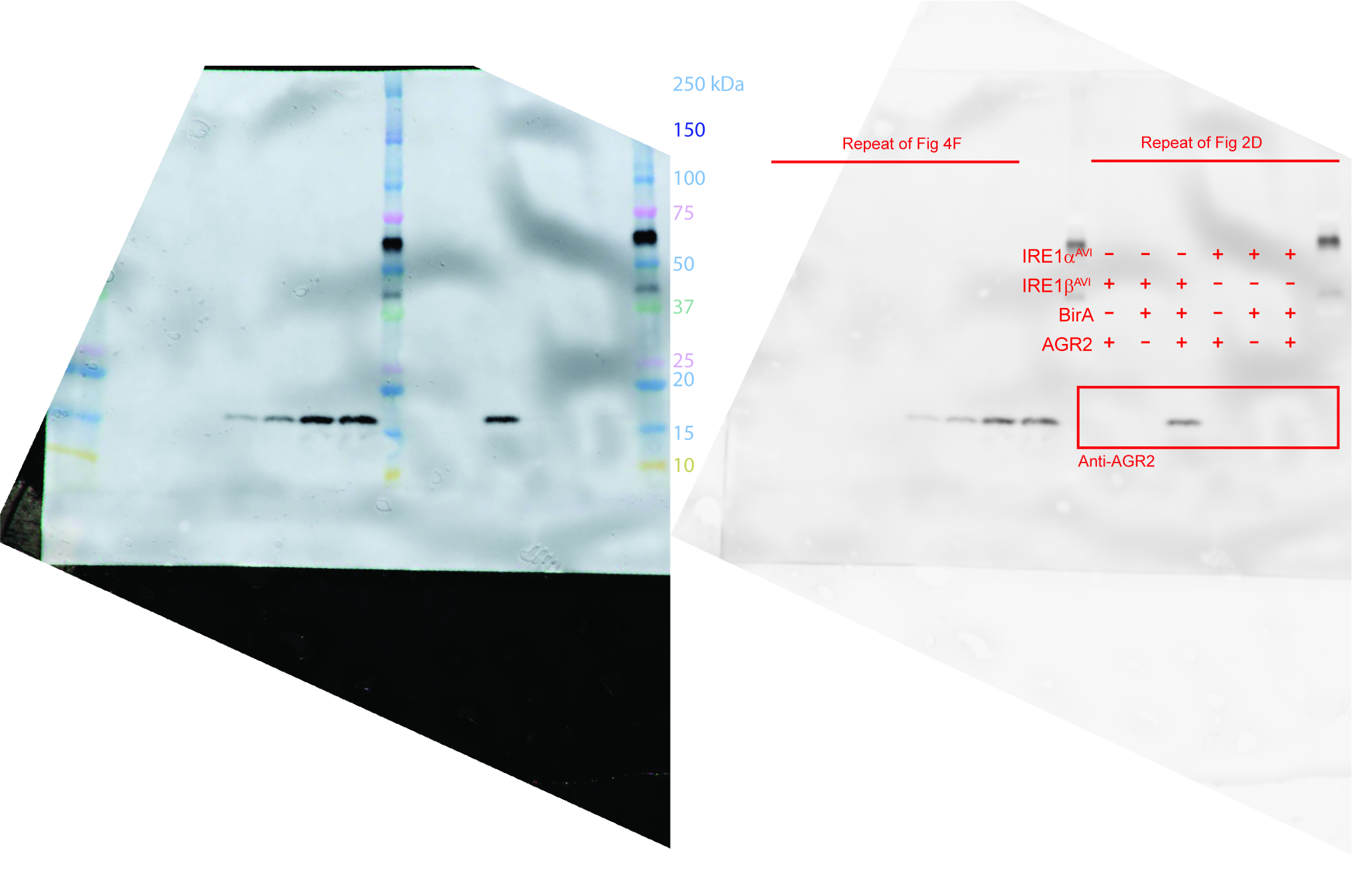

Supplement: Supplementary file 4 — Source Data Fig. 2 [file 44318_2023_15_MOESM4_ESM.zip › Figure 2/2D/Replicate/western AGR2 - IP samples.tif]

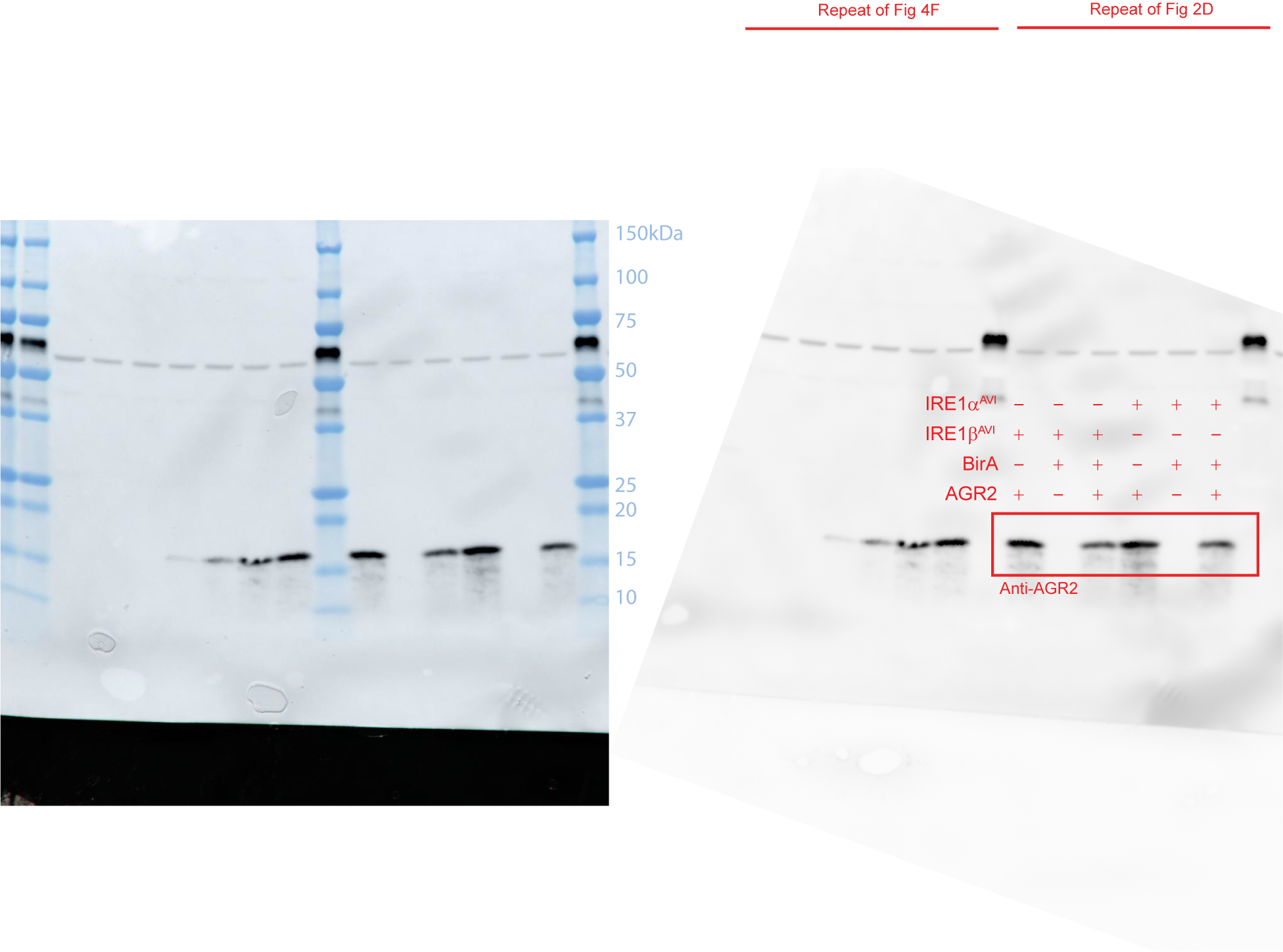

Supplement: Supplementary file 4 — Source Data Fig. 2 [file 44318_2023_15_MOESM4_ESM.zip › Figure 2/2D/Replicate/western AGR2 - input samples.tif]

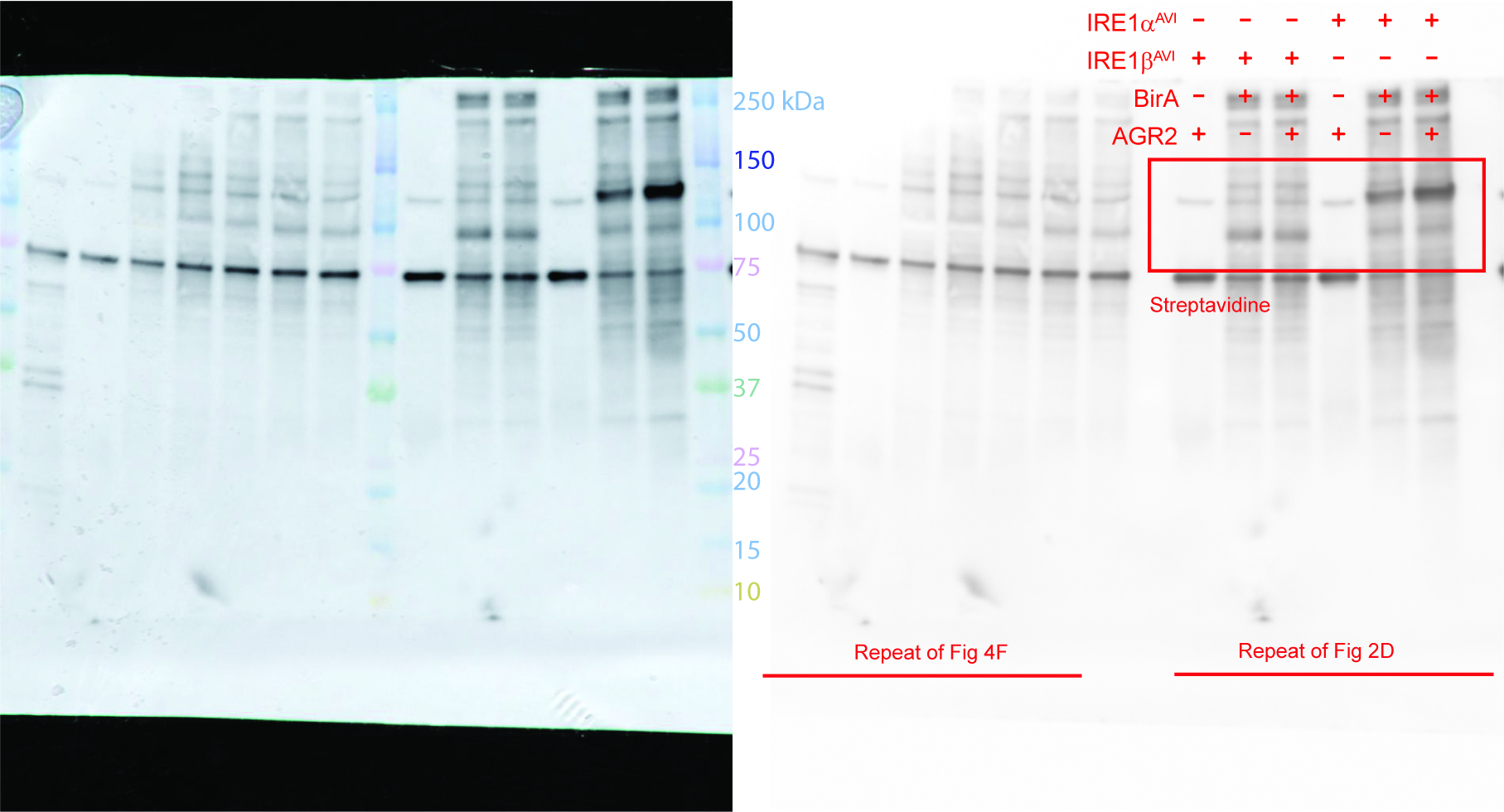

Supplement: Supplementary file 4 — Source Data Fig. 2 [file 44318_2023_15_MOESM4_ESM.zip › Figure 2/2D/Replicate/western streptavidine - IP samples.tif]

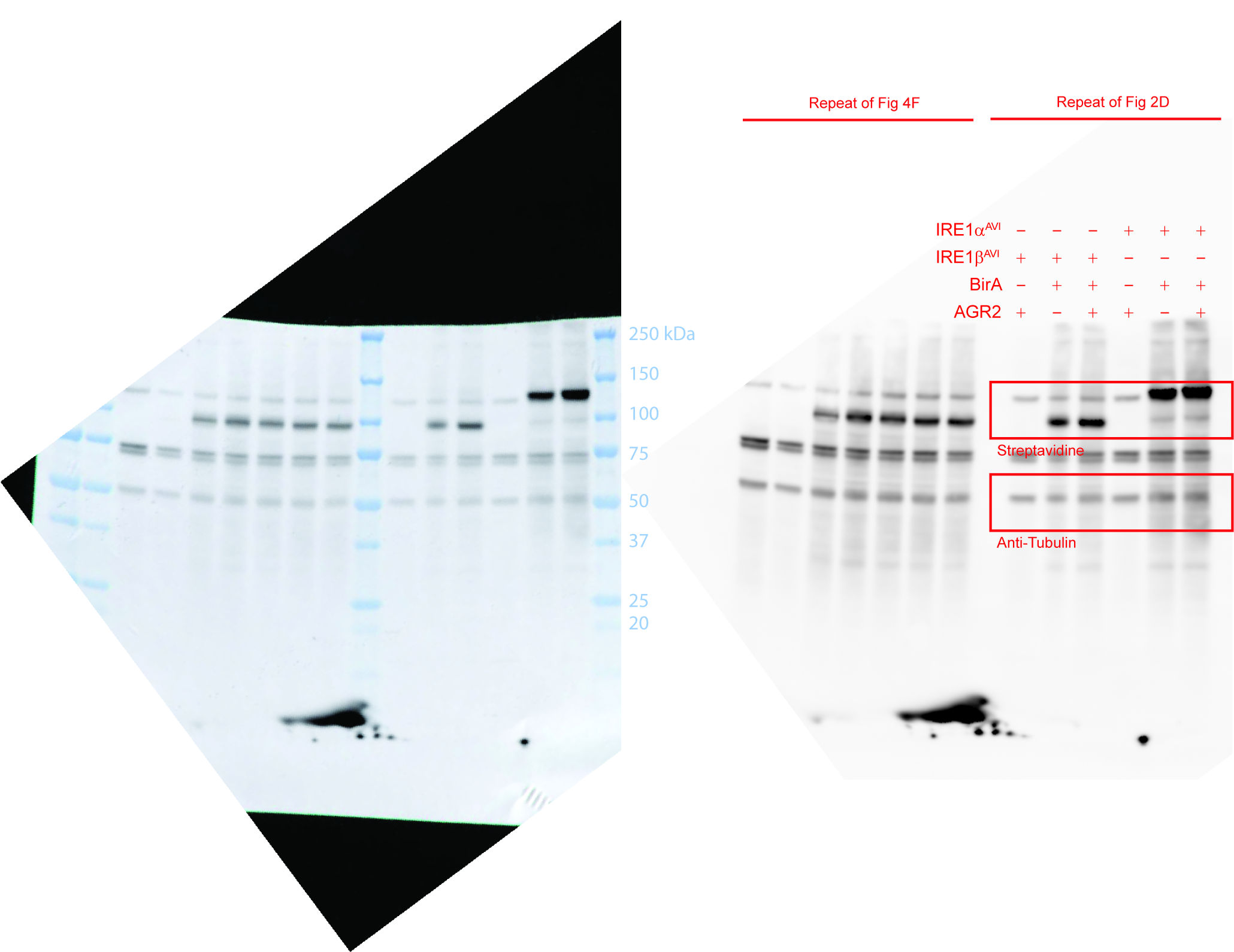

Supplement: Supplementary file 4 — Source Data Fig. 2 [file 44318_2023_15_MOESM4_ESM.zip › Figure 2/2D/Replicate/western streptavidine and tubulin - input samples.tif]

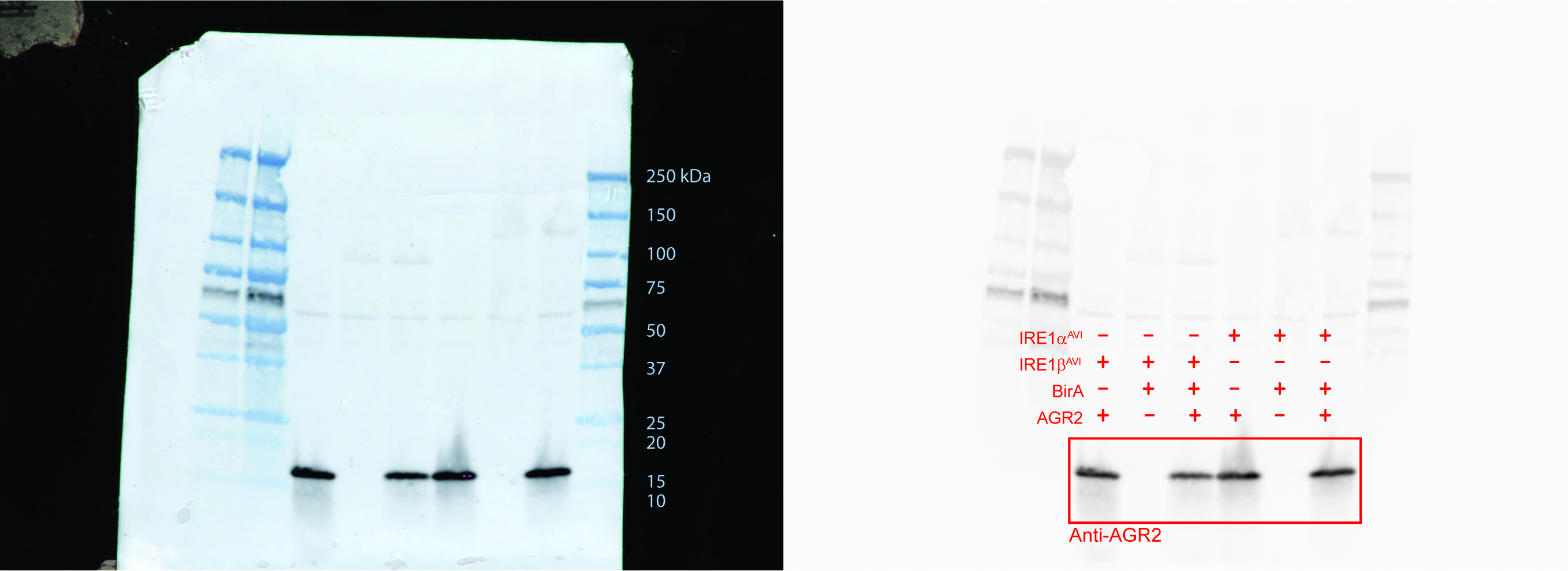

Supplement: Supplementary file 4 — Source Data Fig. 2 [file 44318_2023_15_MOESM4_ESM.zip › Figure 2/2D/western AGR2 - input samples.tif]

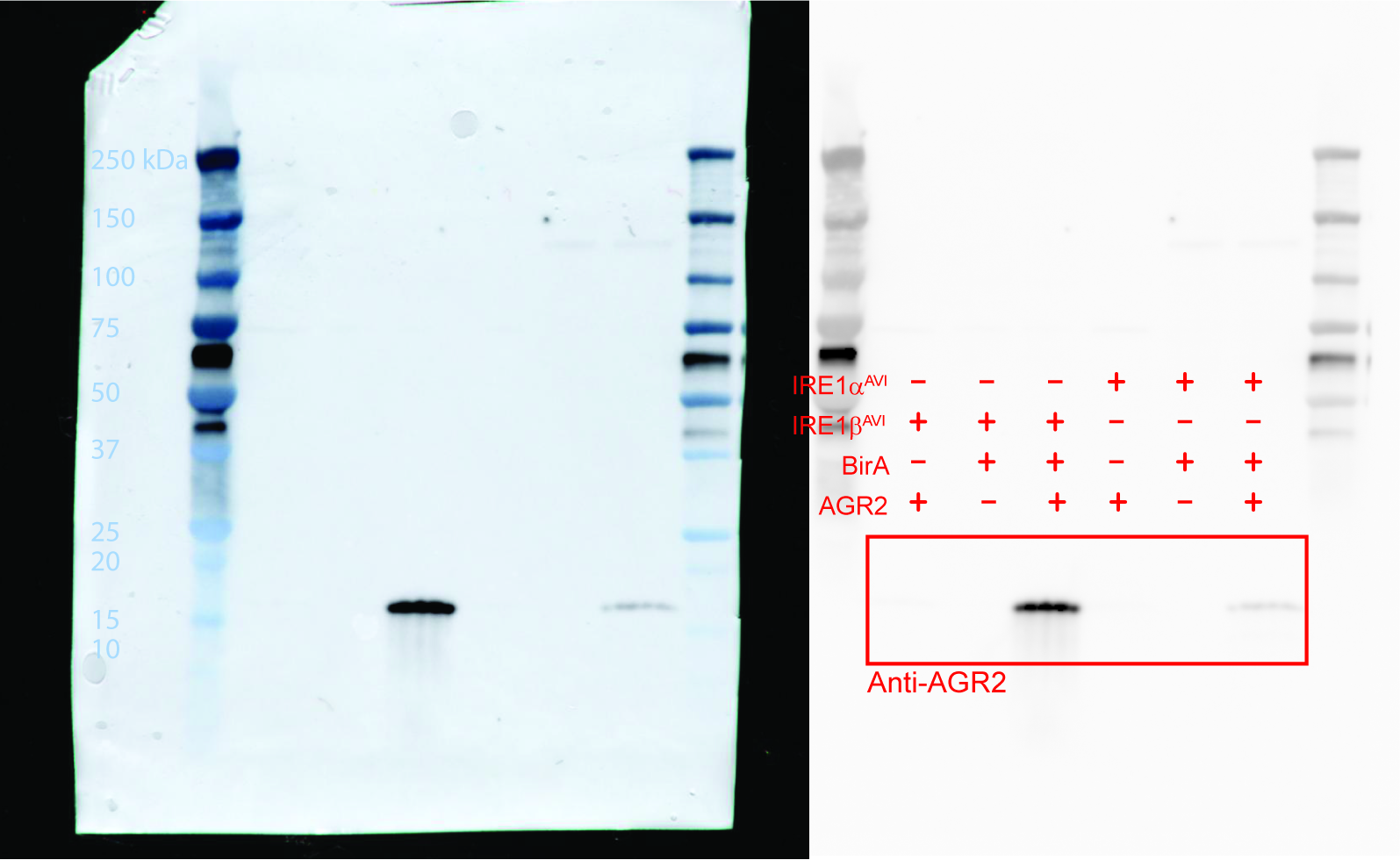

Supplement: Supplementary file 4 — Source Data Fig. 2 [file 44318_2023_15_MOESM4_ESM.zip › Figure 2/2D/western AGR2 - IP samples.tif]

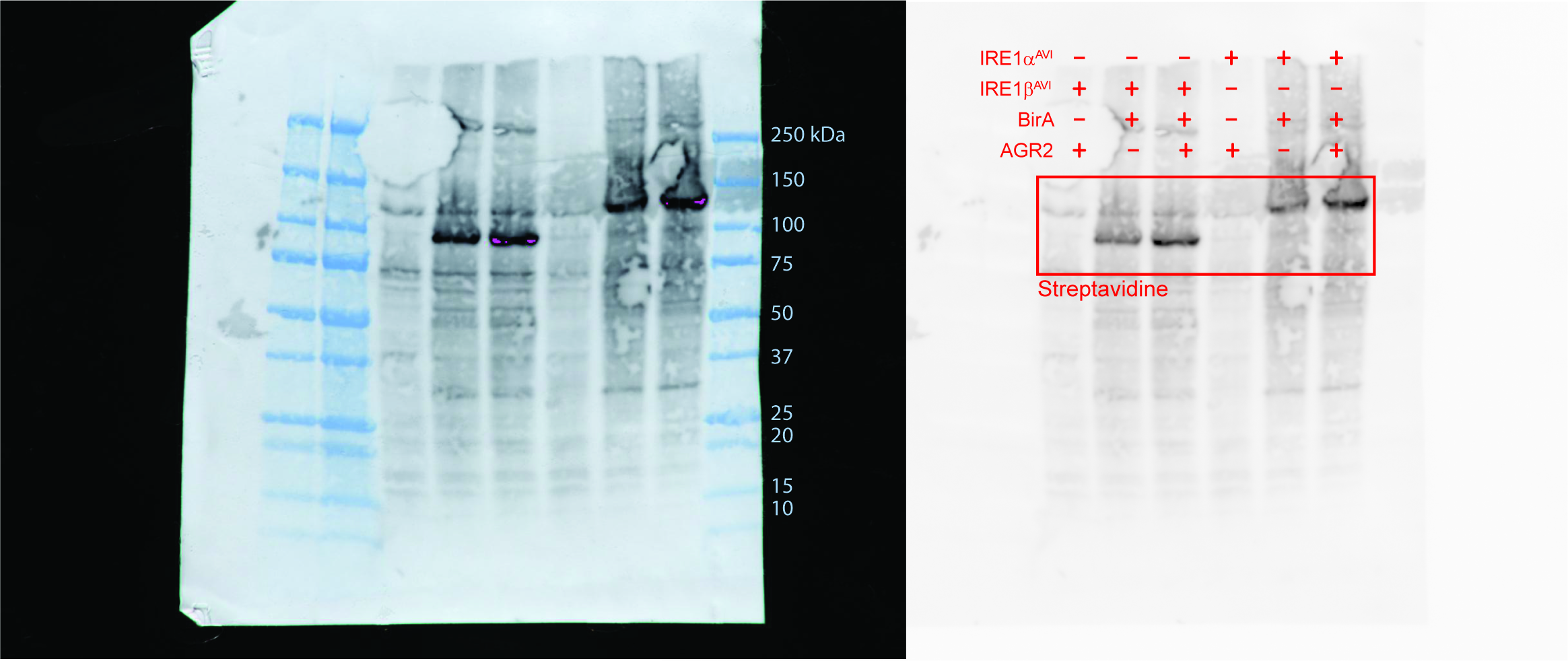

Supplement: Supplementary file 4 — Source Data Fig. 2 [file 44318_2023_15_MOESM4_ESM.zip › Figure 2/2D/western streptavidine - input samples.tif]

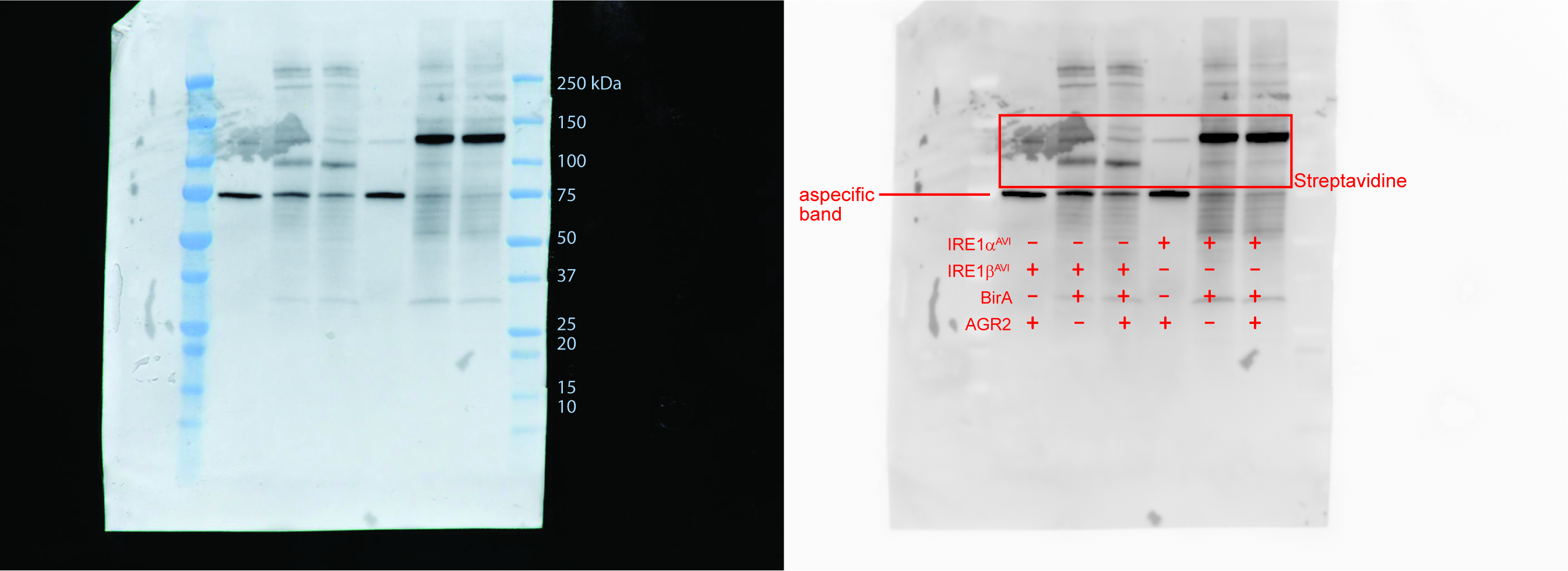

Supplement: Supplementary file 4 — Source Data Fig. 2 [file 44318_2023_15_MOESM4_ESM.zip › Figure 2/2D/western streptavidine - IP samples.tif]

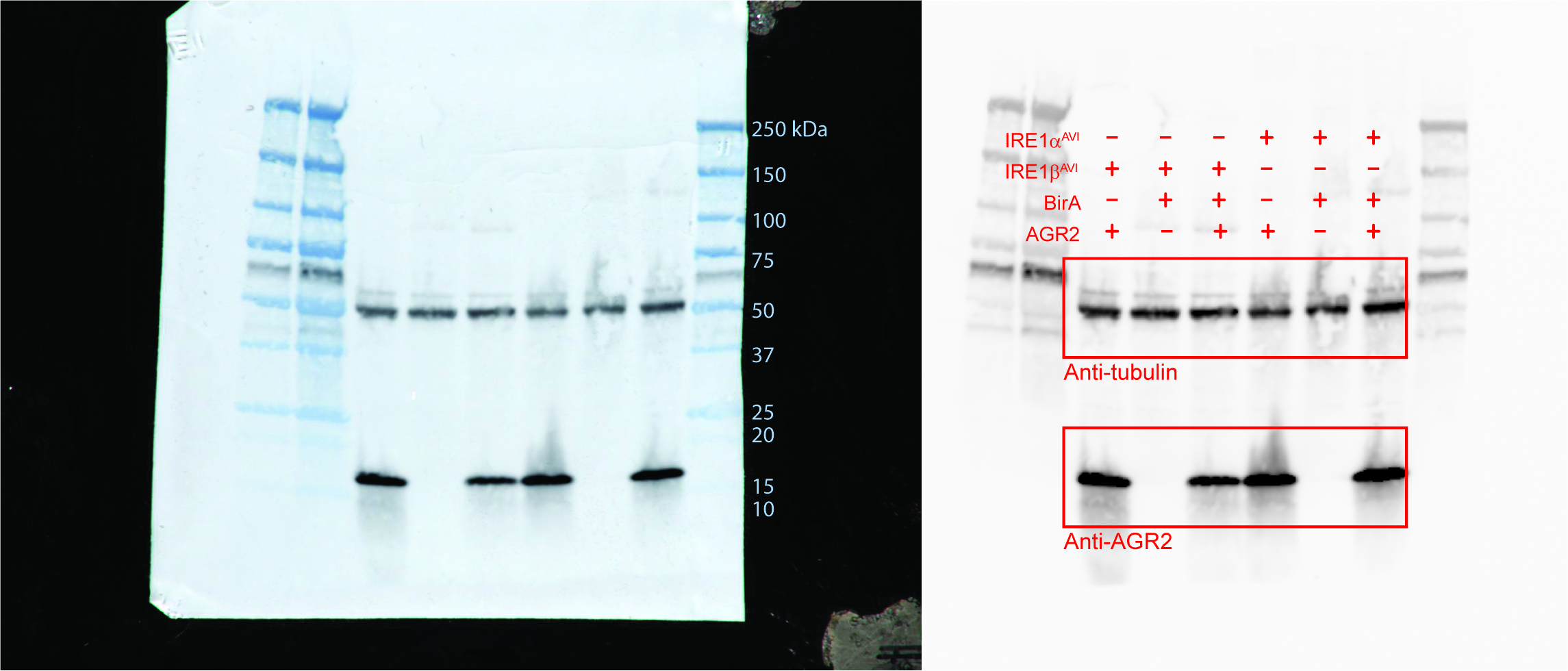

Supplement: Supplementary file 4 — Source Data Fig. 2 [file 44318_2023_15_MOESM4_ESM.zip › Figure 2/2D/western tubulin - input samples.tif]

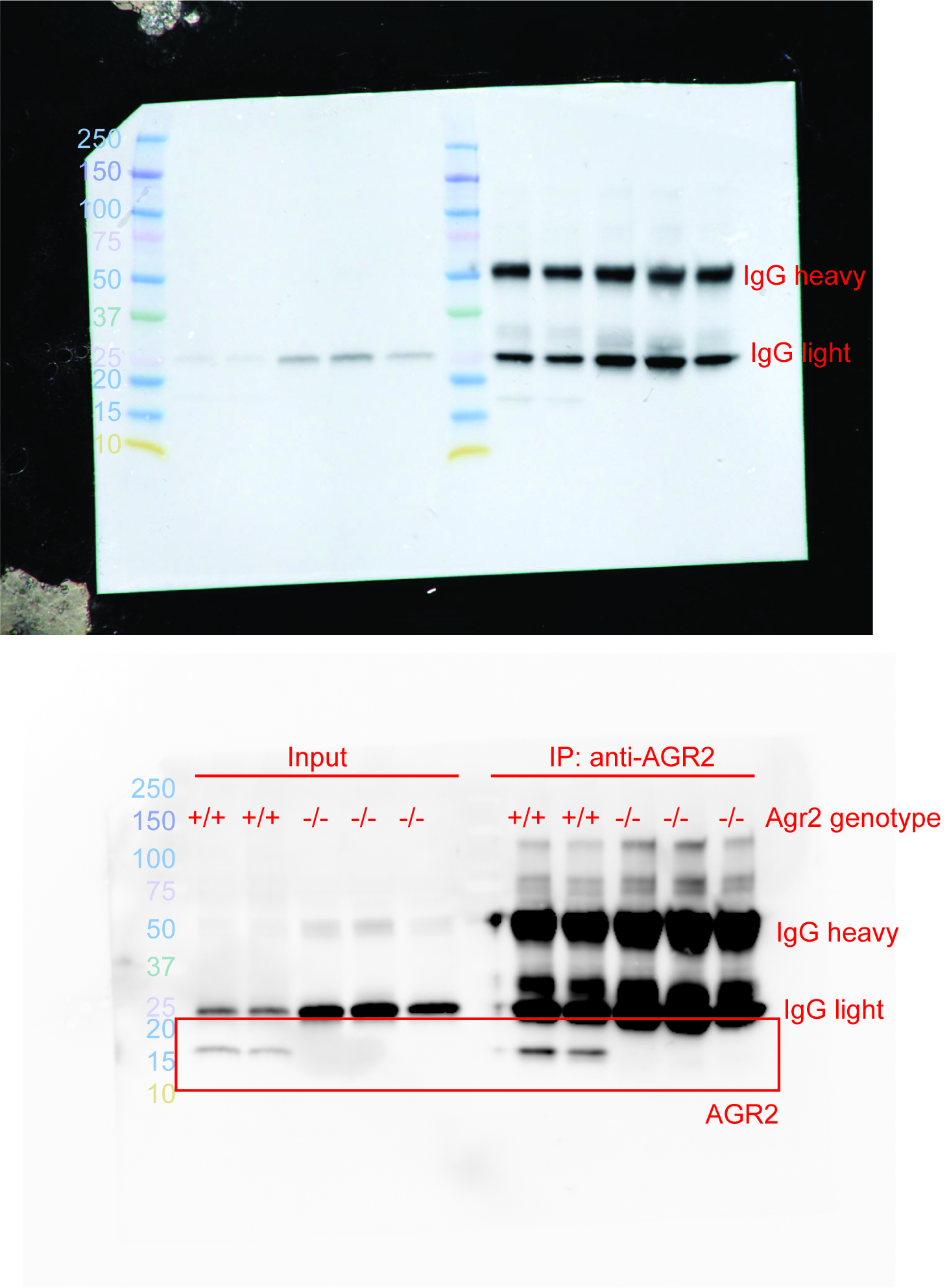

Supplement: Supplementary file 4 — Source Data Fig. 2 [file 44318_2023_15_MOESM4_ESM.zip › Figure 2/2E/Replicate/western AGR2.tif]

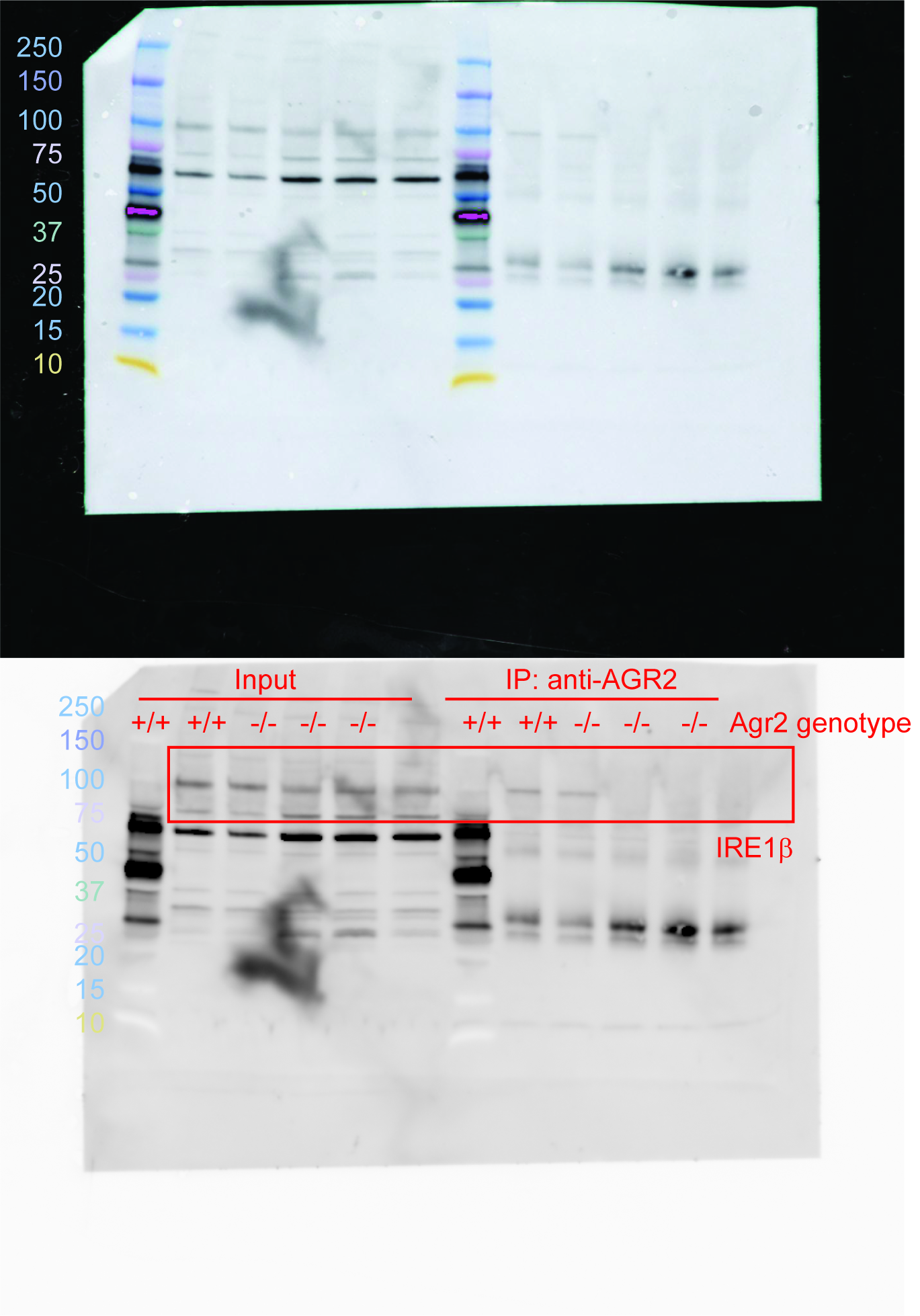

Supplement: Supplementary file 4 — Source Data Fig. 2 [file 44318_2023_15_MOESM4_ESM.zip › Figure 2/2E/Replicate/western IRE1b.tif]

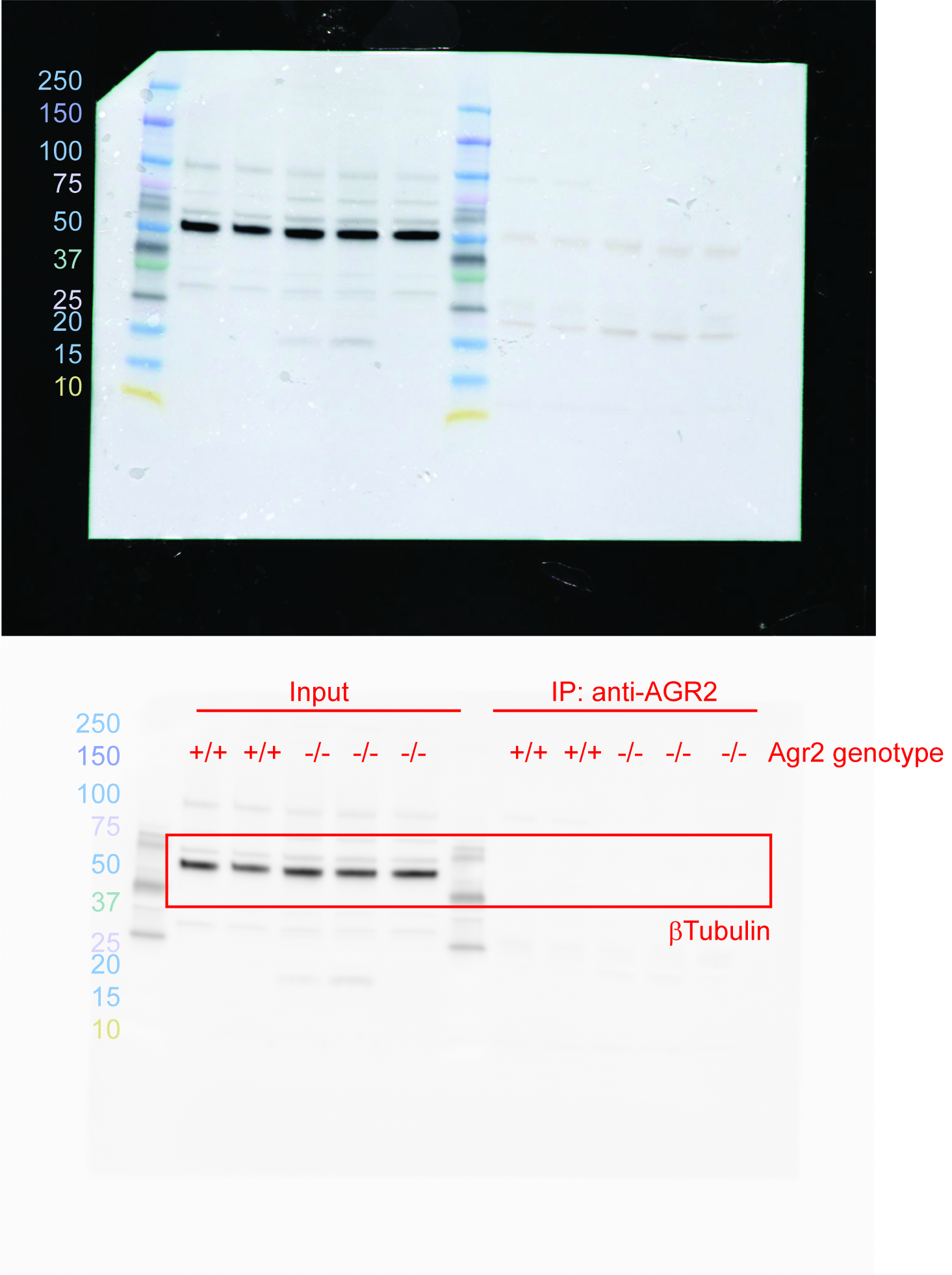

Supplement: Supplementary file 4 — Source Data Fig. 2 [file 44318_2023_15_MOESM4_ESM.zip › Figure 2/2E/Replicate/western tubulin.tif]

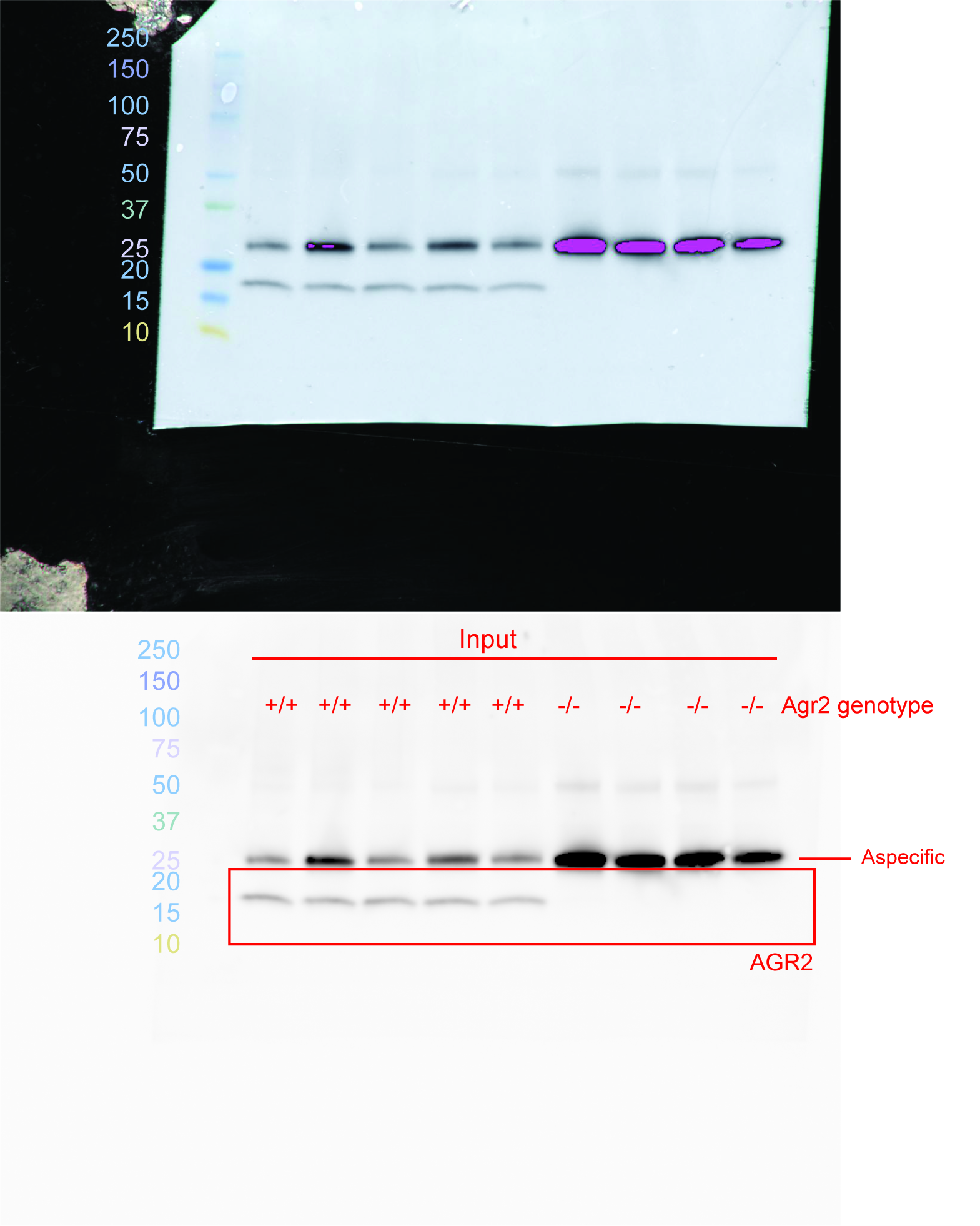

Supplement: Supplementary file 4 — Source Data Fig. 2 [file 44318_2023_15_MOESM4_ESM.zip › Figure 2/2E/western input AGR2.tif]

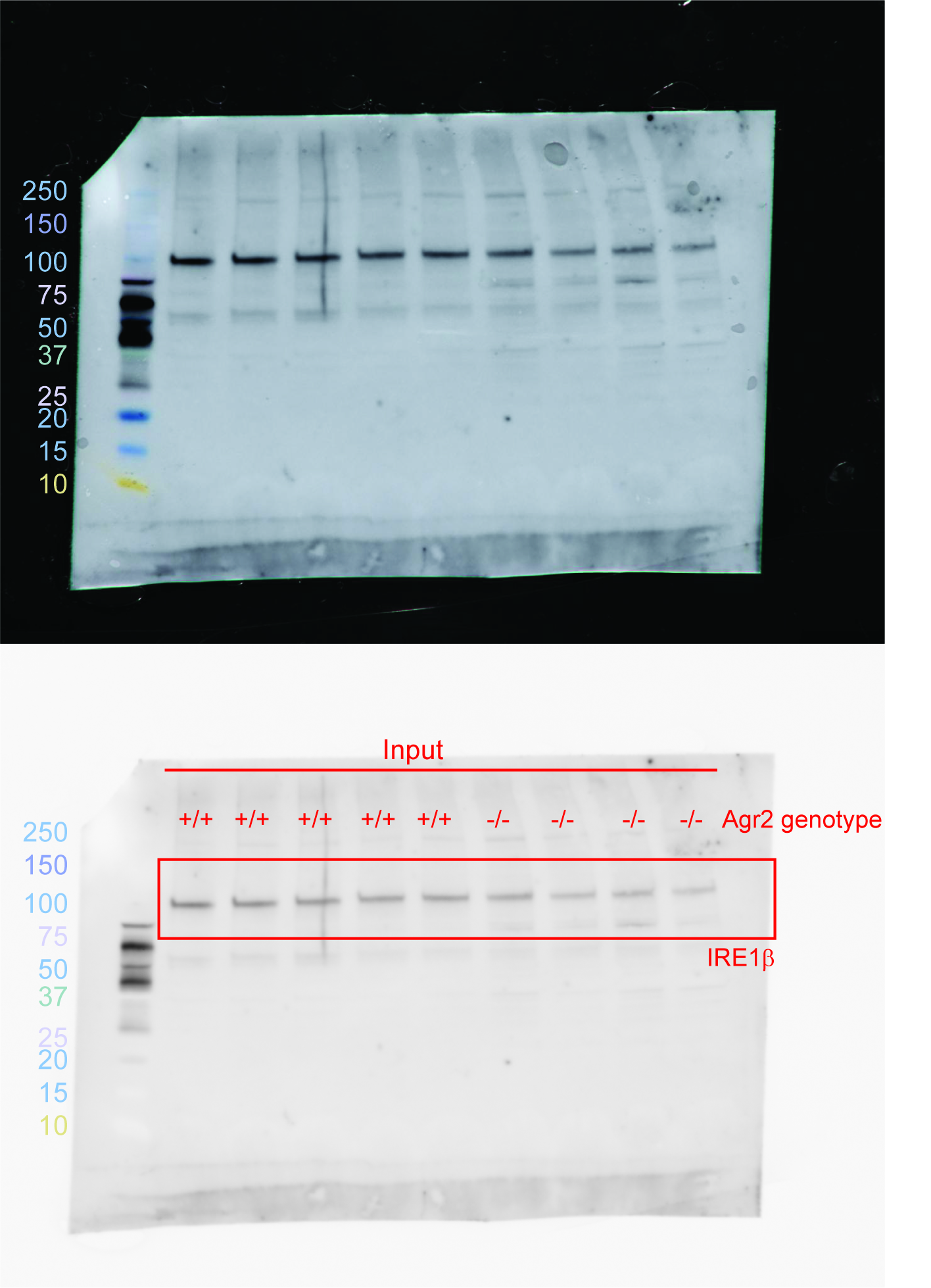

Supplement: Supplementary file 4 — Source Data Fig. 2 [file 44318_2023_15_MOESM4_ESM.zip › Figure 2/2E/western input IRE1b.tif]

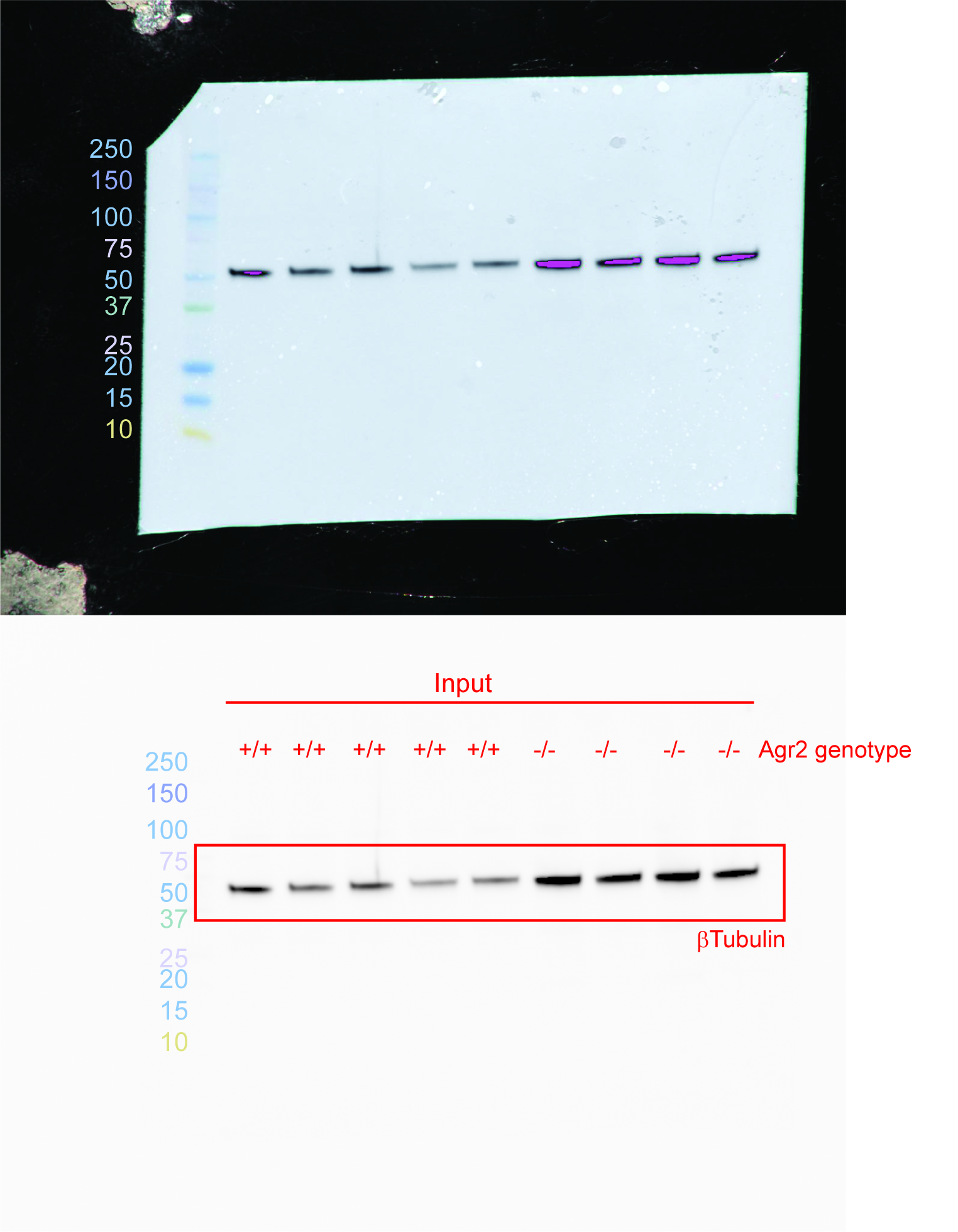

Supplement: Supplementary file 4 — Source Data Fig. 2 [file 44318_2023_15_MOESM4_ESM.zip › Figure 2/2E/western input tubulin.tif]

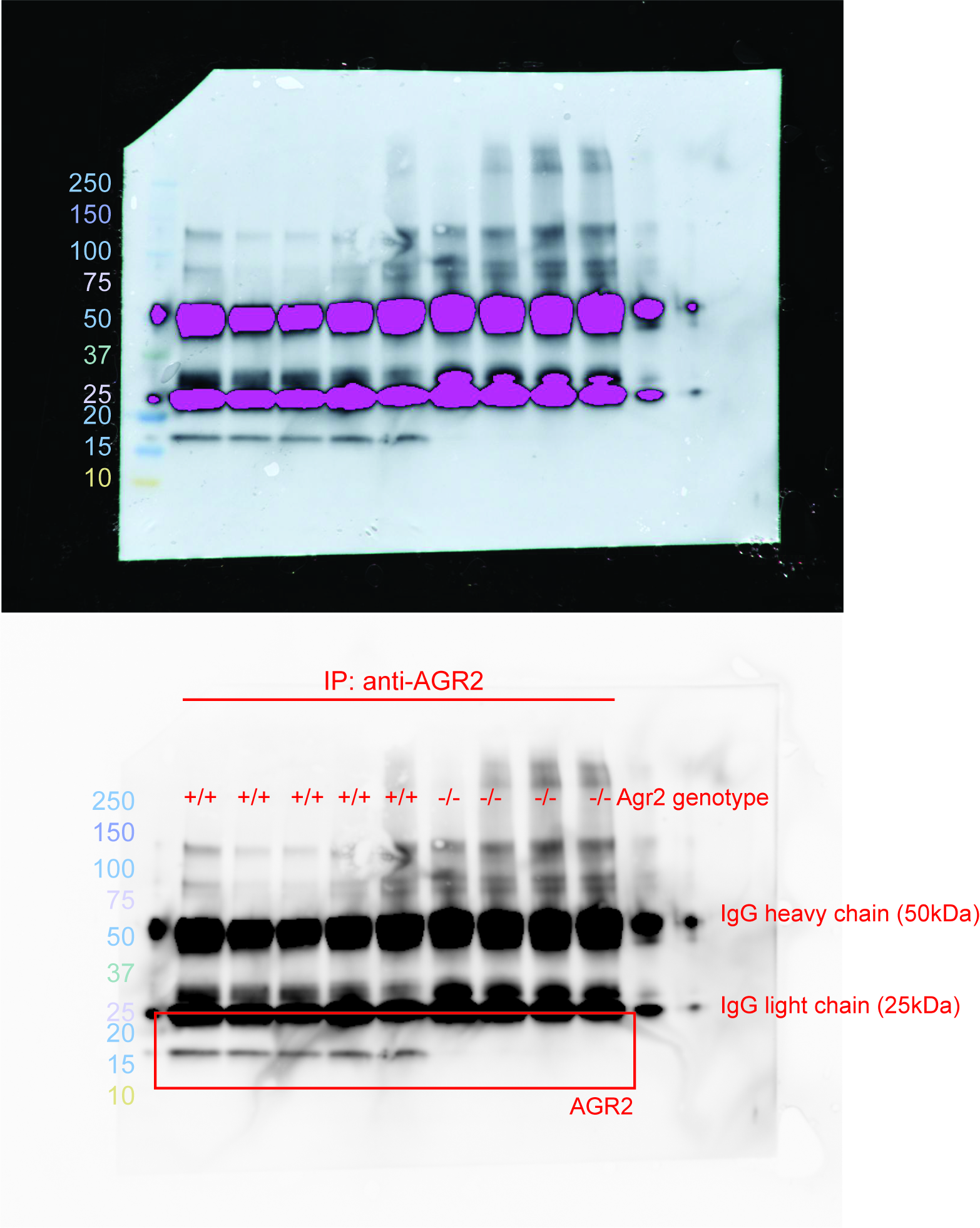

Supplement: Supplementary file 4 — Source Data Fig. 2 [file 44318_2023_15_MOESM4_ESM.zip › Figure 2/2E/western IP AGR2.tif]

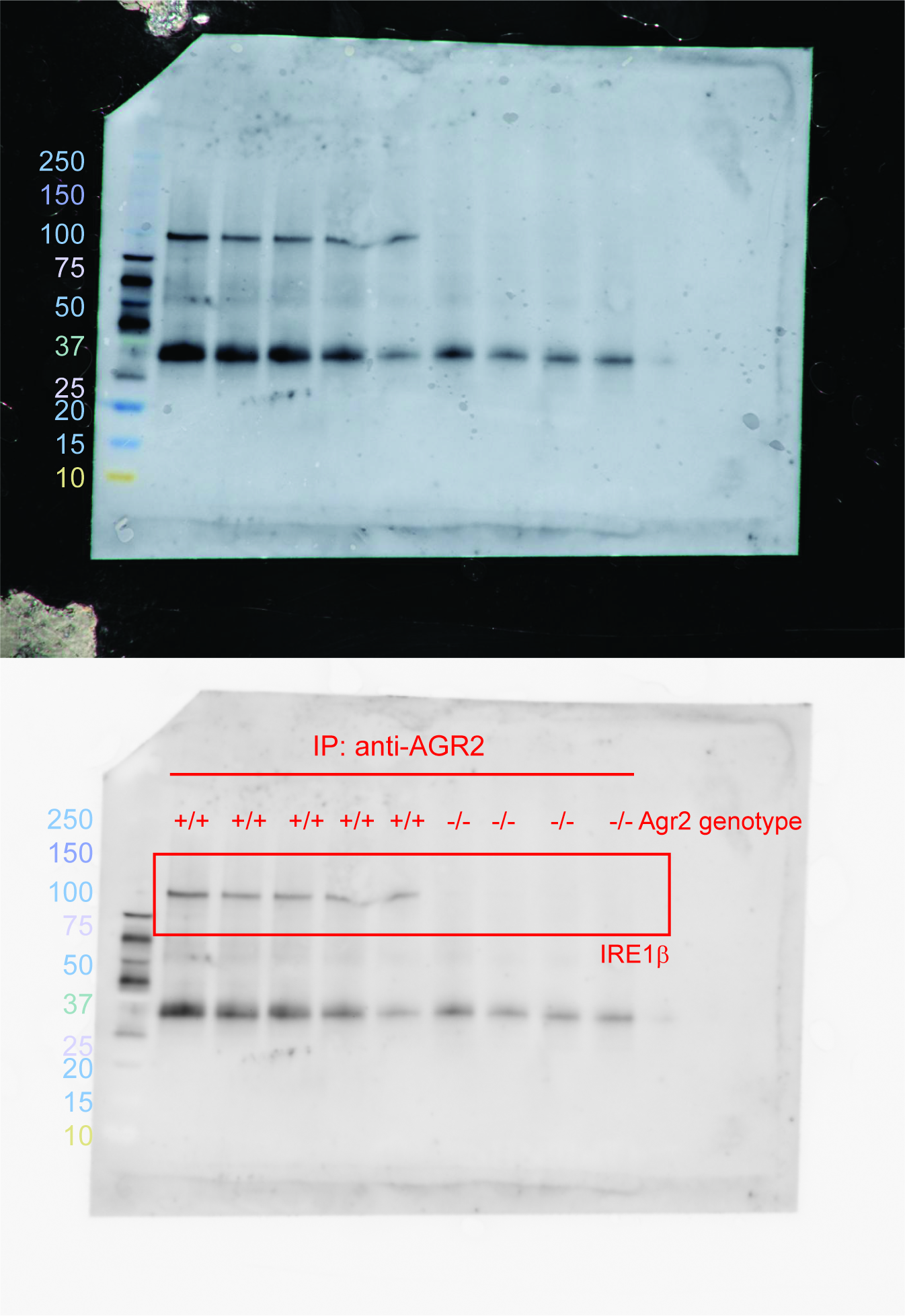

Supplement: Supplementary file 4 — Source Data Fig. 2 [file 44318_2023_15_MOESM4_ESM.zip › Figure 2/2E/western IP IRE1b.tif]

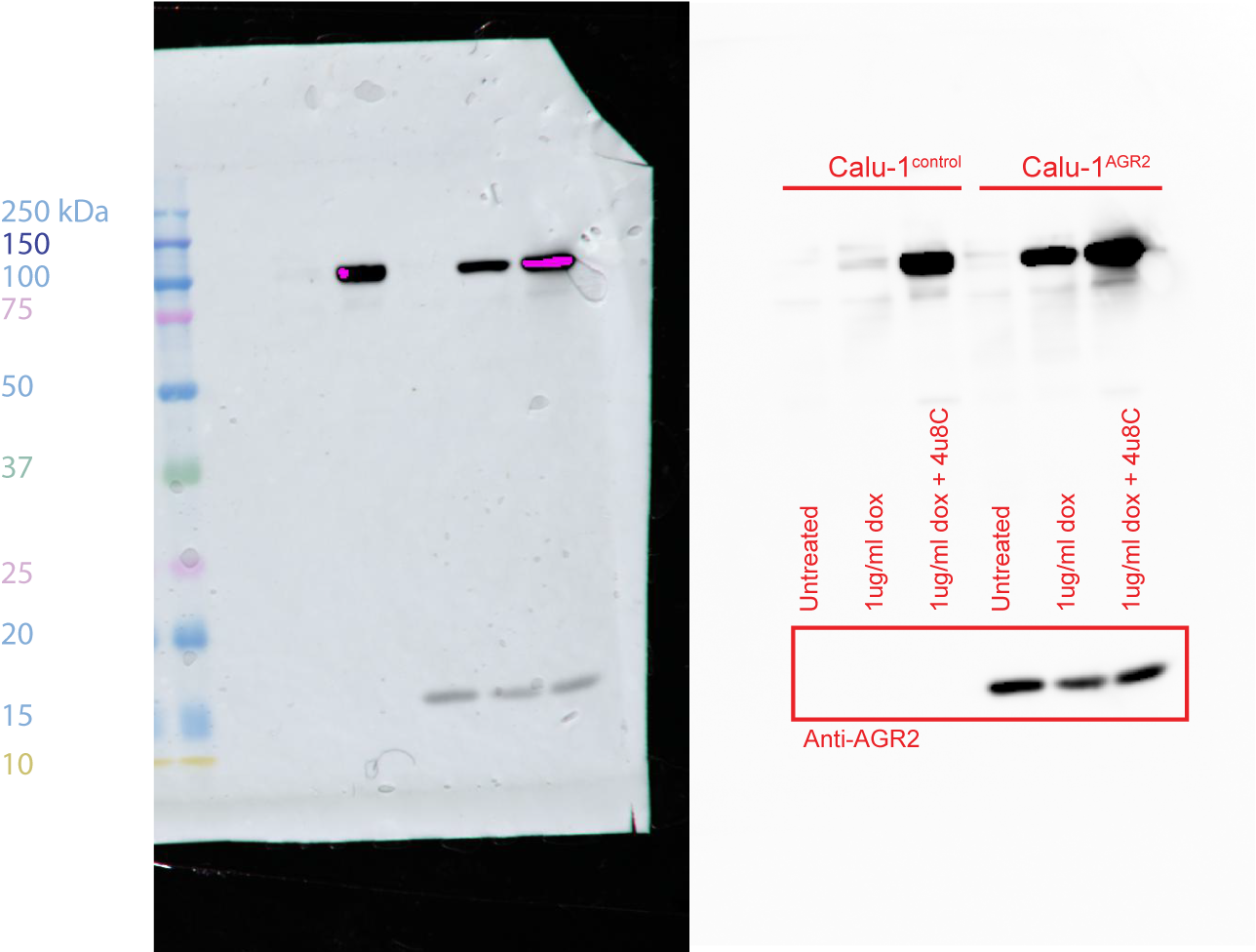

Supplement: Supplementary file 5 — Source Data Fig. 3 [file 44318_2023_15_MOESM5_ESM.zip › Figure 3/3E/Replicate/western AGR2.tif]

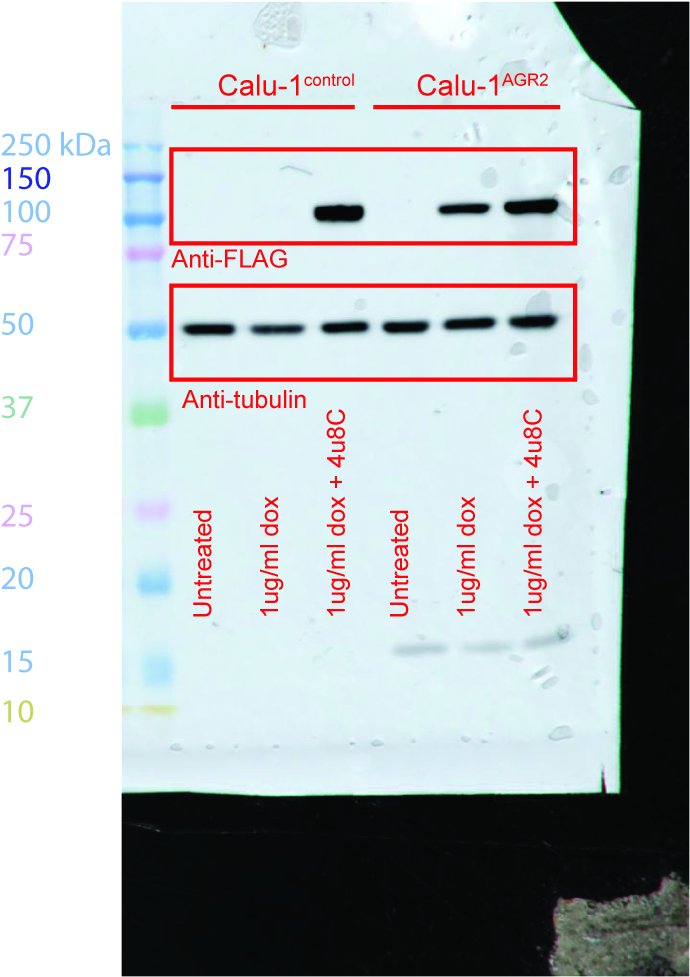

Supplement: Supplementary file 5 — Source Data Fig. 3 [file 44318_2023_15_MOESM5_ESM.zip › Figure 3/3E/Replicate/western FLAG.tif]

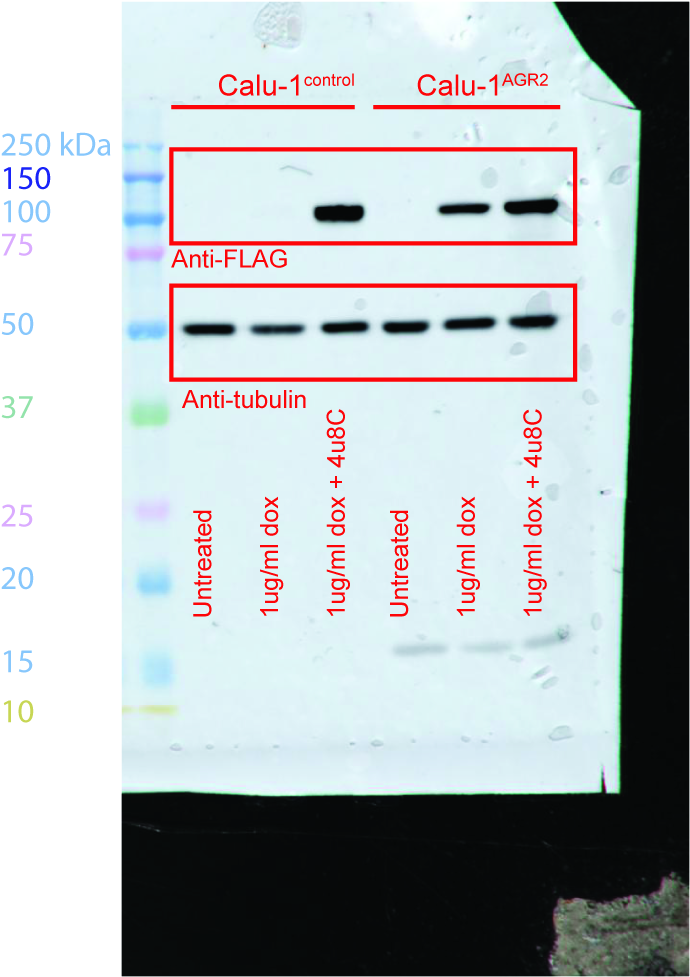

Supplement: Supplementary file 5 — Source Data Fig. 3 [file 44318_2023_15_MOESM5_ESM.zip › Figure 3/3E/Replicate/western tubulin.tif]

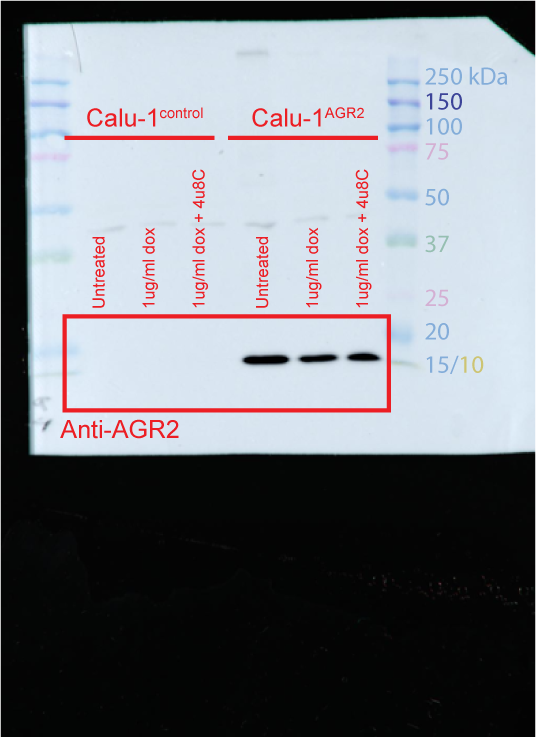

Supplement: Supplementary file 5 — Source Data Fig. 3 [file 44318_2023_15_MOESM5_ESM.zip › Figure 3/3E/western AGR2.tif]

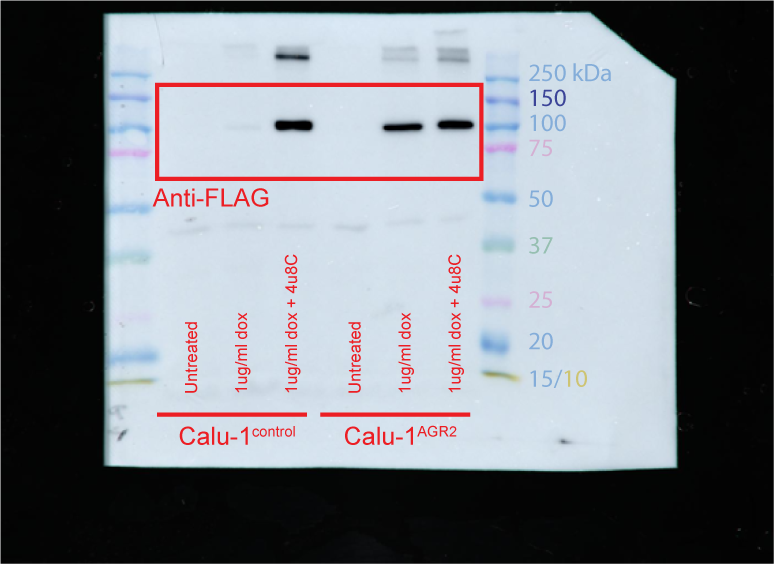

Supplement: Supplementary file 5 — Source Data Fig. 3 [file 44318_2023_15_MOESM5_ESM.zip › Figure 3/3E/western FLAG.tif]

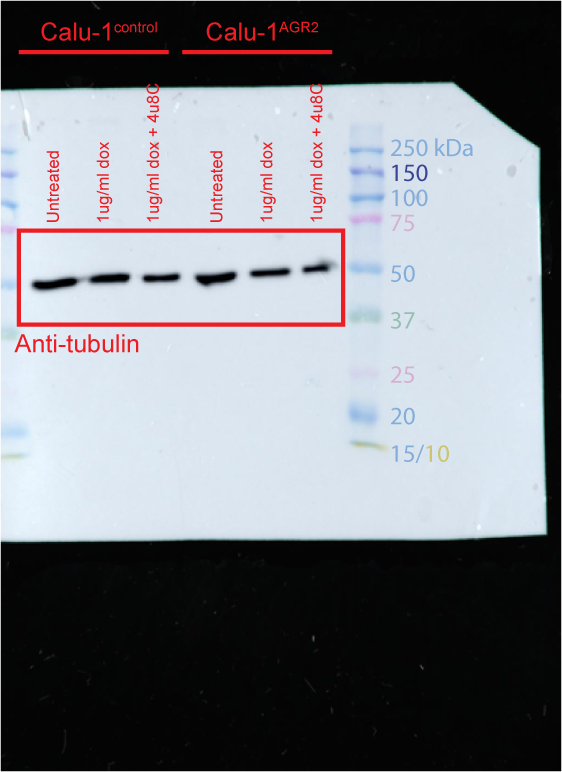

Supplement: Supplementary file 5 — Source Data Fig. 3 [file 44318_2023_15_MOESM5_ESM.zip › Figure 3/3E/western tubulin.tif]

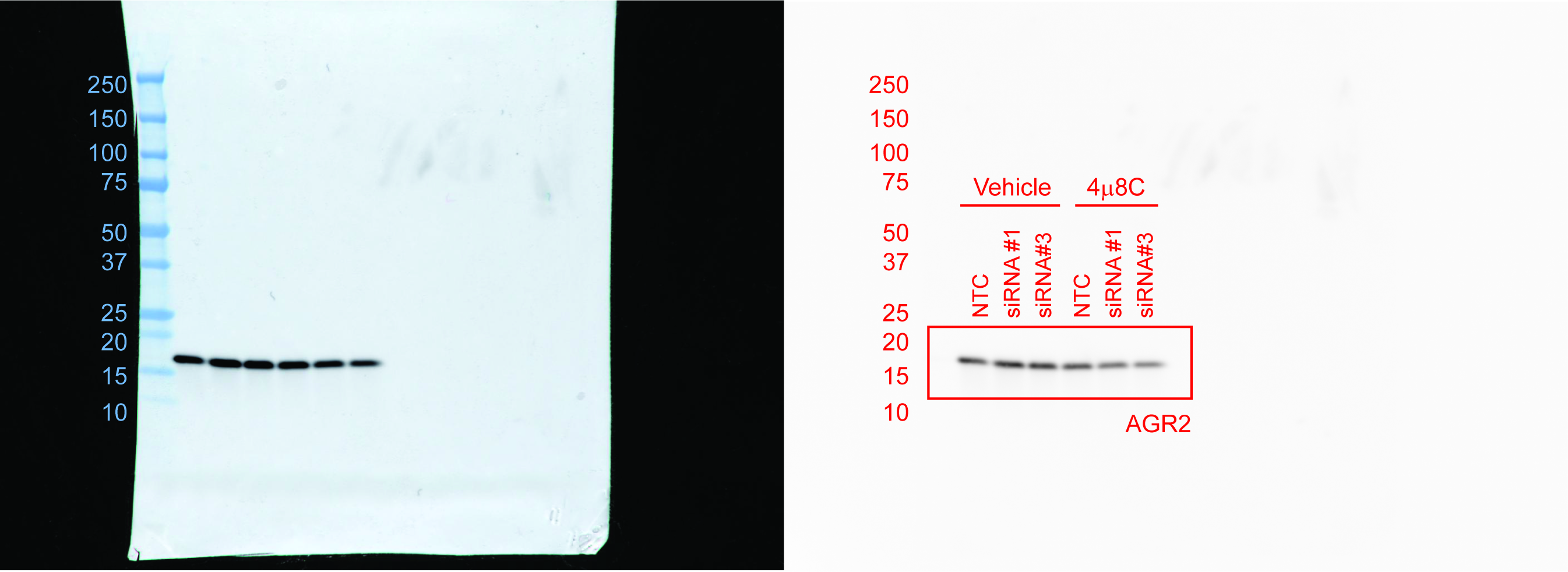

Supplement: Supplementary file 5 — Source Data Fig. 3 [file 44318_2023_15_MOESM5_ESM.zip › Figure 3/3H/replicate/western AGR2.tif]

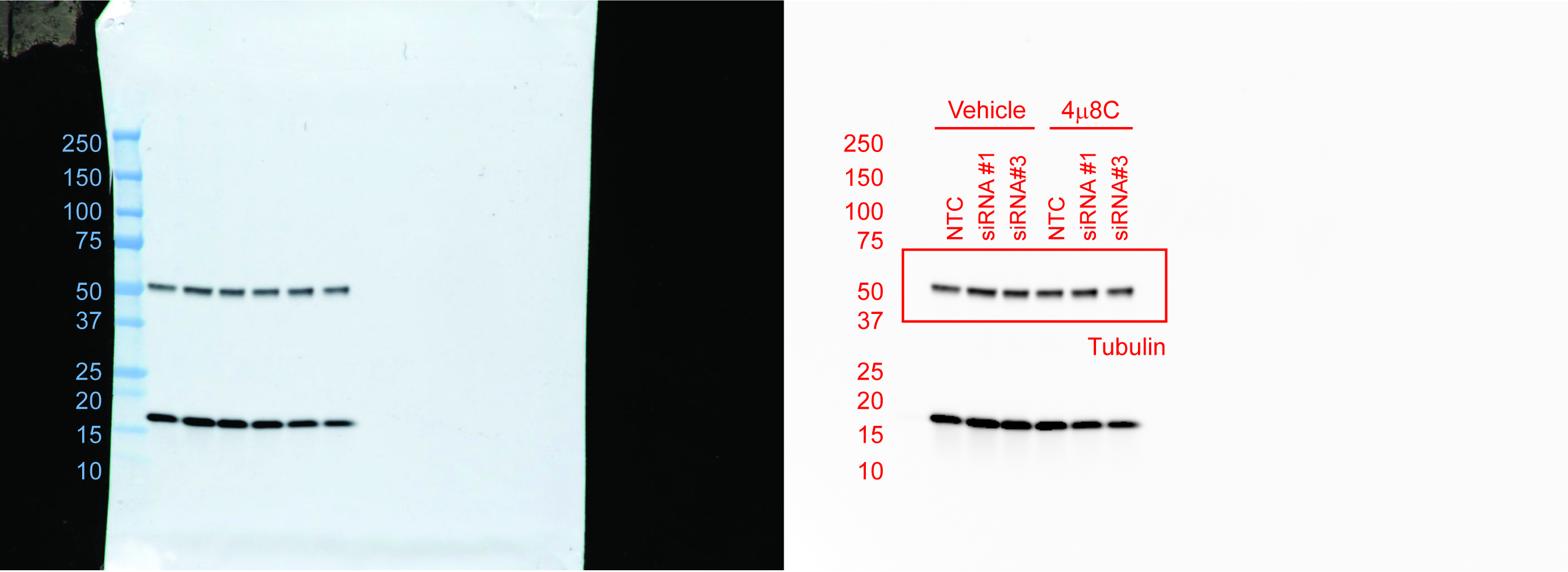

Supplement: Supplementary file 5 — Source Data Fig. 3 [file 44318_2023_15_MOESM5_ESM.zip › Figure 3/3H/replicate/western tubulin.tif]

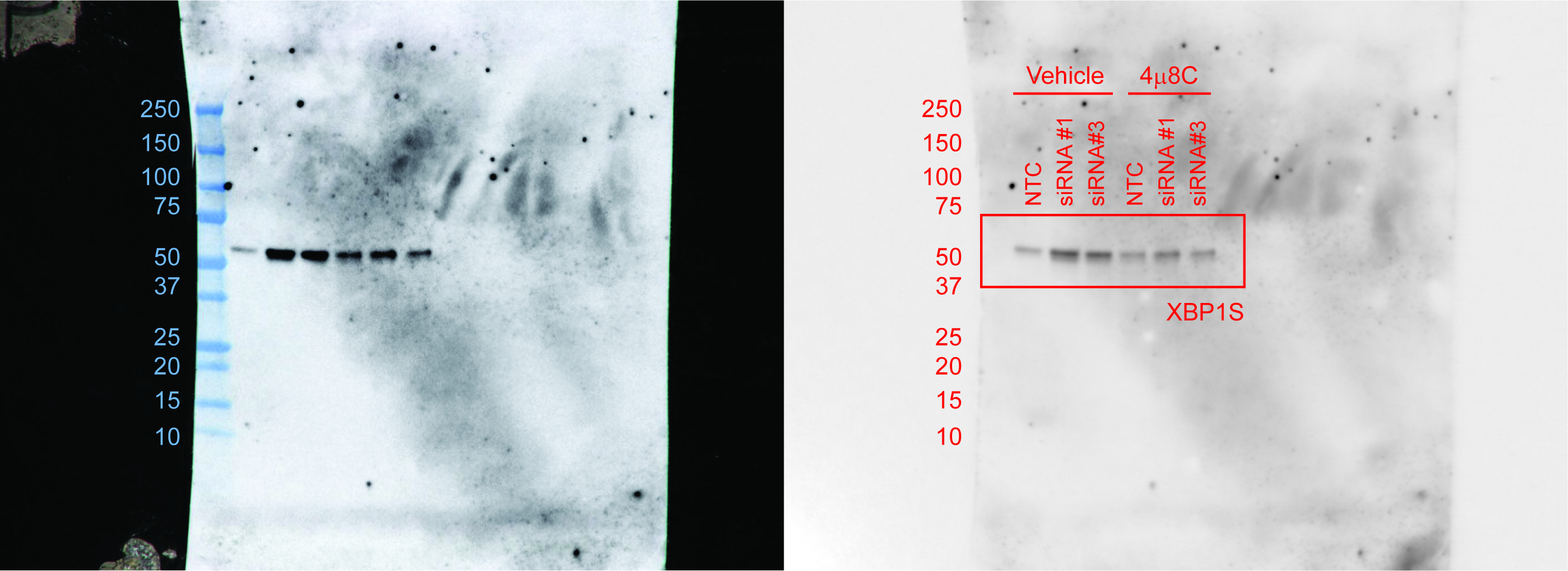

Supplement: Supplementary file 5 — Source Data Fig. 3 [file 44318_2023_15_MOESM5_ESM.zip › Figure 3/3H/replicate/western XBP1S.tif]

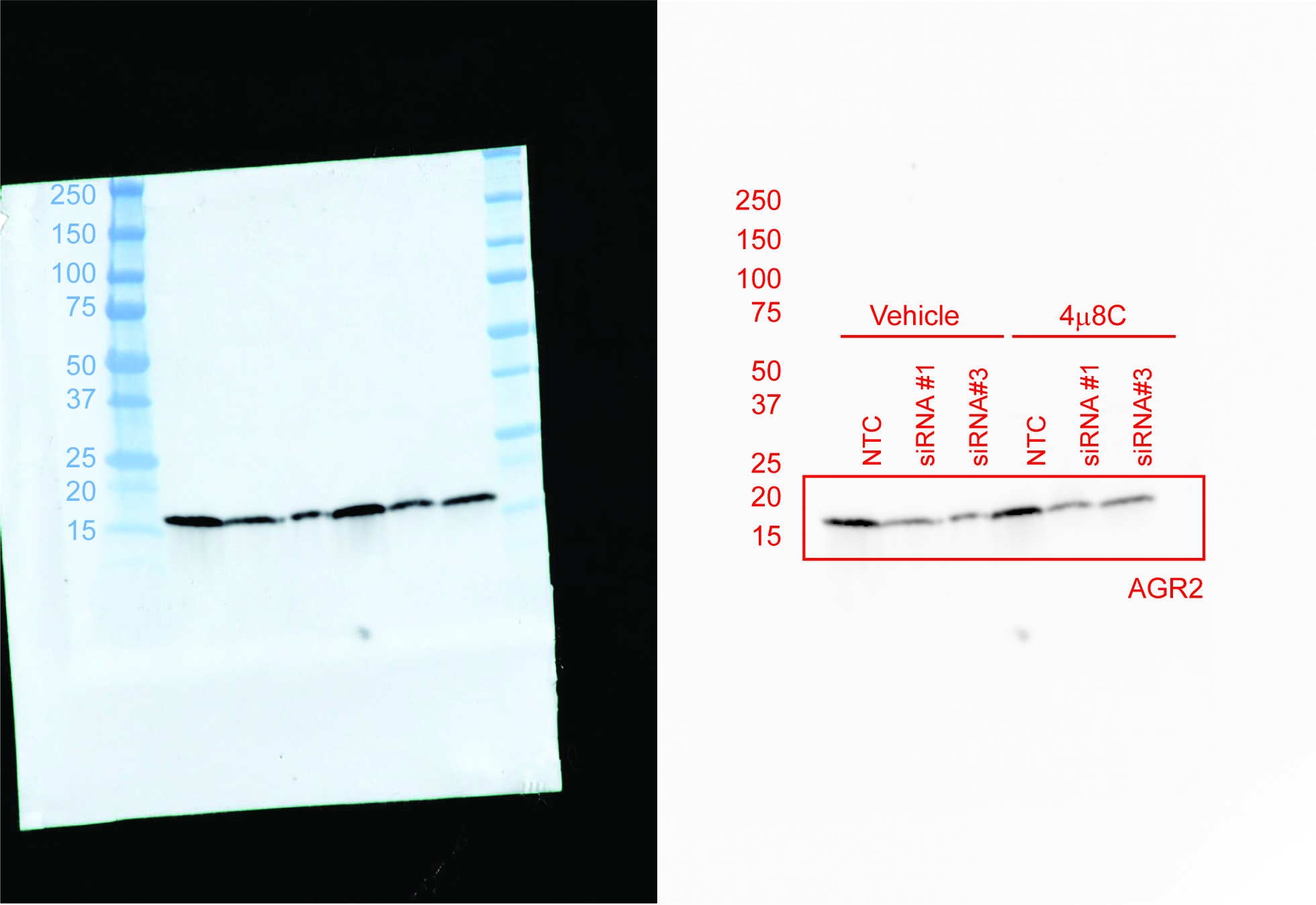

Supplement: Supplementary file 5 — Source Data Fig. 3 [file 44318_2023_15_MOESM5_ESM.zip › Figure 3/3H/western AGR2.tif]

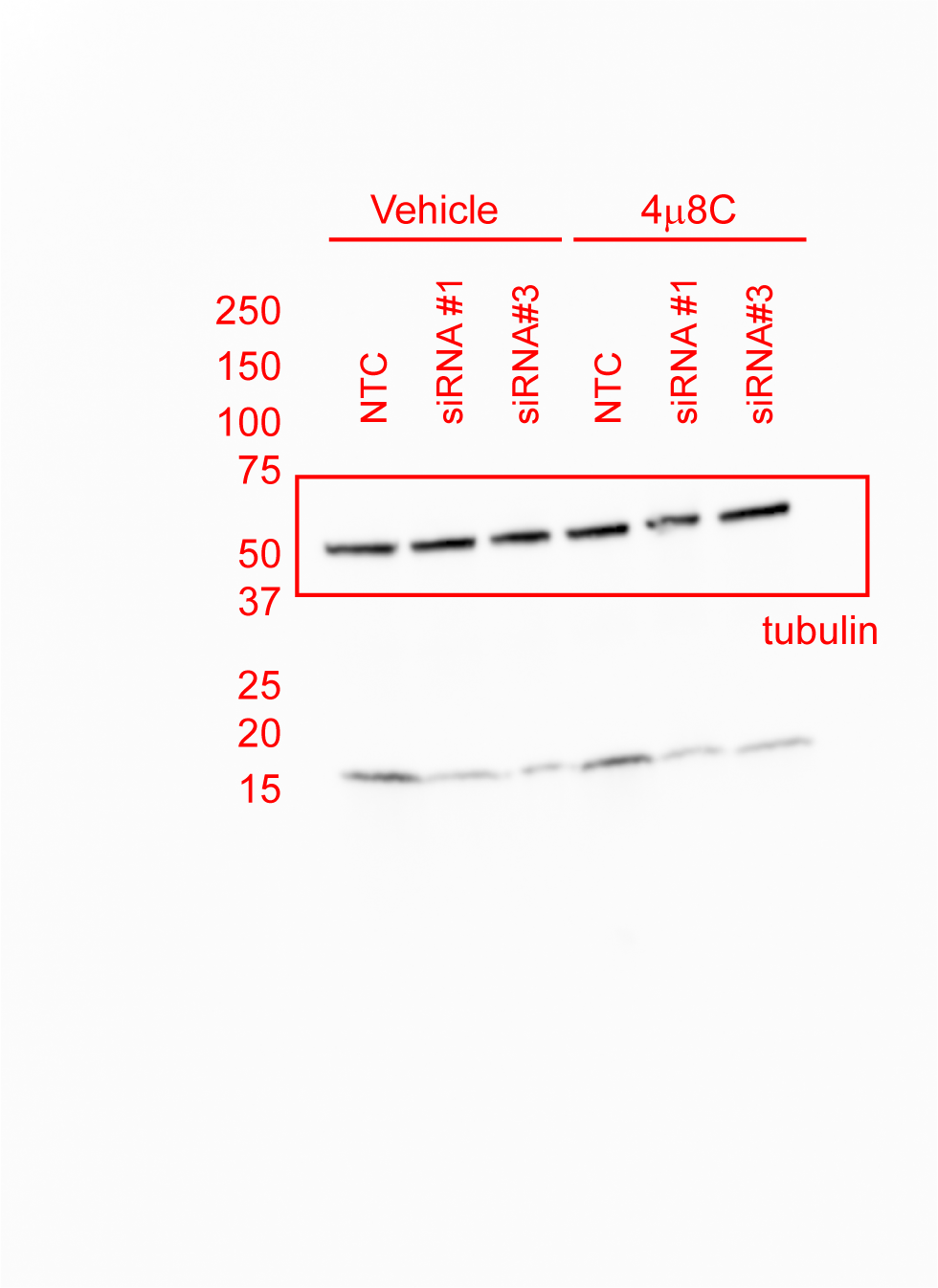

Supplement: Supplementary file 5 — Source Data Fig. 3 [file 44318_2023_15_MOESM5_ESM.zip › Figure 3/3H/western tubulin.tif]

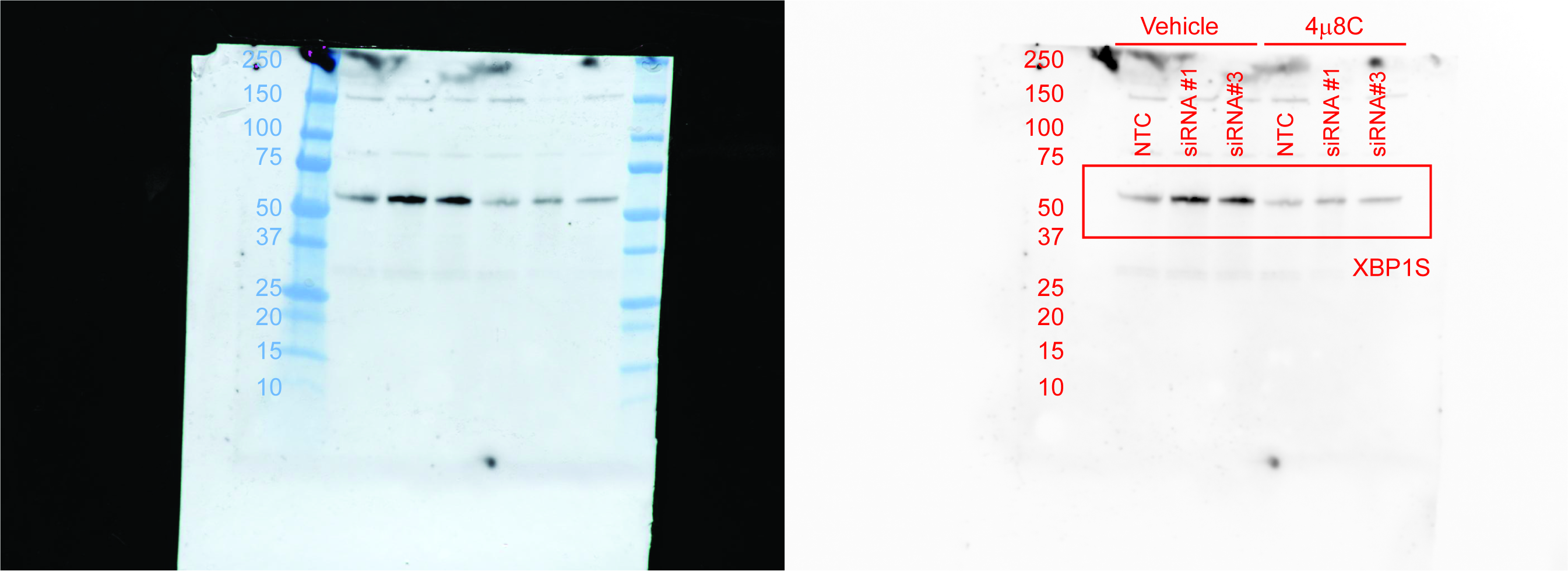

Supplement: Supplementary file 5 — Source Data Fig. 3 [file 44318_2023_15_MOESM5_ESM.zip › Figure 3/3H/western XBP1S.tif]

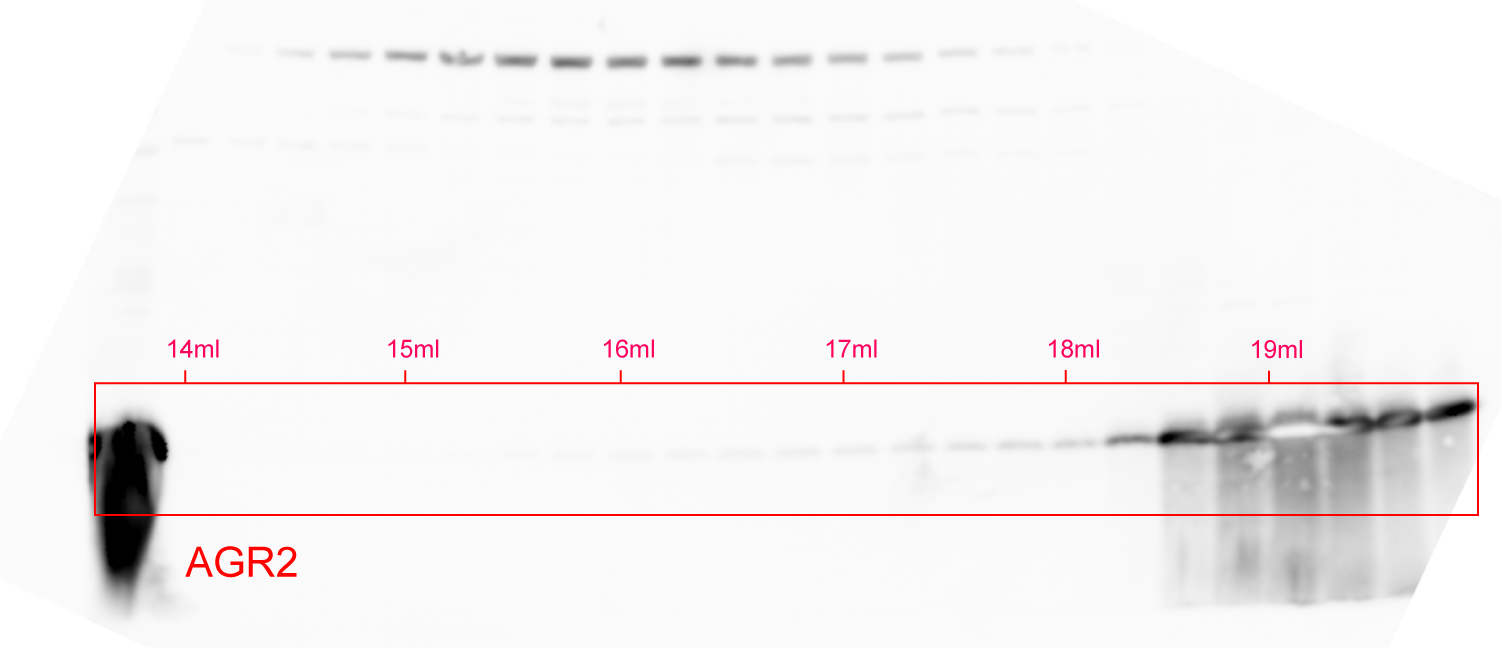

Supplement: Supplementary file 6 — Source Data Fig. 4 [file 44318_2023_15_MOESM6_ESM.zip › Figure 4/4C/Replicates/western IRE1b-agr2_AGR2_repl1.tif]

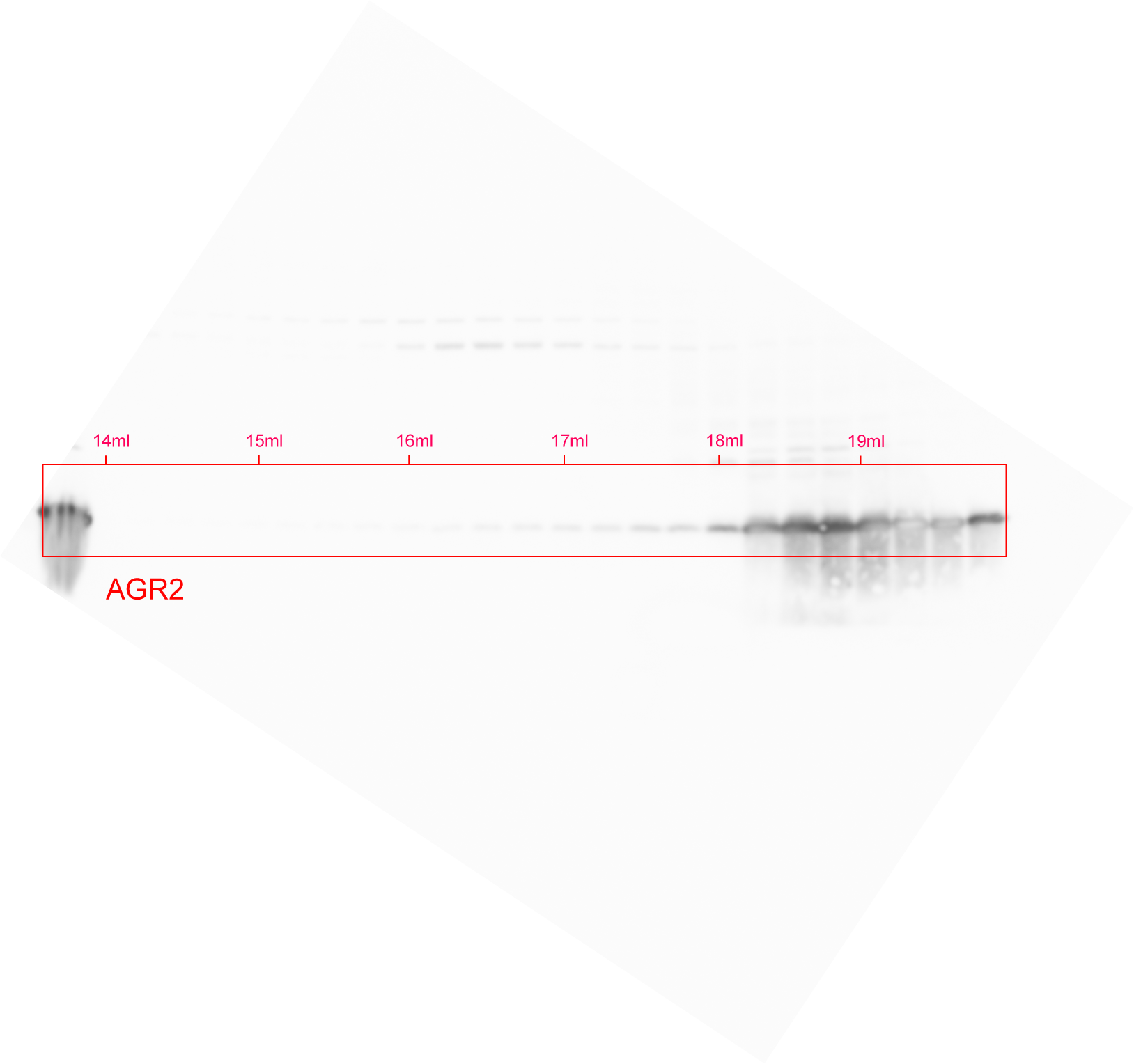

Supplement: Supplementary file 6 — Source Data Fig. 4 [file 44318_2023_15_MOESM6_ESM.zip › Figure 4/4C/Replicates/western IRE1b-agr2_AGR2_repl2.tif]

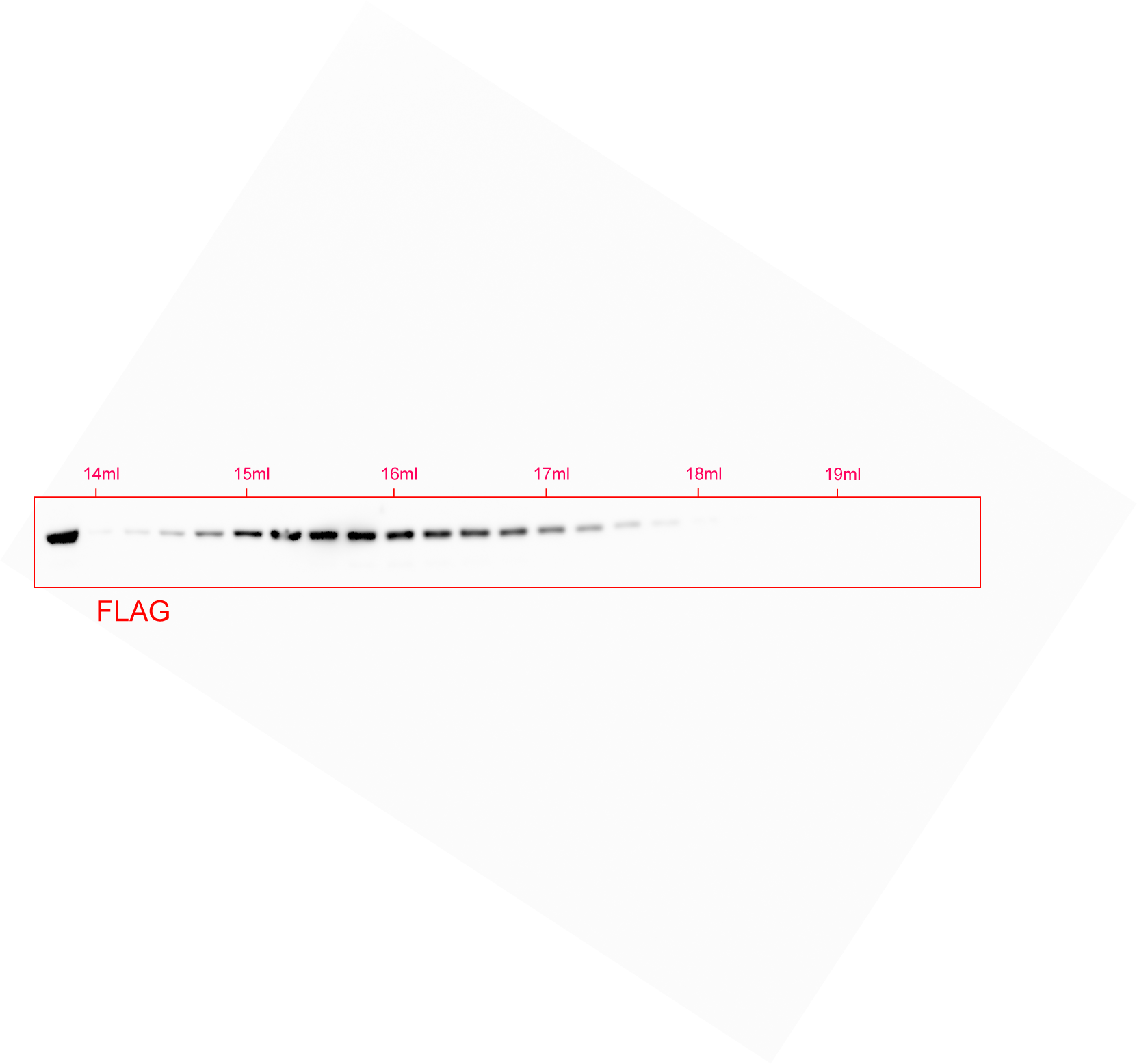

Supplement: Supplementary file 6 — Source Data Fig. 4 [file 44318_2023_15_MOESM6_ESM.zip › Figure 4/4C/Replicates/western IRE1b-agr2_FLAG_repl1.tif]

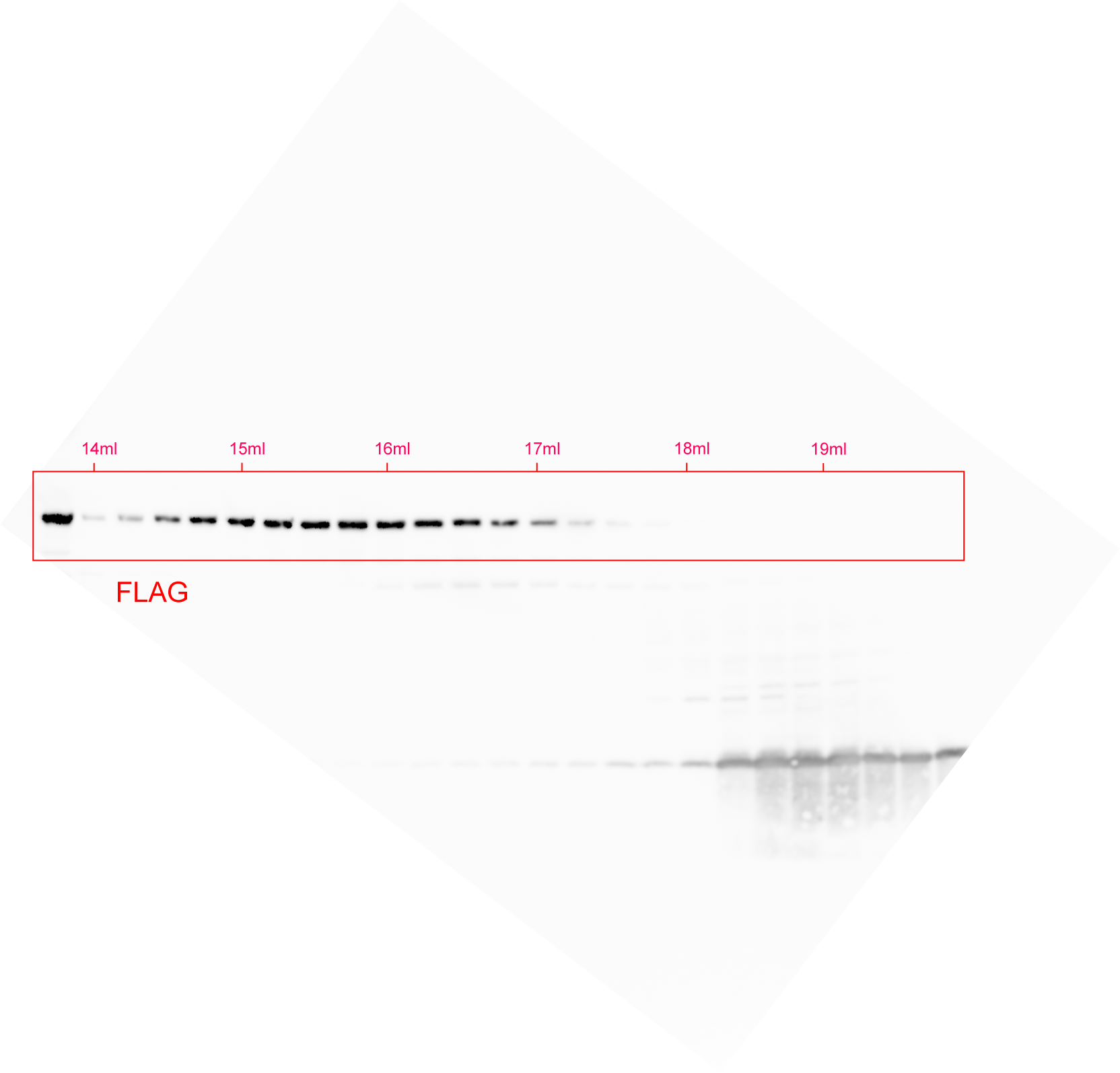

Supplement: Supplementary file 6 — Source Data Fig. 4 [file 44318_2023_15_MOESM6_ESM.zip › Figure 4/4C/Replicates/western IRE1b-agr2_FLAG_repl2.tif]

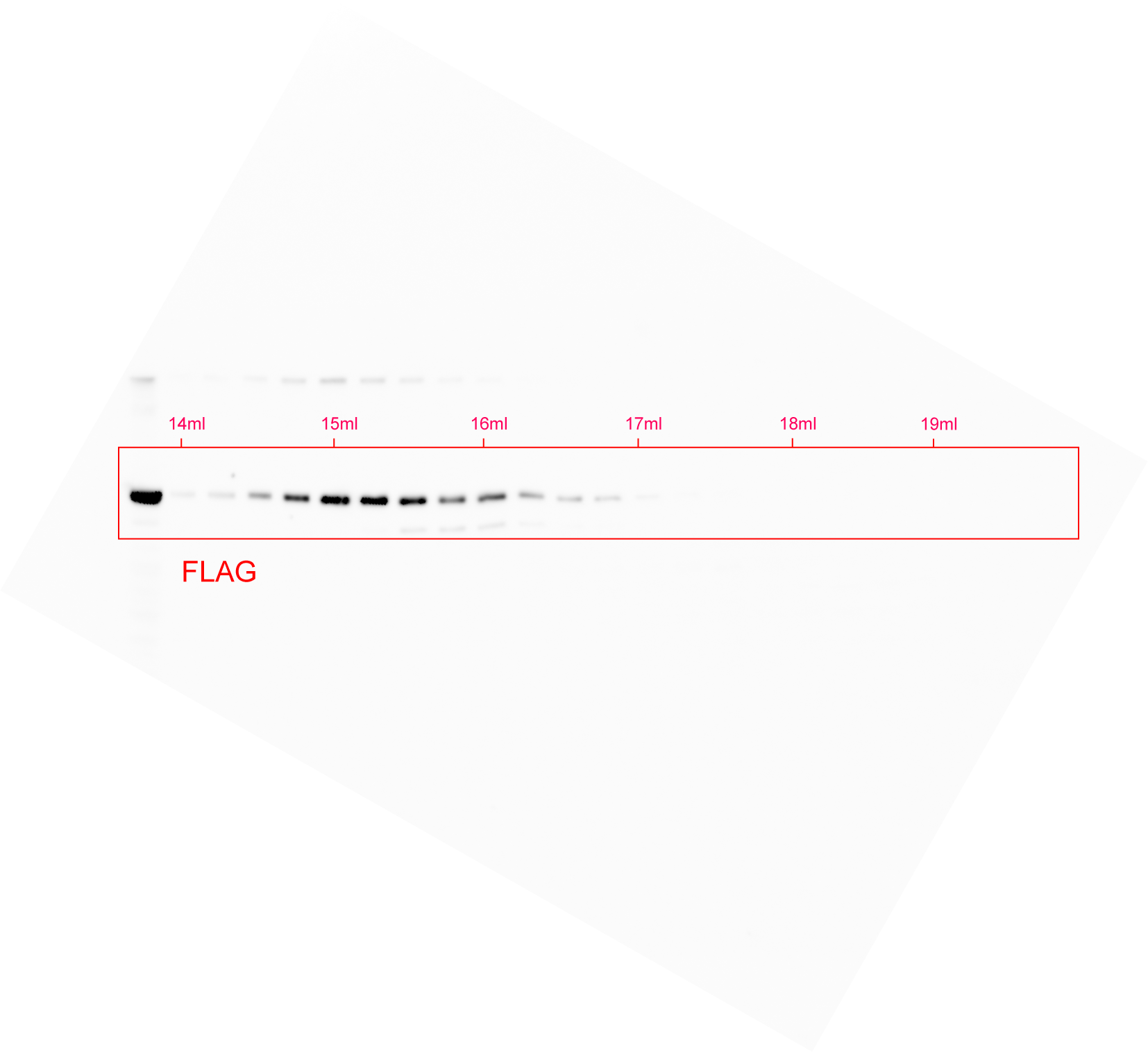

Supplement: Supplementary file 6 — Source Data Fig. 4 [file 44318_2023_15_MOESM6_ESM.zip › Figure 4/4C/Replicates/western IRE1b-control_FLAG_repl1.tif]

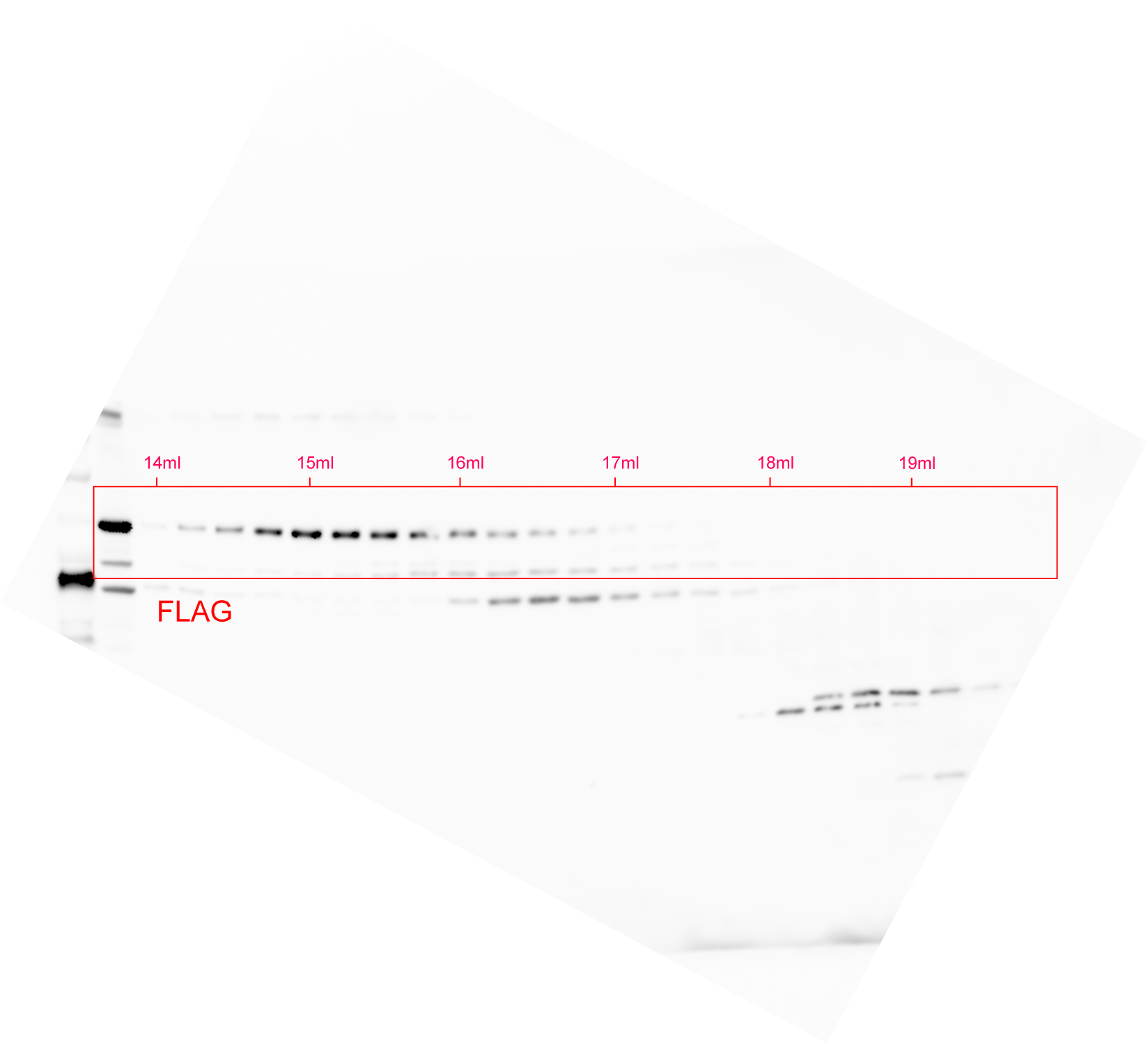

Supplement: Supplementary file 6 — Source Data Fig. 4 [file 44318_2023_15_MOESM6_ESM.zip › Figure 4/4C/Replicates/western IRE1b-control_FLAG_repl2.tif]

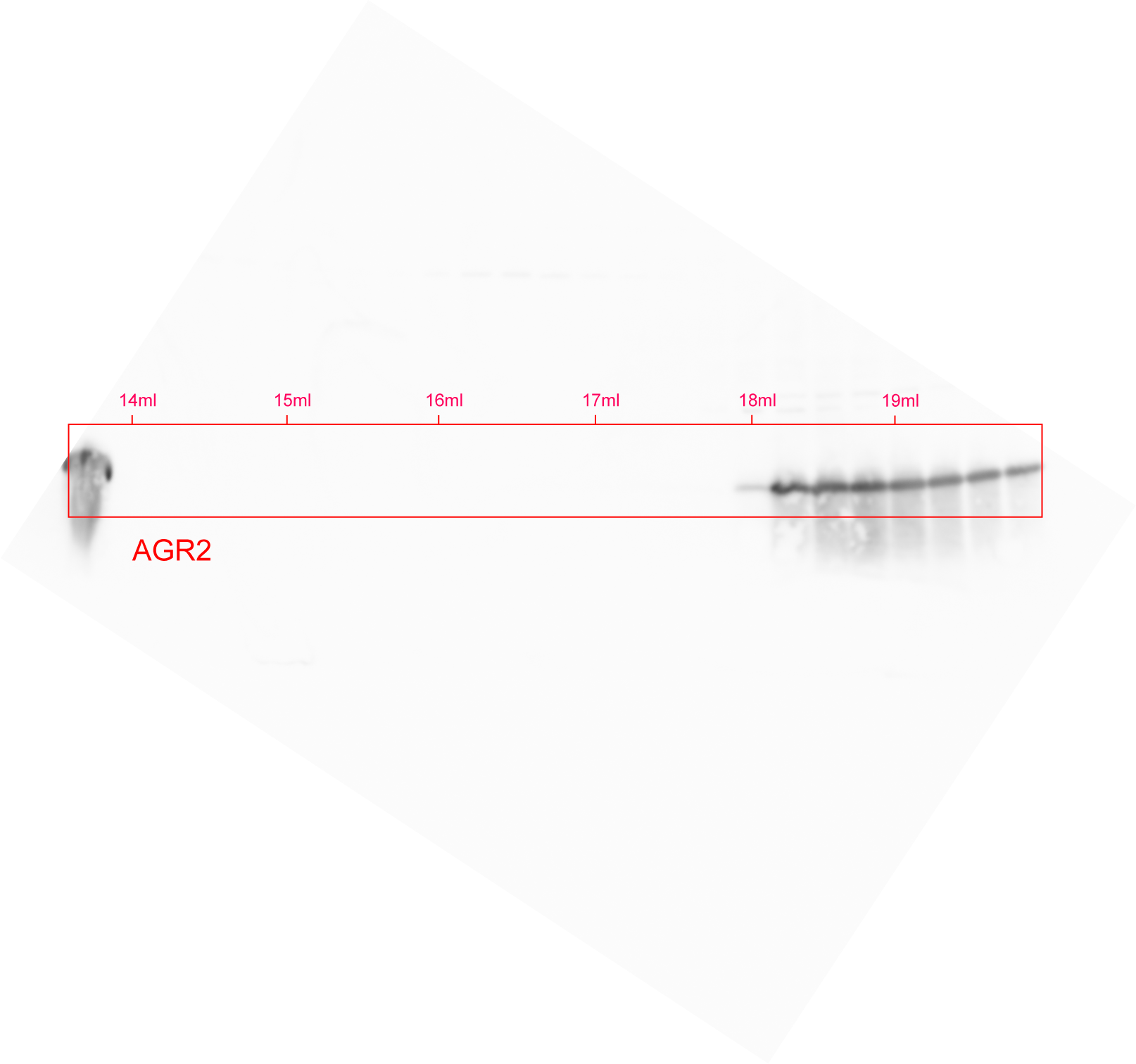

Supplement: Supplementary file 6 — Source Data Fig. 4 [file 44318_2023_15_MOESM6_ESM.zip › Figure 4/4C/Replicates/western no IRE1b-agr2_AGR2_repl.tif]

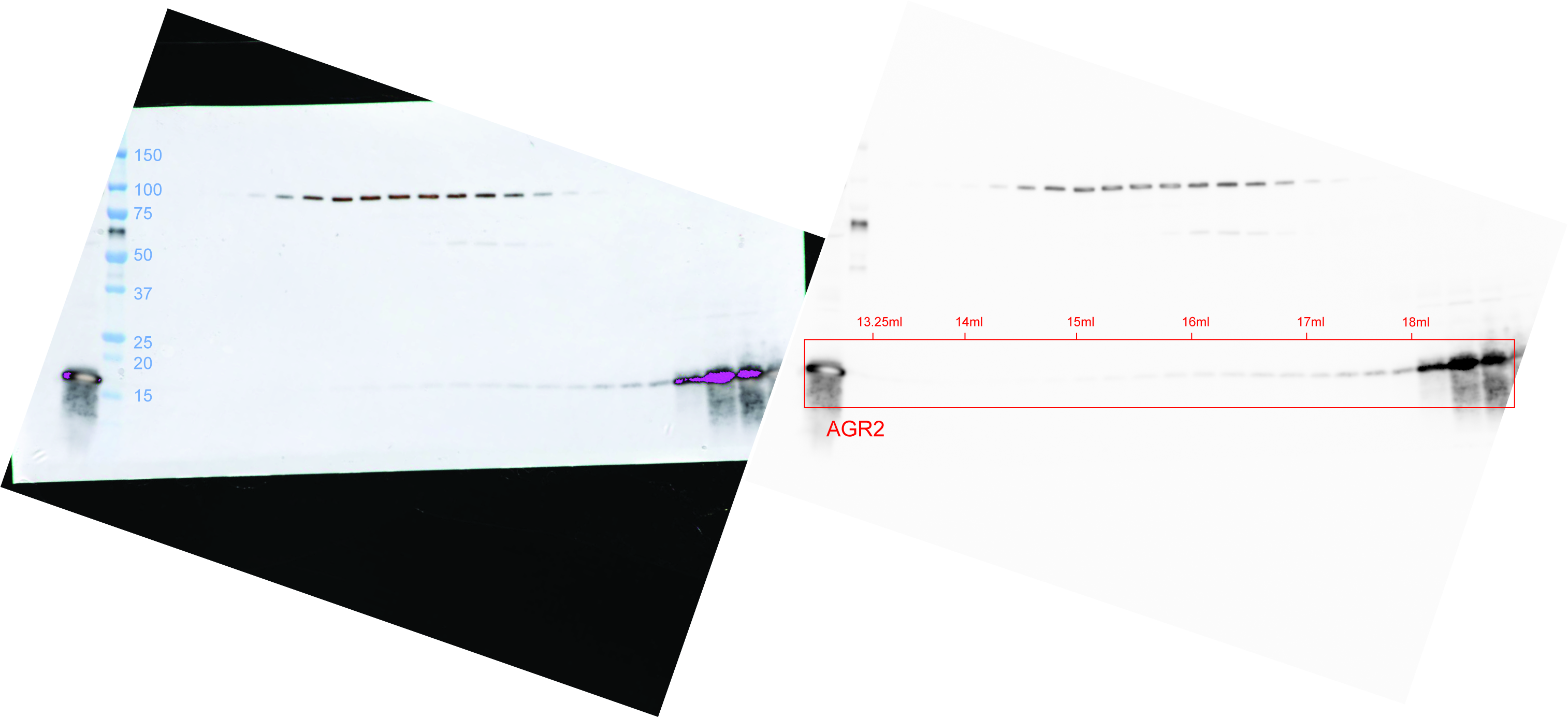

Supplement: Supplementary file 6 — Source Data Fig. 4 [file 44318_2023_15_MOESM6_ESM.zip › Figure 4/4C/western IRE1b-agr2_AGR2.tif]

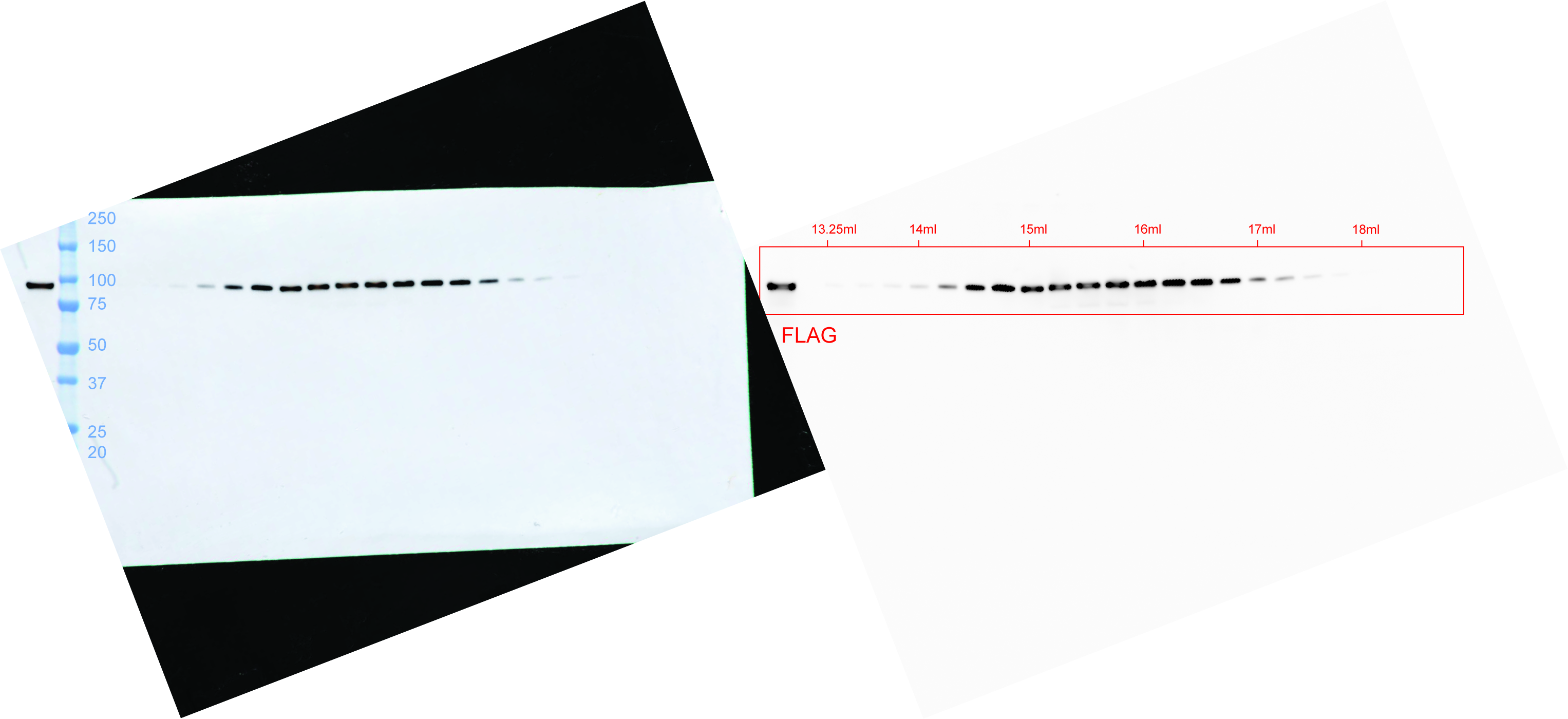

Supplement: Supplementary file 6 — Source Data Fig. 4 [file 44318_2023_15_MOESM6_ESM.zip › Figure 4/4C/western IRE1b-agr2_FLAG.tif]

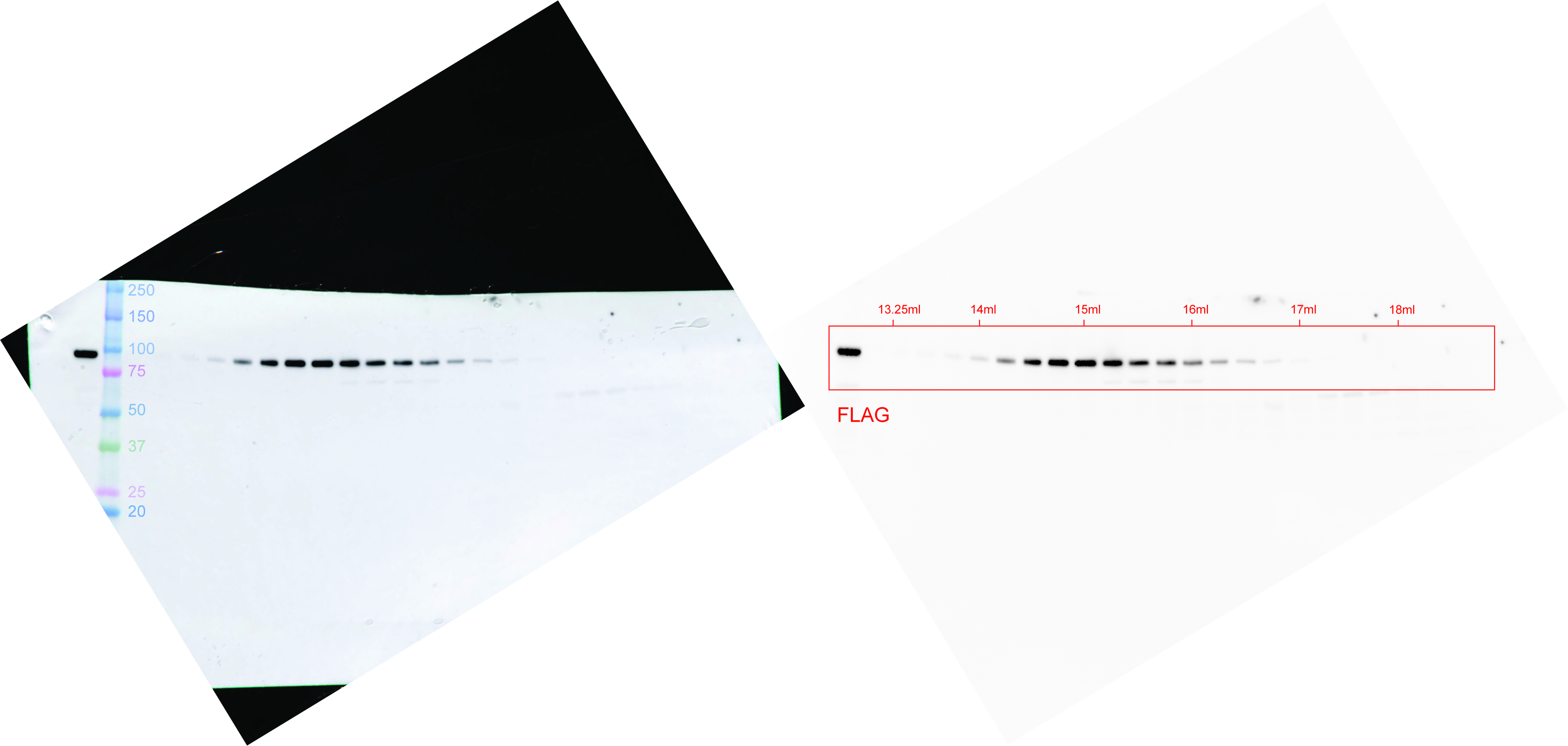

Supplement: Supplementary file 6 — Source Data Fig. 4 [file 44318_2023_15_MOESM6_ESM.zip › Figure 4/4C/western IRE1b-control_FLAG.tif]

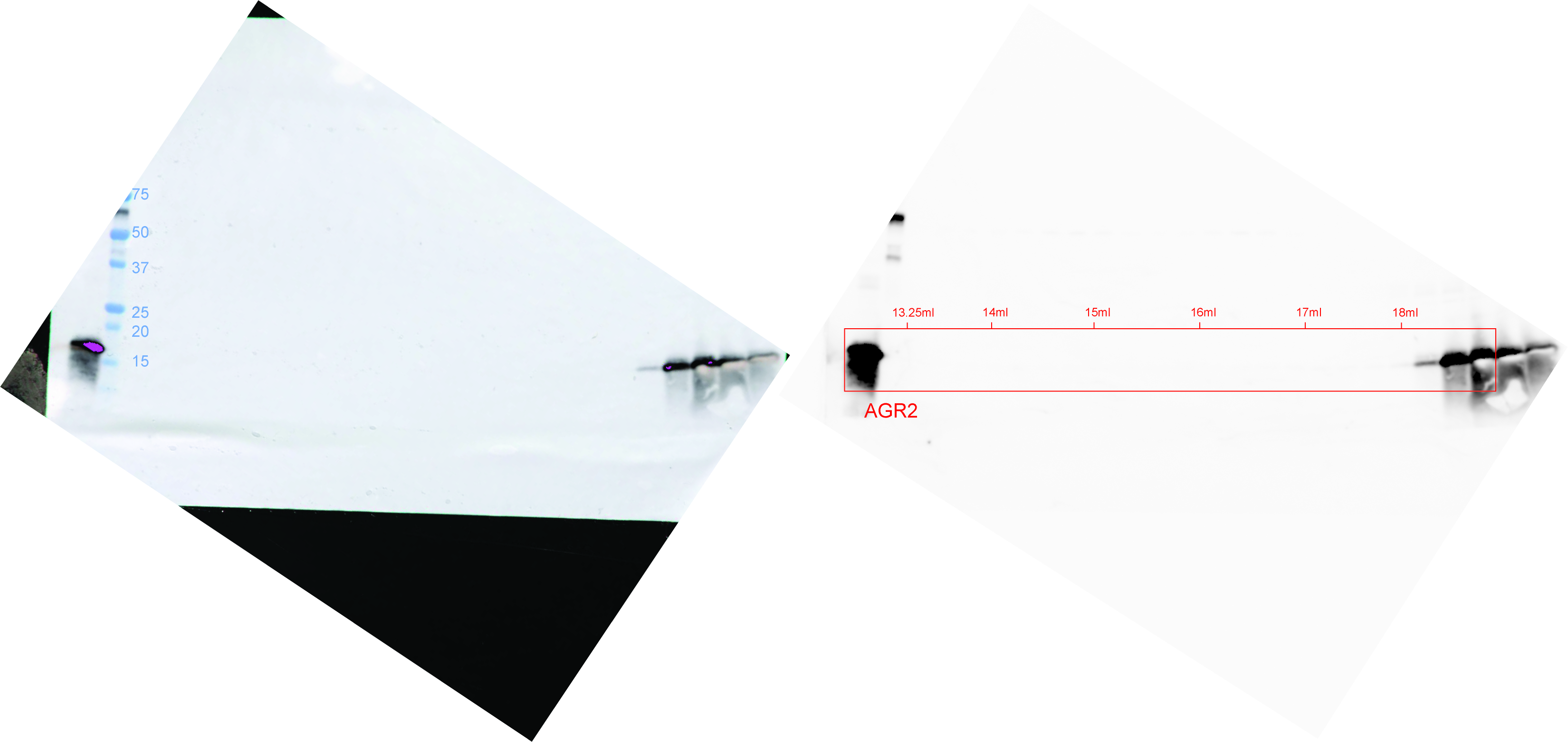

Supplement: Supplementary file 6 — Source Data Fig. 4 [file 44318_2023_15_MOESM6_ESM.zip › Figure 4/4C/western no IRE1b-agr2_AGR2.tif]

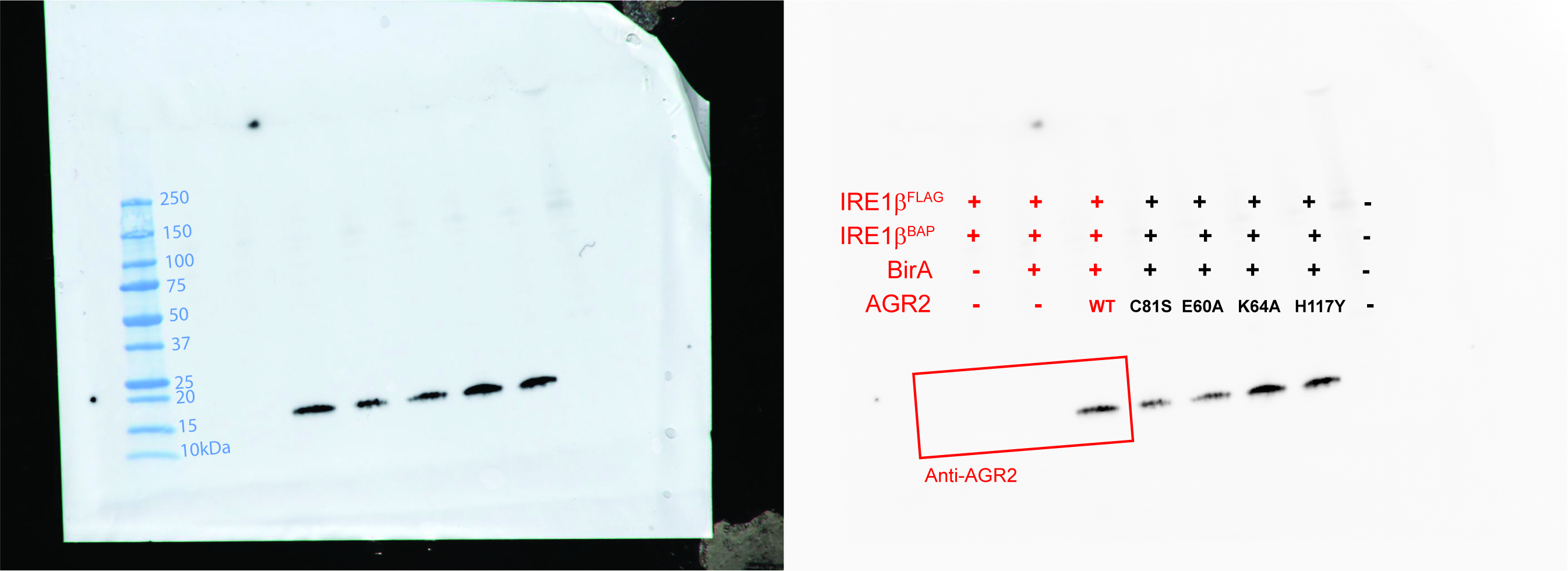

Supplement: Supplementary file 6 — Source Data Fig. 4 [file 44318_2023_15_MOESM6_ESM.zip › Figure 4/4E/western AGR2 - input samples.tif]

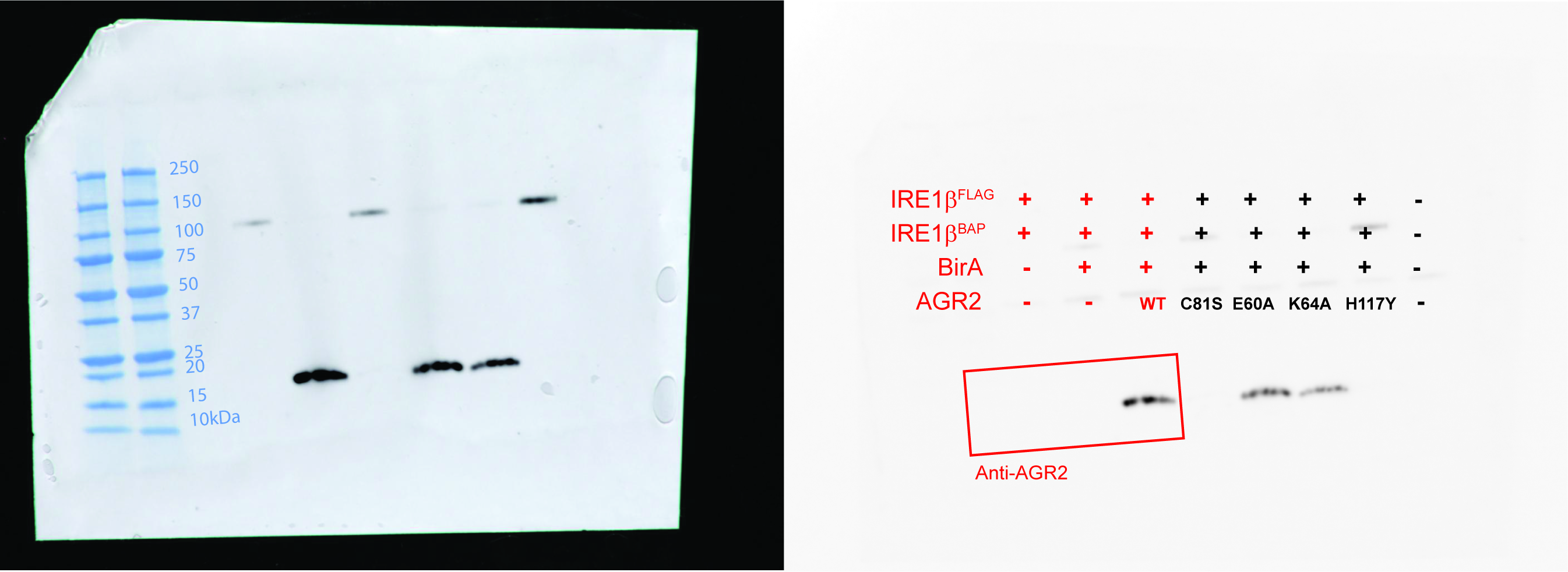

Supplement: Supplementary file 6 — Source Data Fig. 4 [file 44318_2023_15_MOESM6_ESM.zip › Figure 4/4E/western AGR2 - IP samples.tif]

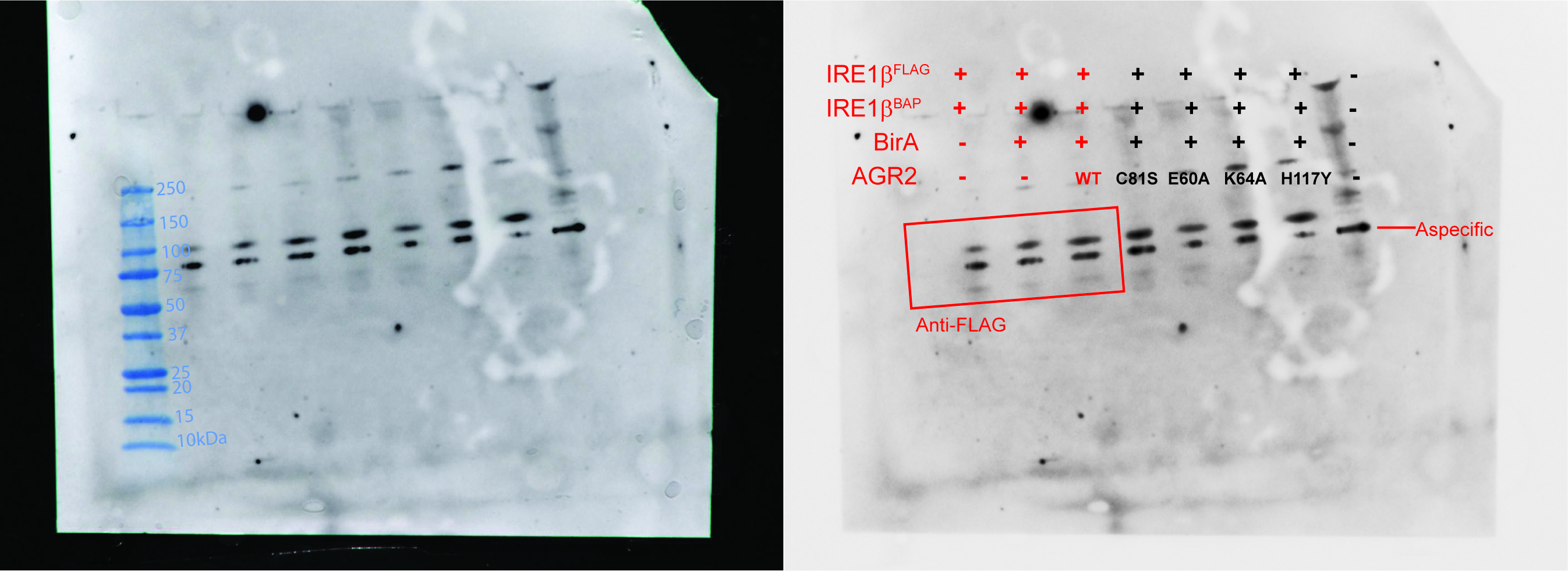

Supplement: Supplementary file 6 — Source Data Fig. 4 [file 44318_2023_15_MOESM6_ESM.zip › Figure 4/4E/western FLAG - input samples.tif]

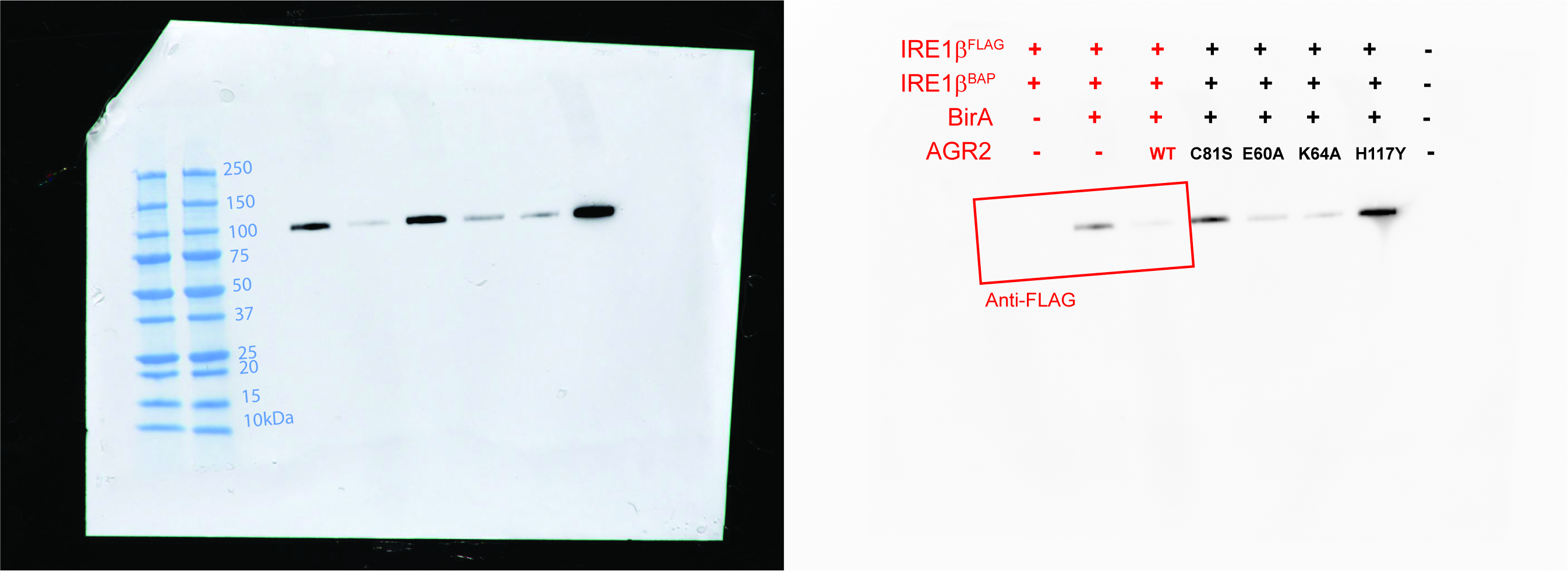

Supplement: Supplementary file 6 — Source Data Fig. 4 [file 44318_2023_15_MOESM6_ESM.zip › Figure 4/4E/western FLAG - IP samples.tif]

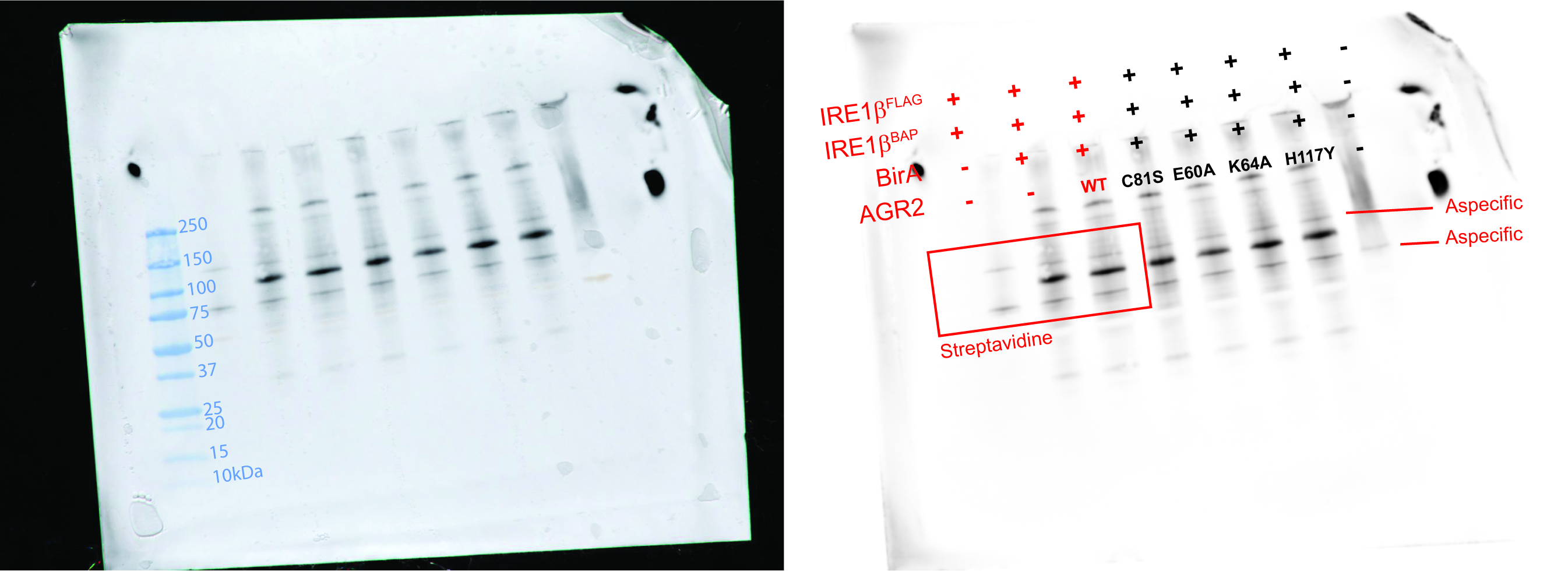

Supplement: Supplementary file 6 — Source Data Fig. 4 [file 44318_2023_15_MOESM6_ESM.zip › Figure 4/4E/western streptavidine - input samples.tif]

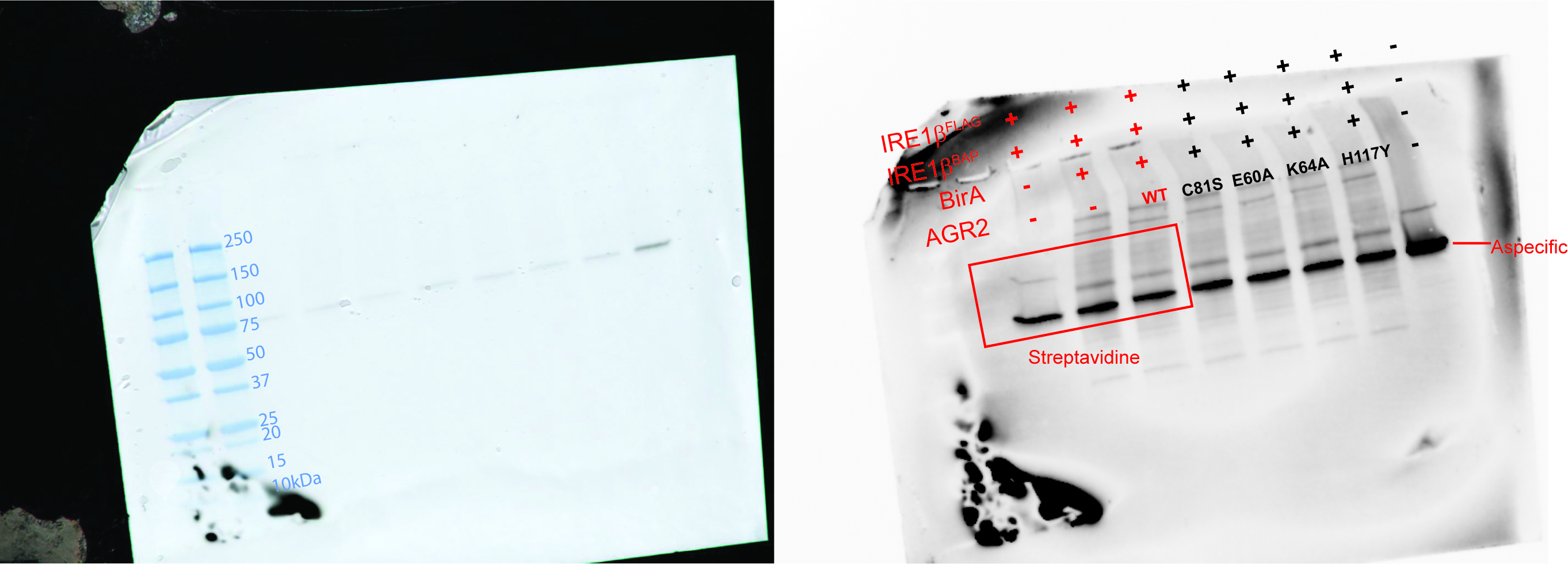

Supplement: Supplementary file 6 — Source Data Fig. 4 [file 44318_2023_15_MOESM6_ESM.zip › Figure 4/4E/western streptavidine - IP samples.tif]

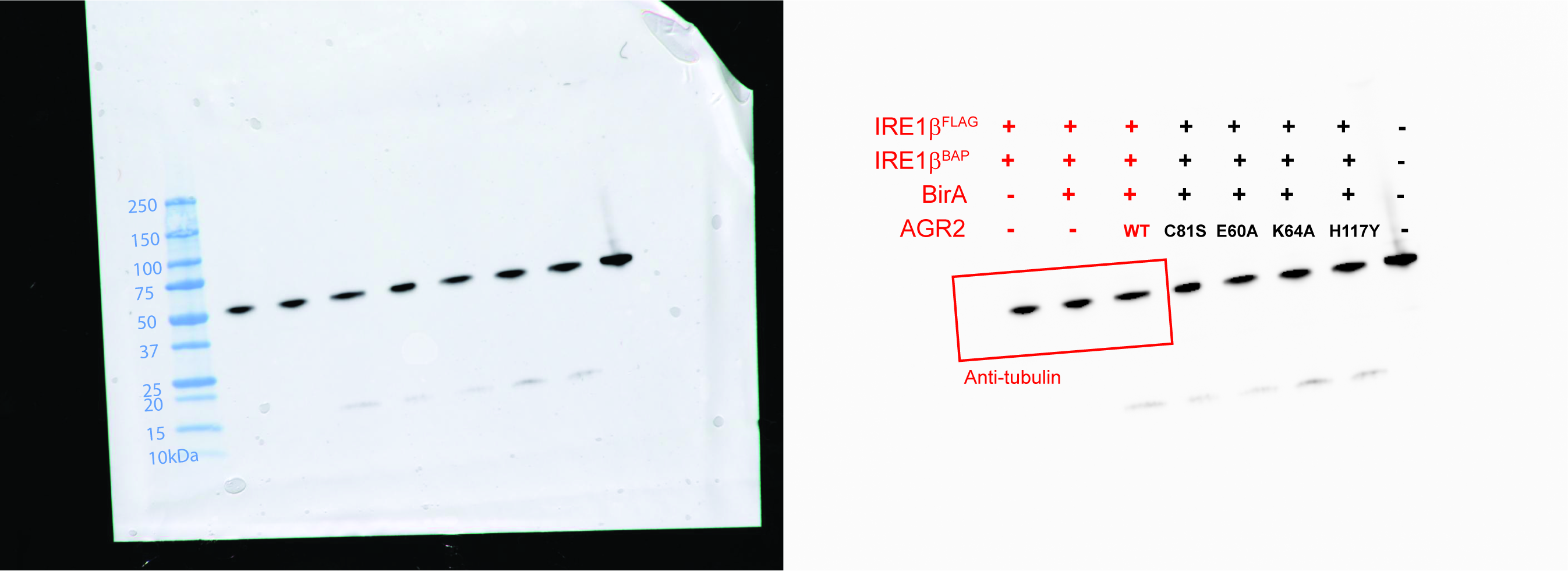

Supplement: Supplementary file 6 — Source Data Fig. 4 [file 44318_2023_15_MOESM6_ESM.zip › Figure 4/4E/western tubuline - input samples.tif]

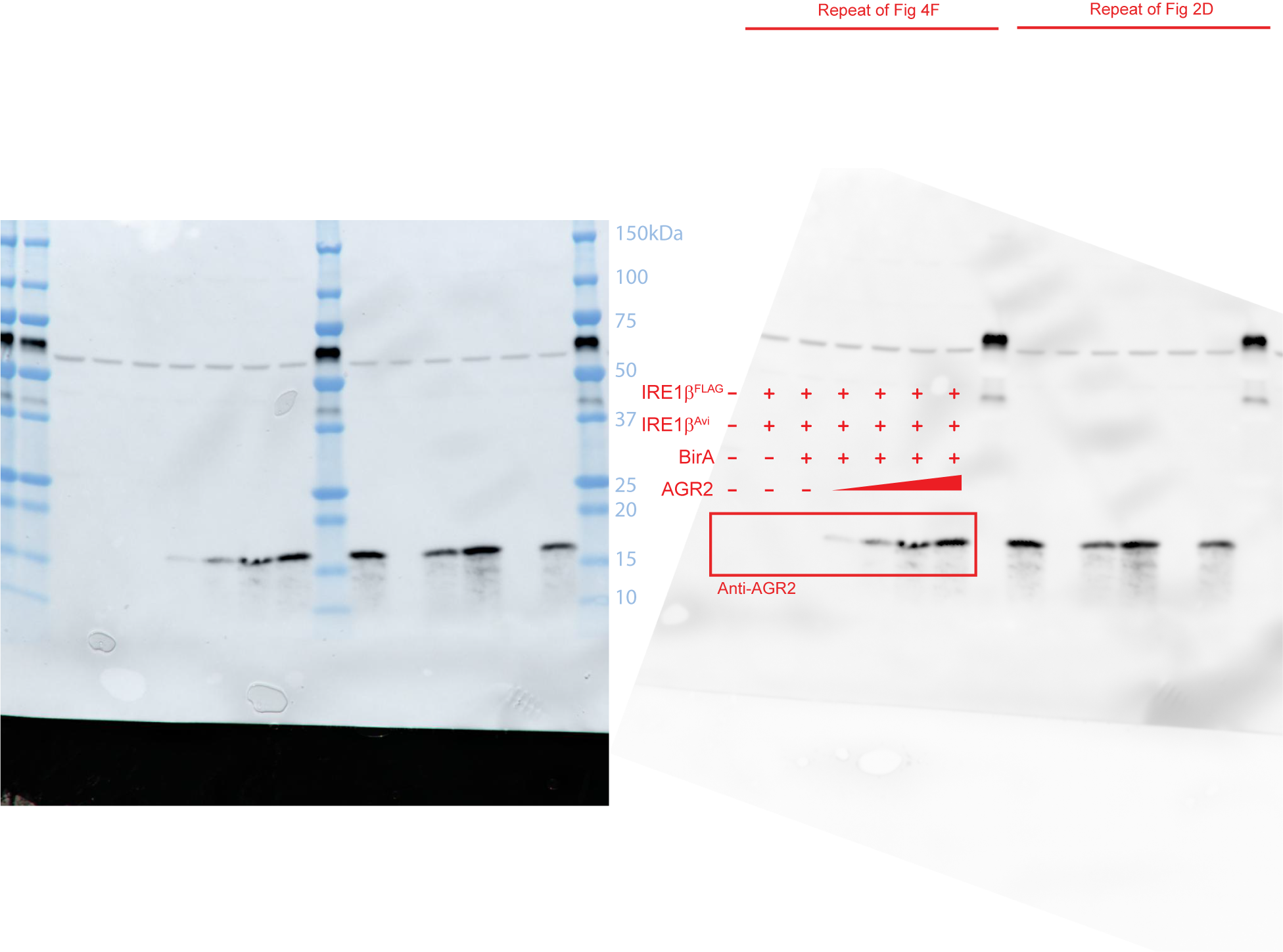

Supplement: Supplementary file 6 — Source Data Fig. 4 [file 44318_2023_15_MOESM6_ESM.zip › Figure 4/4F/Replicate/western AGR2 - input samples.tif]

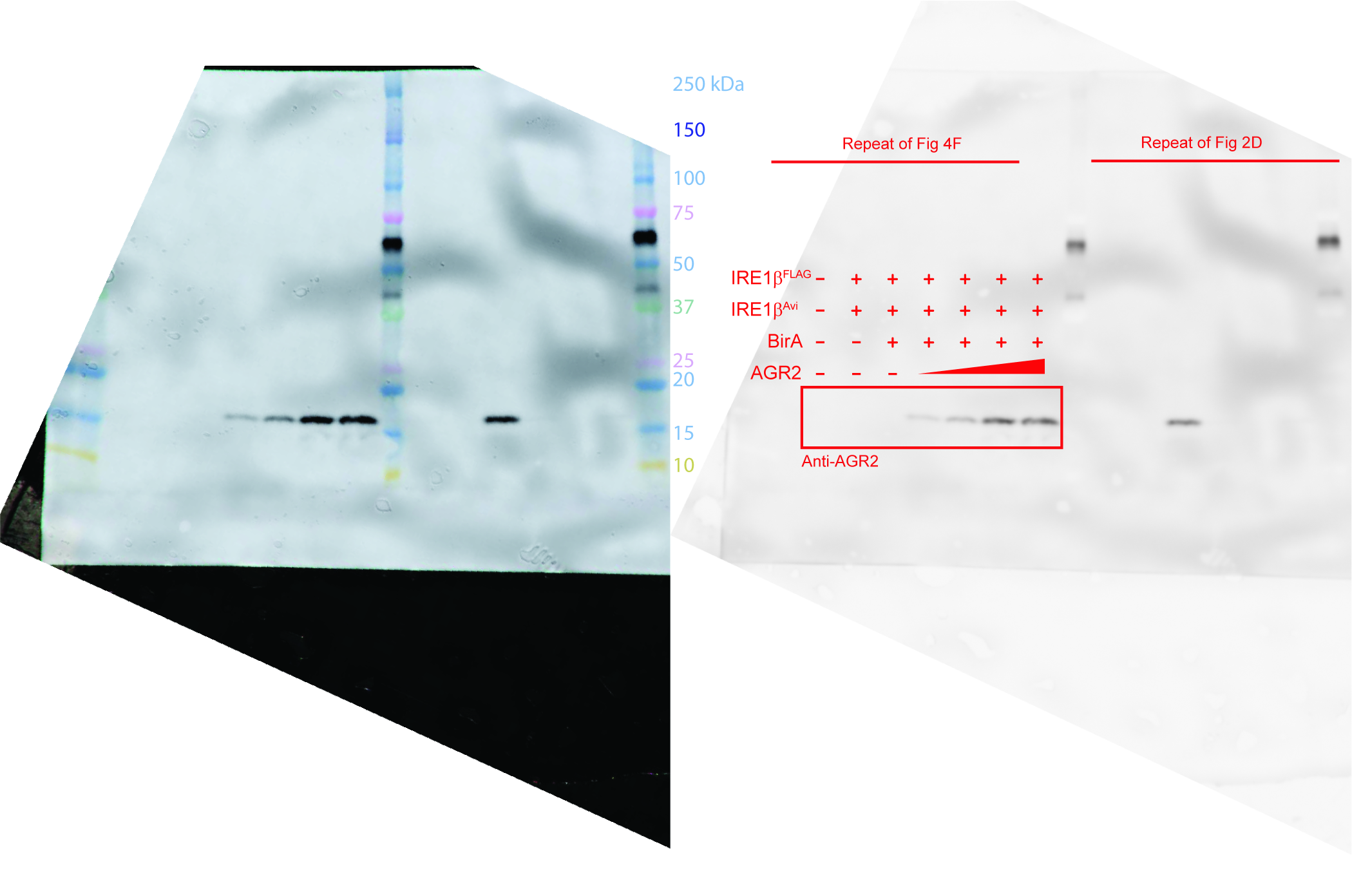

Supplement: Supplementary file 6 — Source Data Fig. 4 [file 44318_2023_15_MOESM6_ESM.zip › Figure 4/4F/Replicate/western AGR2 - IP samples.tif]

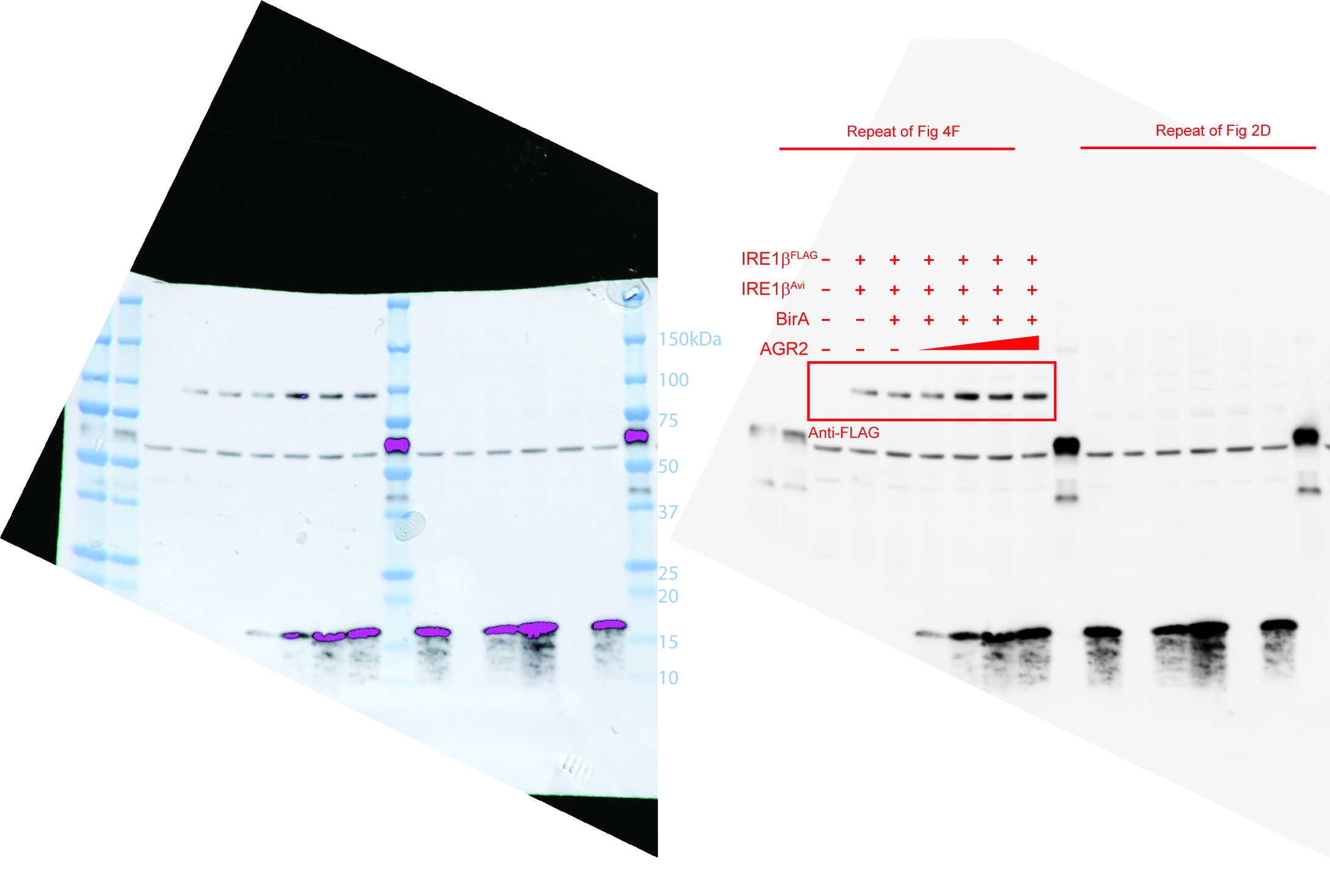

Supplement: Supplementary file 6 — Source Data Fig. 4 [file 44318_2023_15_MOESM6_ESM.zip › Figure 4/4F/Replicate/western FLAG - input samples.tif]

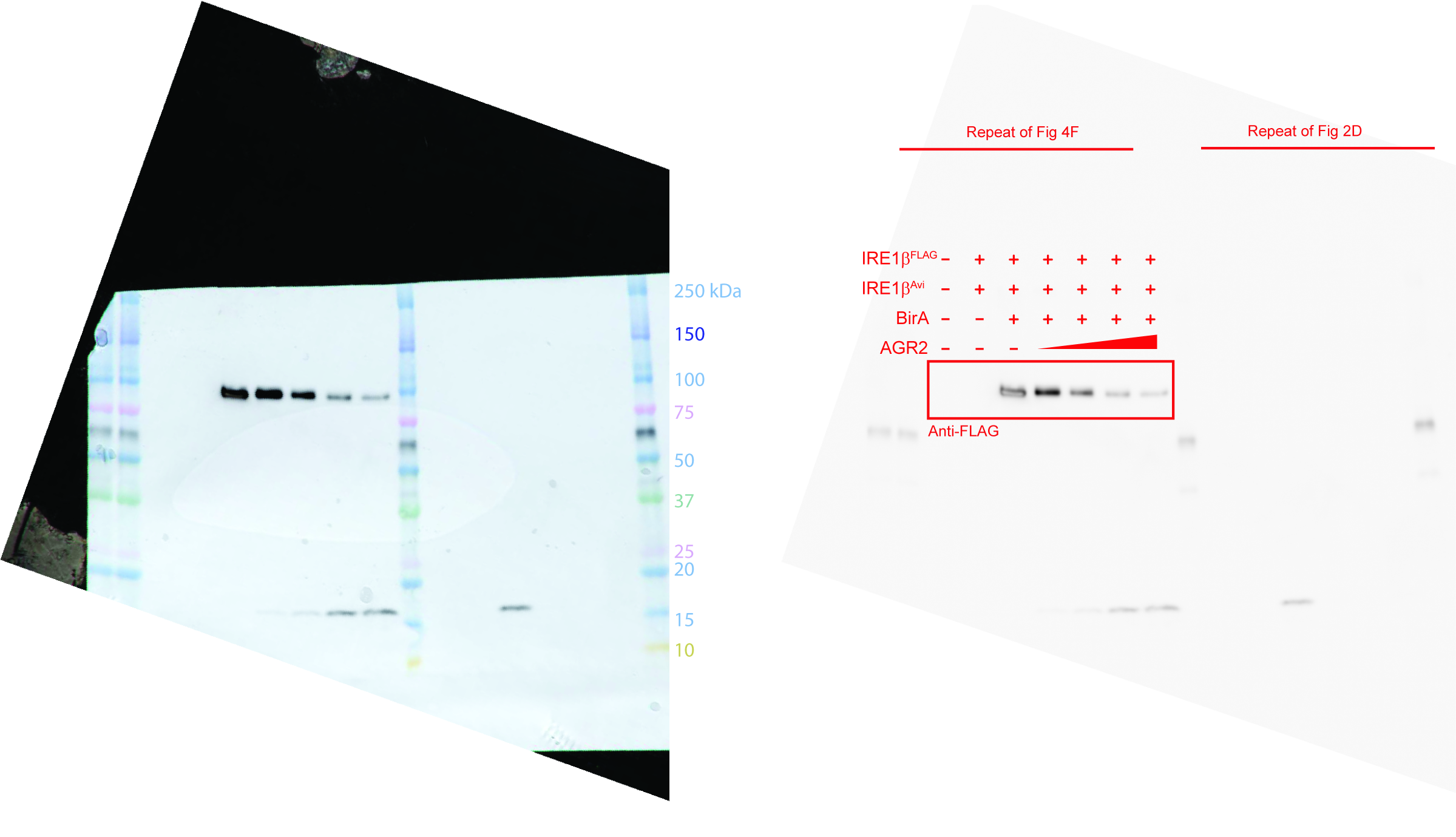

Supplement: Supplementary file 6 — Source Data Fig. 4 [file 44318_2023_15_MOESM6_ESM.zip › Figure 4/4F/Replicate/western FLAG - IP samples.tif]

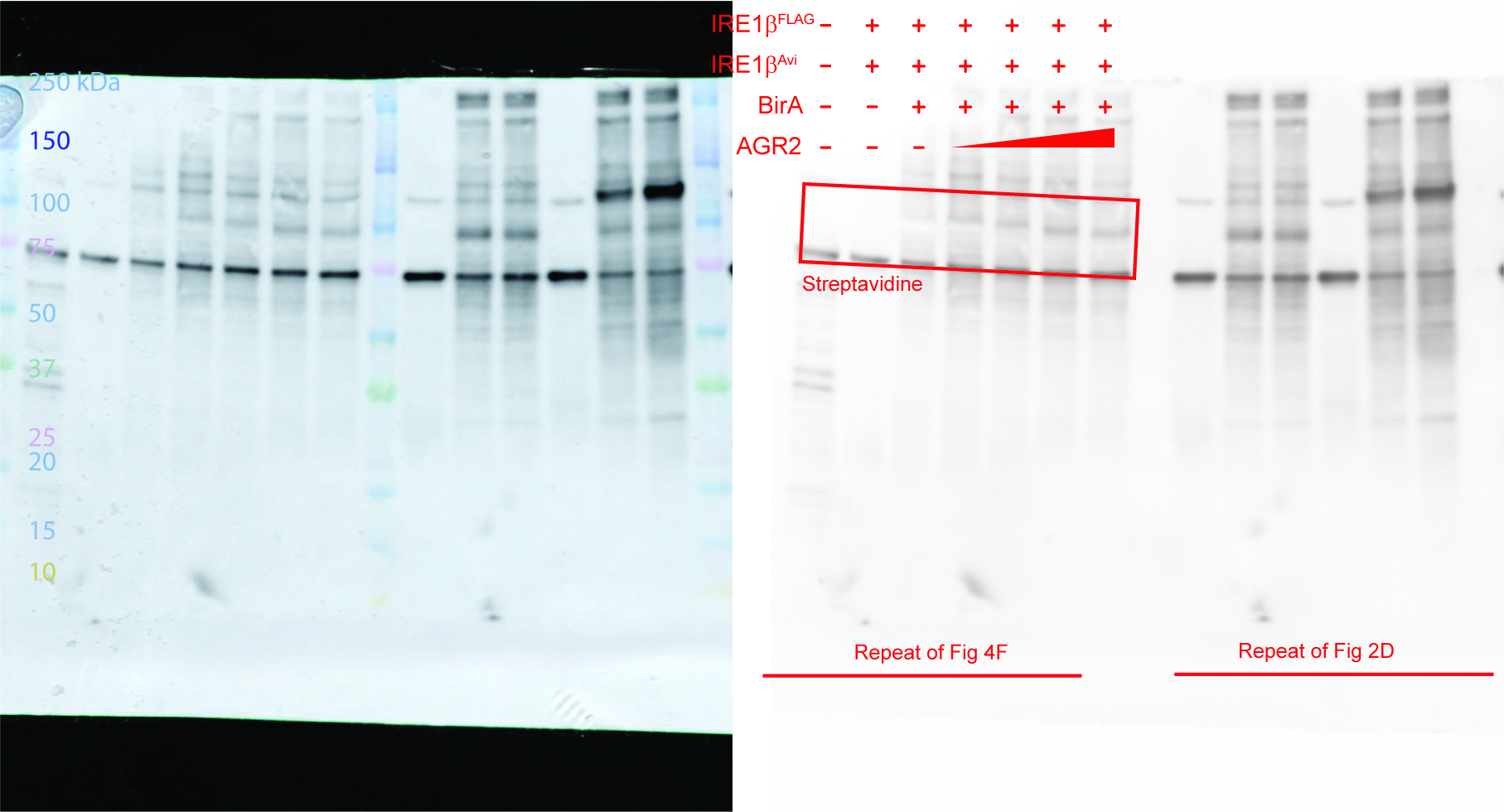

Supplement: Supplementary file 6 — Source Data Fig. 4 [file 44318_2023_15_MOESM6_ESM.zip › Figure 4/4F/Replicate/western streptavidine - IP samples.tif]

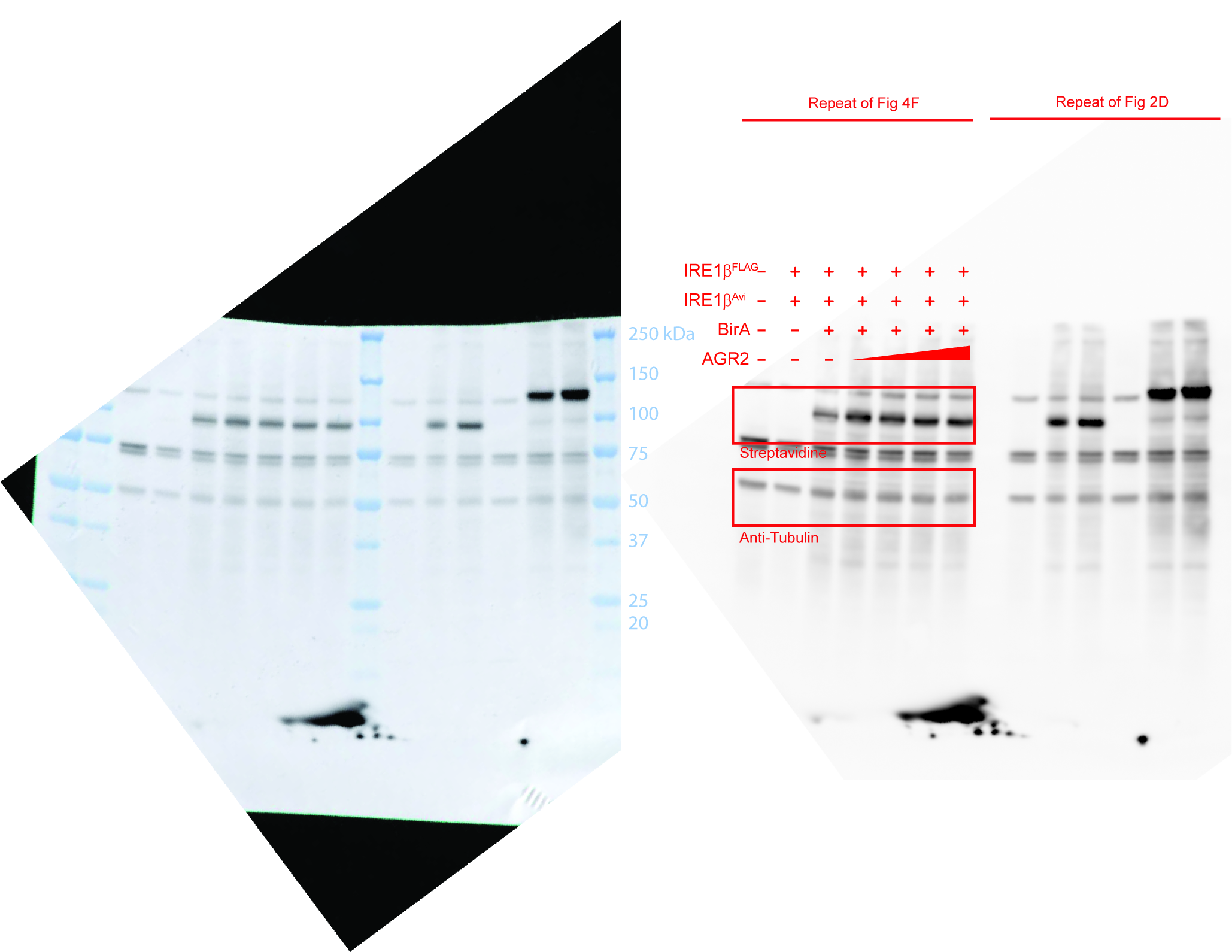

Supplement: Supplementary file 6 — Source Data Fig. 4 [file 44318_2023_15_MOESM6_ESM.zip › Figure 4/4F/Replicate/western streptavidine and tubulin - input samples.tif]

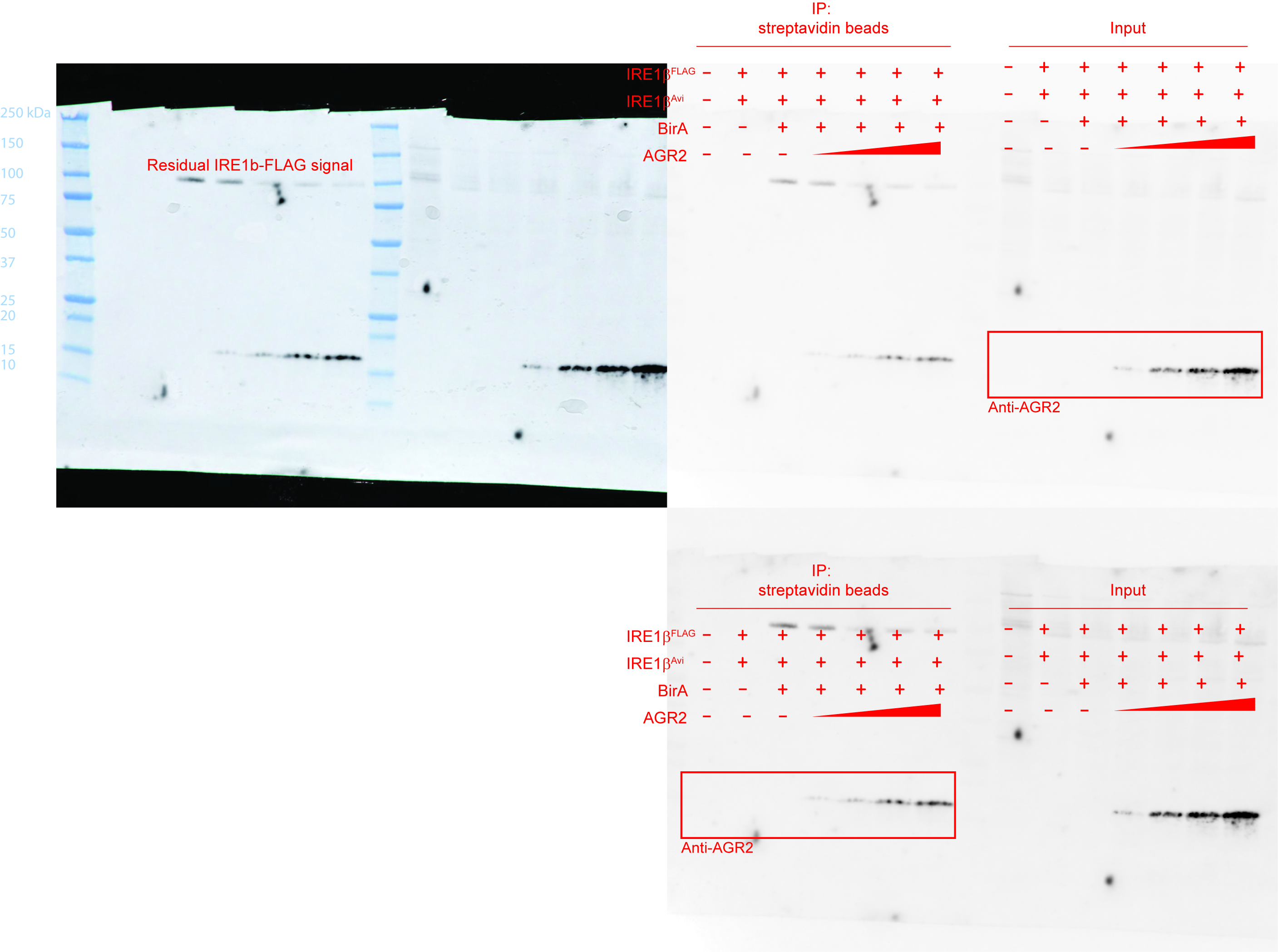

Supplement: Supplementary file 6 — Source Data Fig. 4 [file 44318_2023_15_MOESM6_ESM.zip › Figure 4/4F/western agr2.tif]

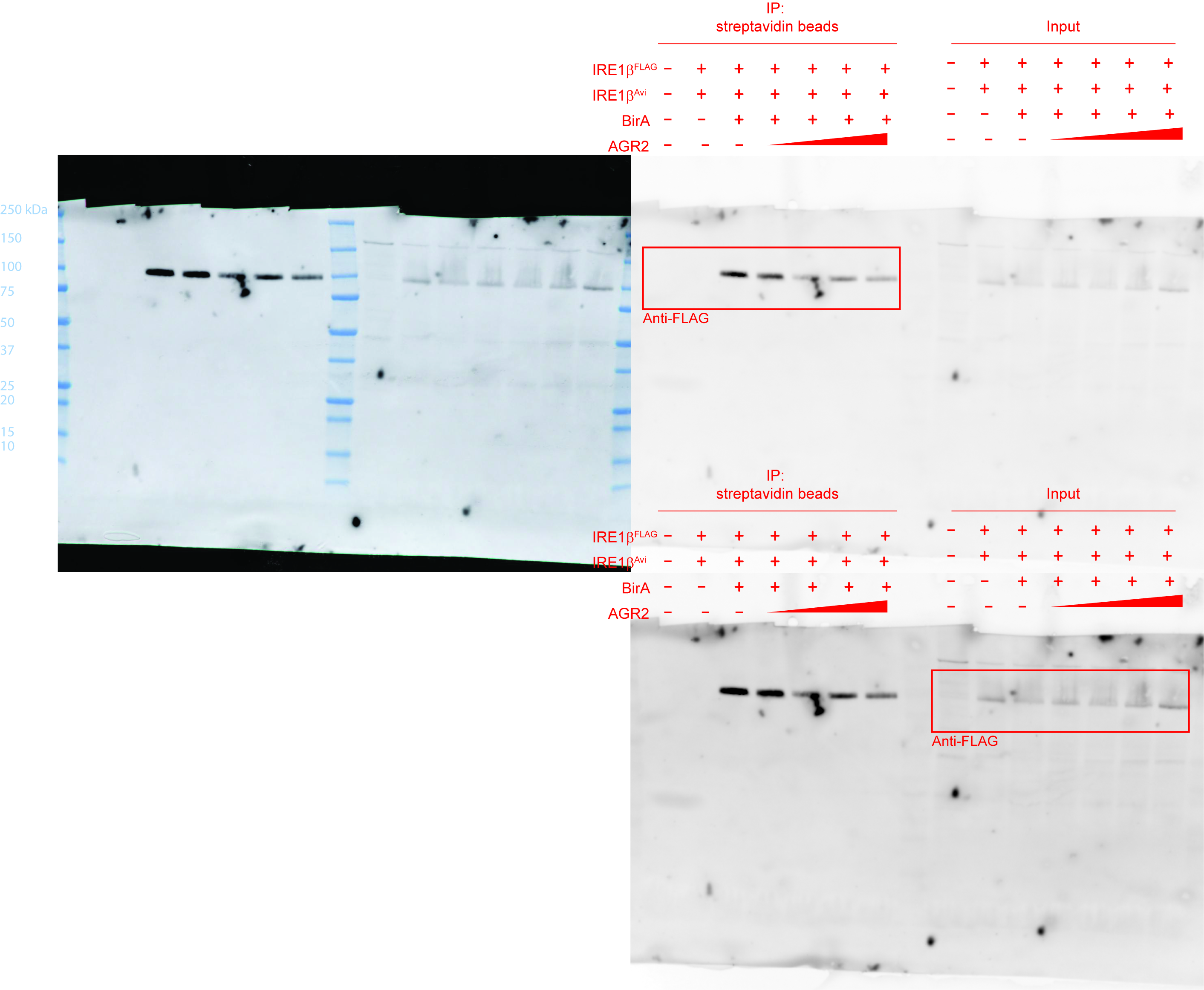

Supplement: Supplementary file 6 — Source Data Fig. 4 [file 44318_2023_15_MOESM6_ESM.zip › Figure 4/4F/western FLAG.tif]

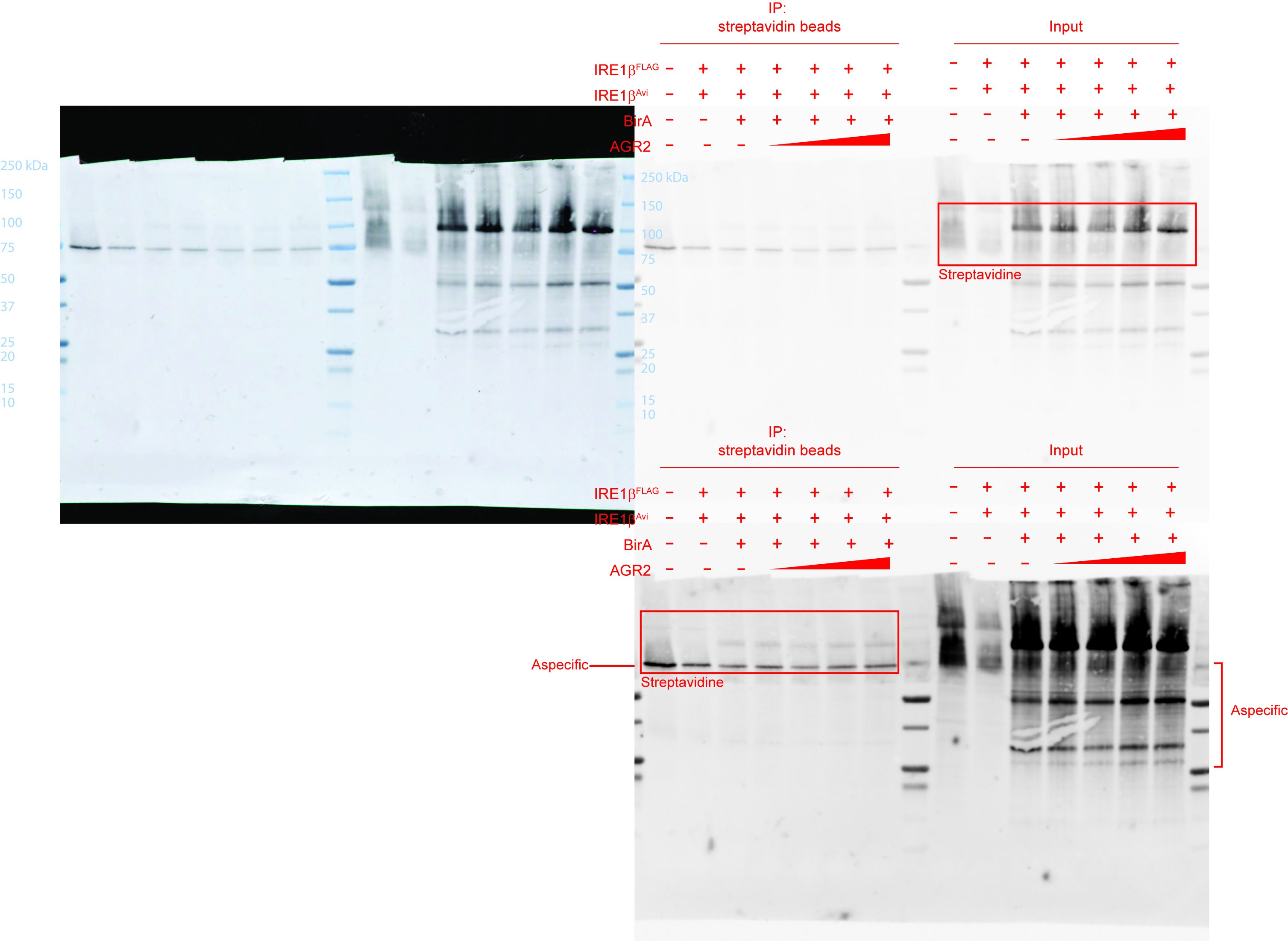

Supplement: Supplementary file 6 — Source Data Fig. 4 [file 44318_2023_15_MOESM6_ESM.zip › Figure 4/4F/western streptavidin.tif]

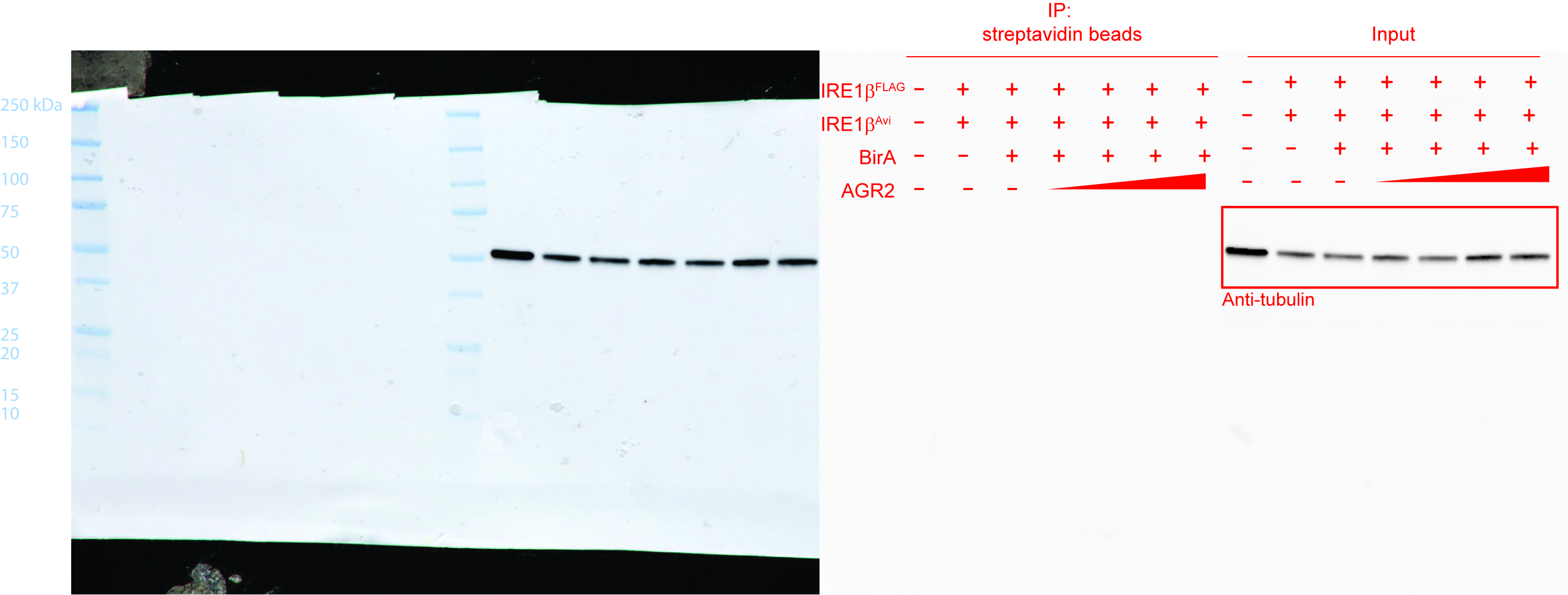

Supplement: Supplementary file 6 — Source Data Fig. 4 [file 44318_2023_15_MOESM6_ESM.zip › Figure 4/4F/western tubulin.tif]

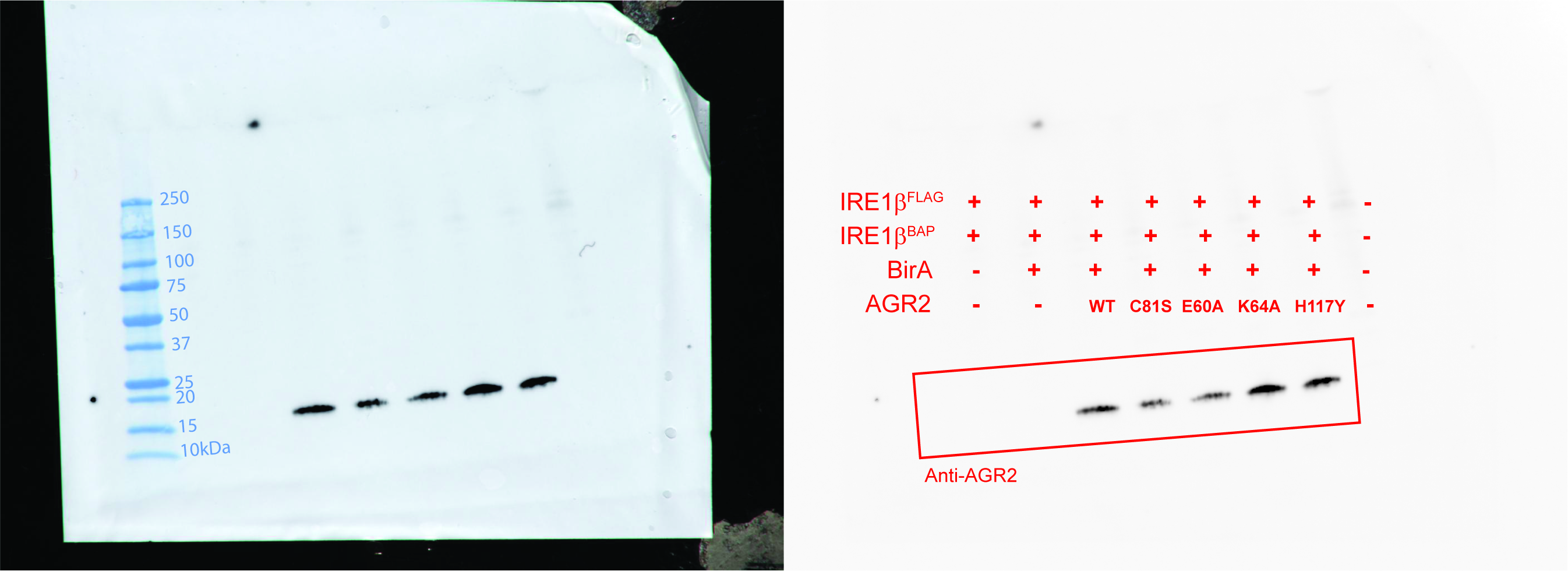

Supplement: Supplementary file 7 — Source Data Fig. 5 [file 44318_2023_15_MOESM7_ESM.zip › Figure 5/5C/Replicates/western AGR2 - input samples repl1.tif]

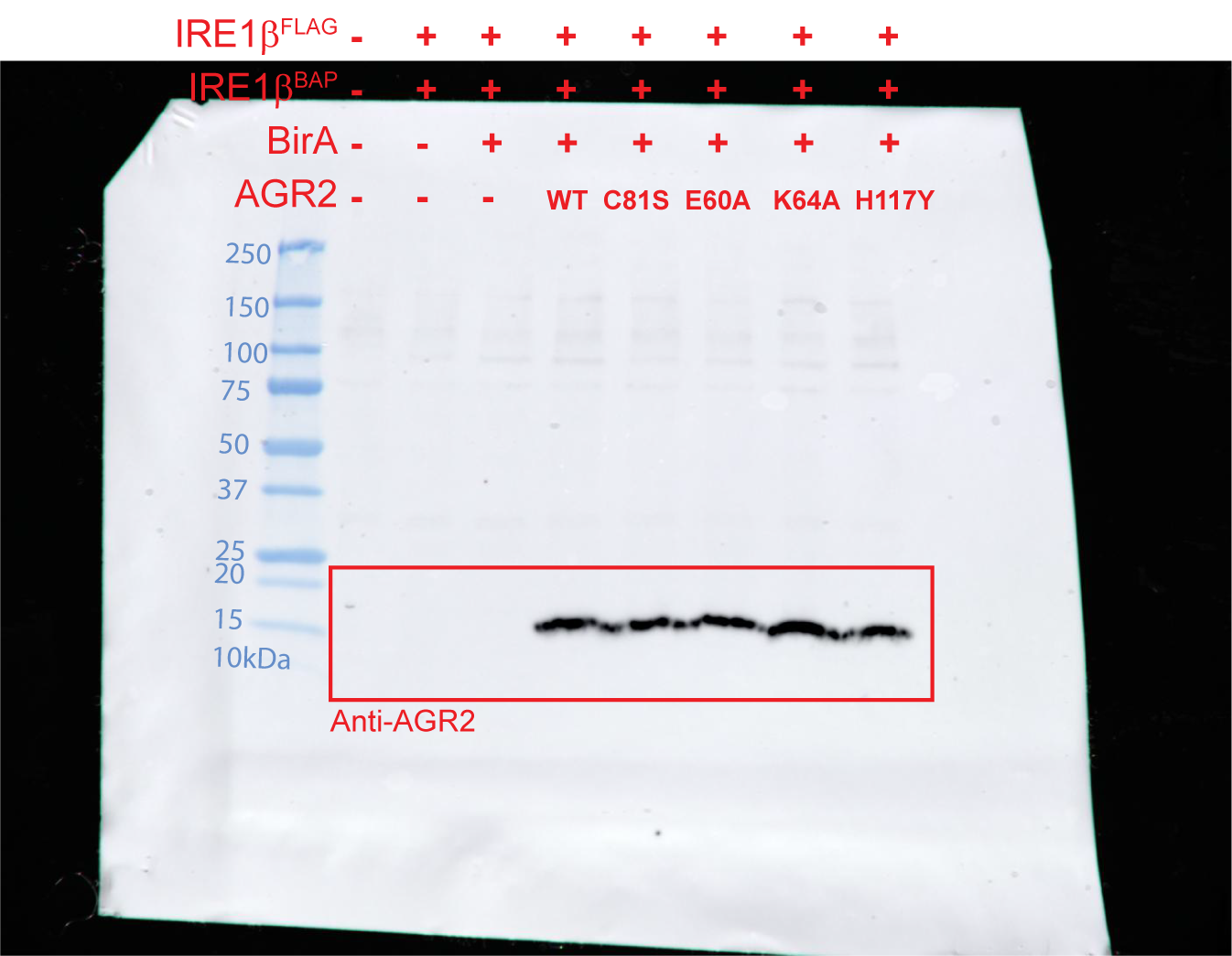

Supplement: Supplementary file 7 — Source Data Fig. 5 [file 44318_2023_15_MOESM7_ESM.zip › Figure 5/5C/Replicates/western AGR2 - input samples repl2.tif]

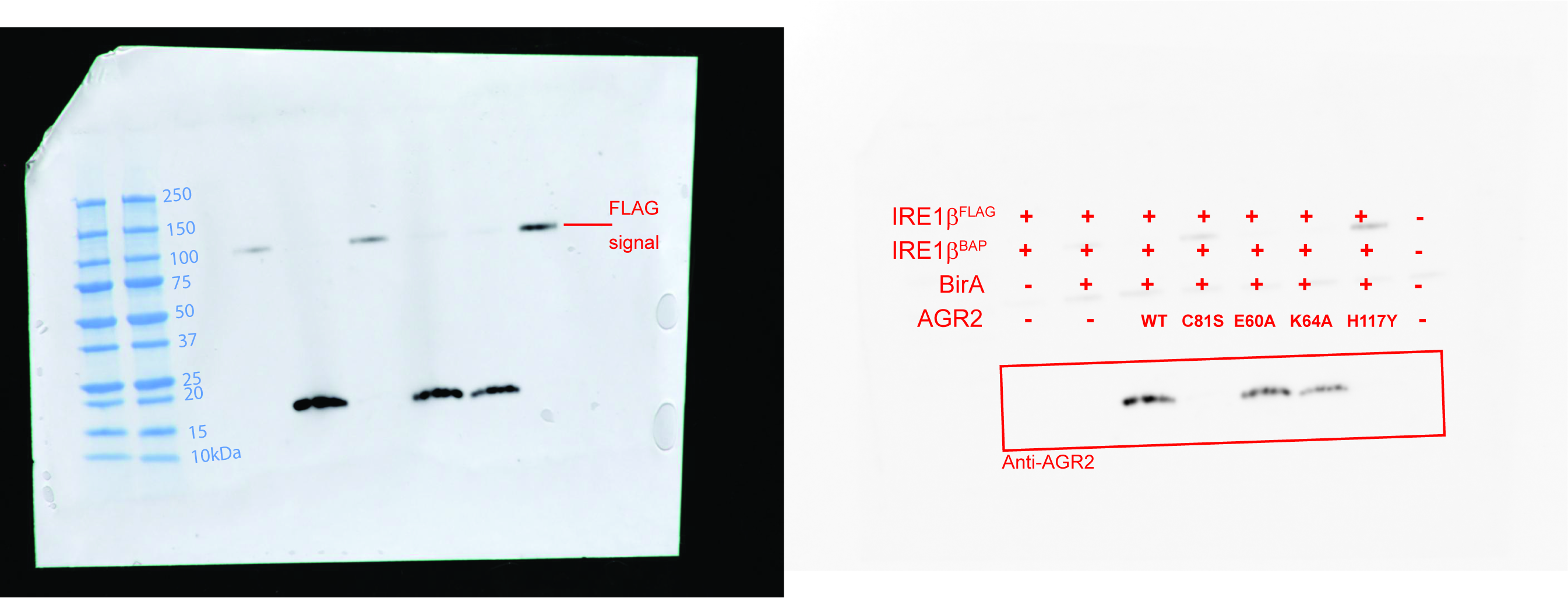

Supplement: Supplementary file 7 — Source Data Fig. 5 [file 44318_2023_15_MOESM7_ESM.zip › Figure 5/5C/Replicates/western AGR2 - IP samples repl1.tif]

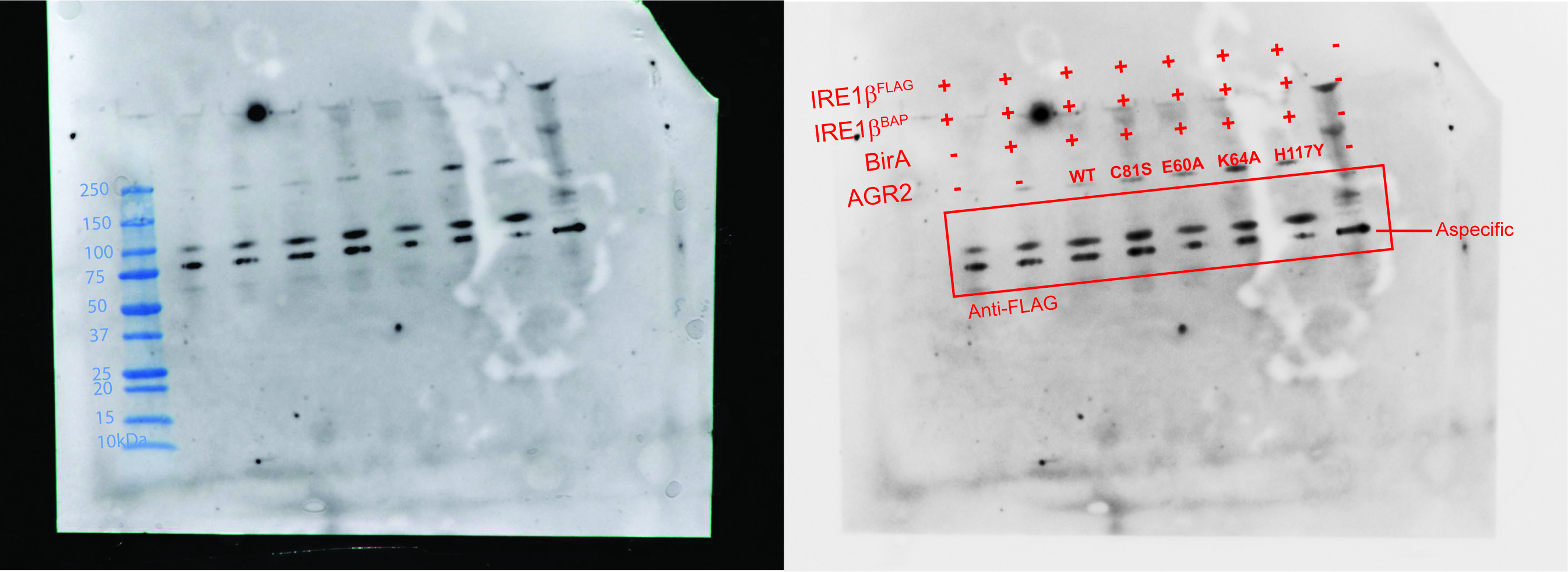

Supplement: Supplementary file 7 — Source Data Fig. 5 [file 44318_2023_15_MOESM7_ESM.zip › Figure 5/5C/Replicates/western FLAG - input samples repl1.tif]

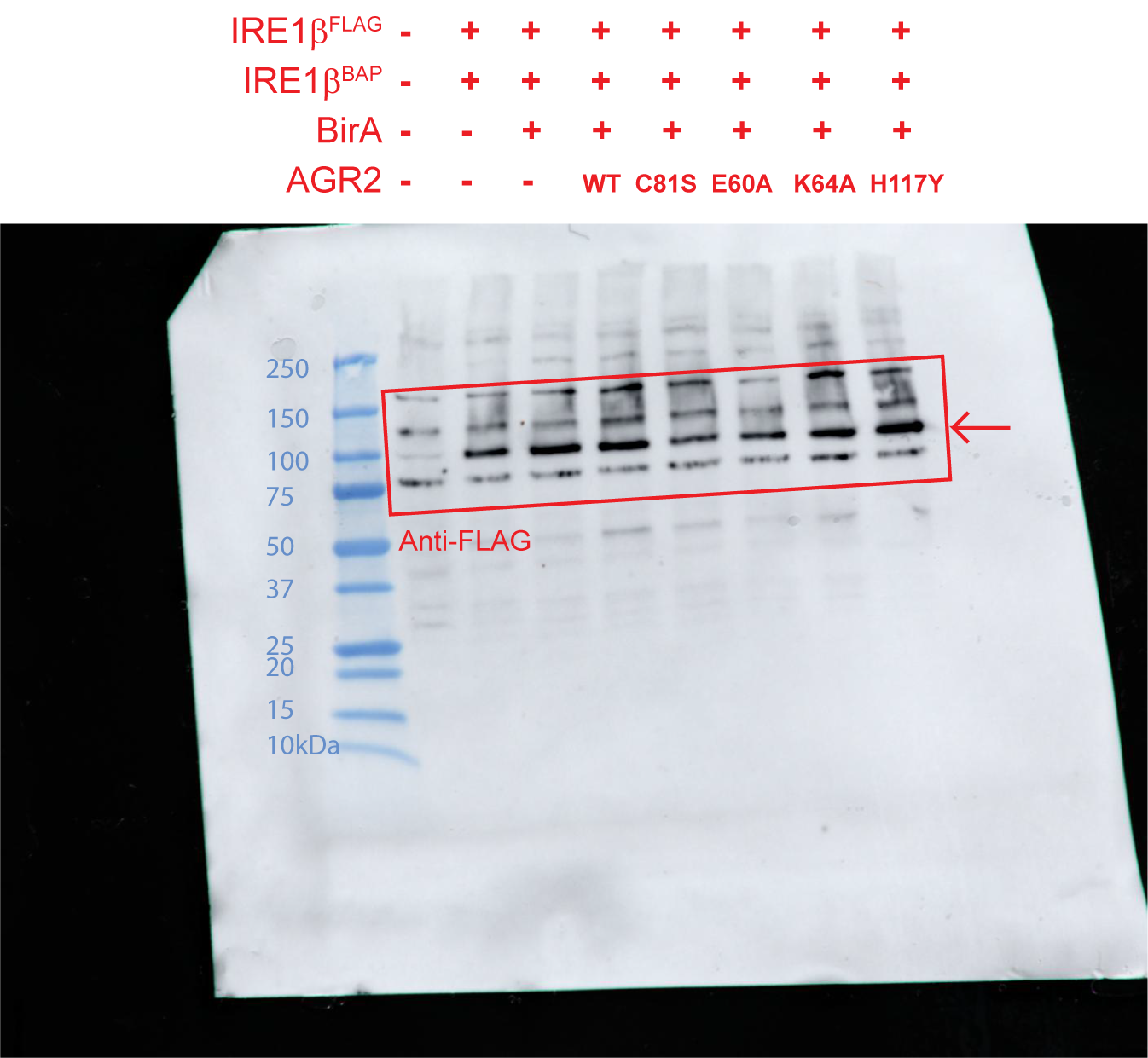

Supplement: Supplementary file 7 — Source Data Fig. 5 [file 44318_2023_15_MOESM7_ESM.zip › Figure 5/5C/Replicates/western FLAG - input samples repl2.tif]

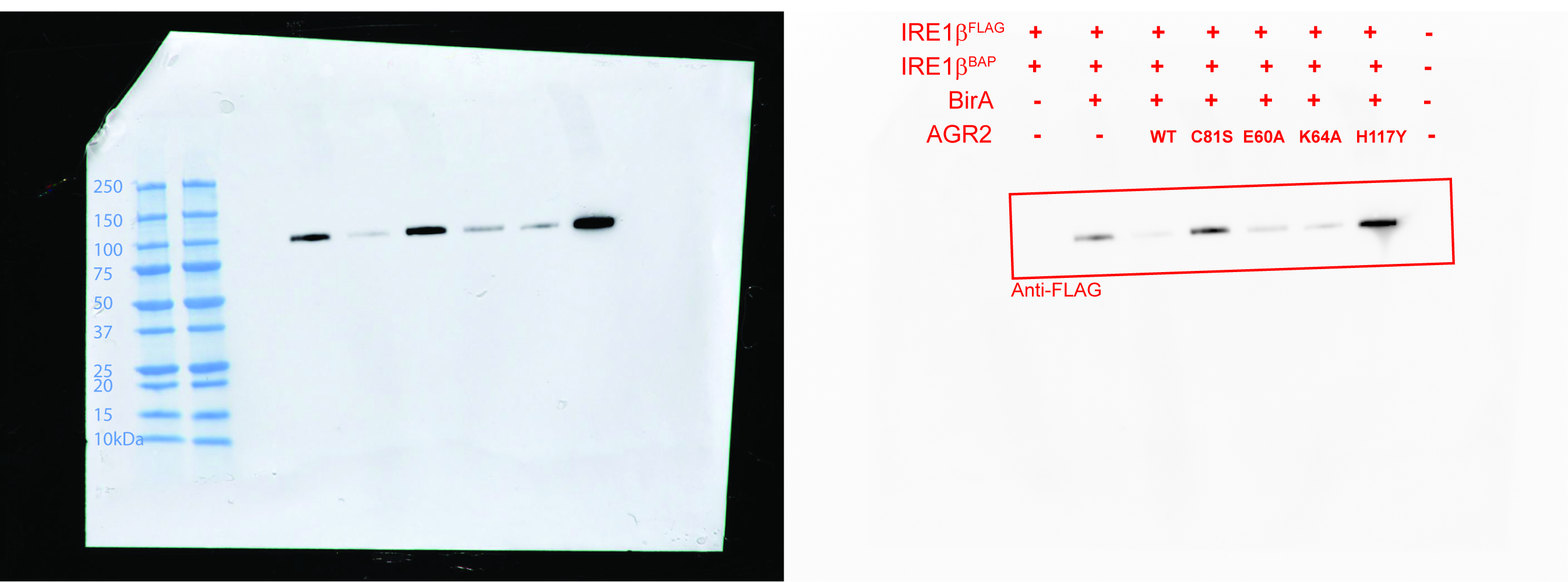

Supplement: Supplementary file 7 — Source Data Fig. 5 [file 44318_2023_15_MOESM7_ESM.zip › Figure 5/5C/Replicates/western FLAG - IP samples repl1.tif]

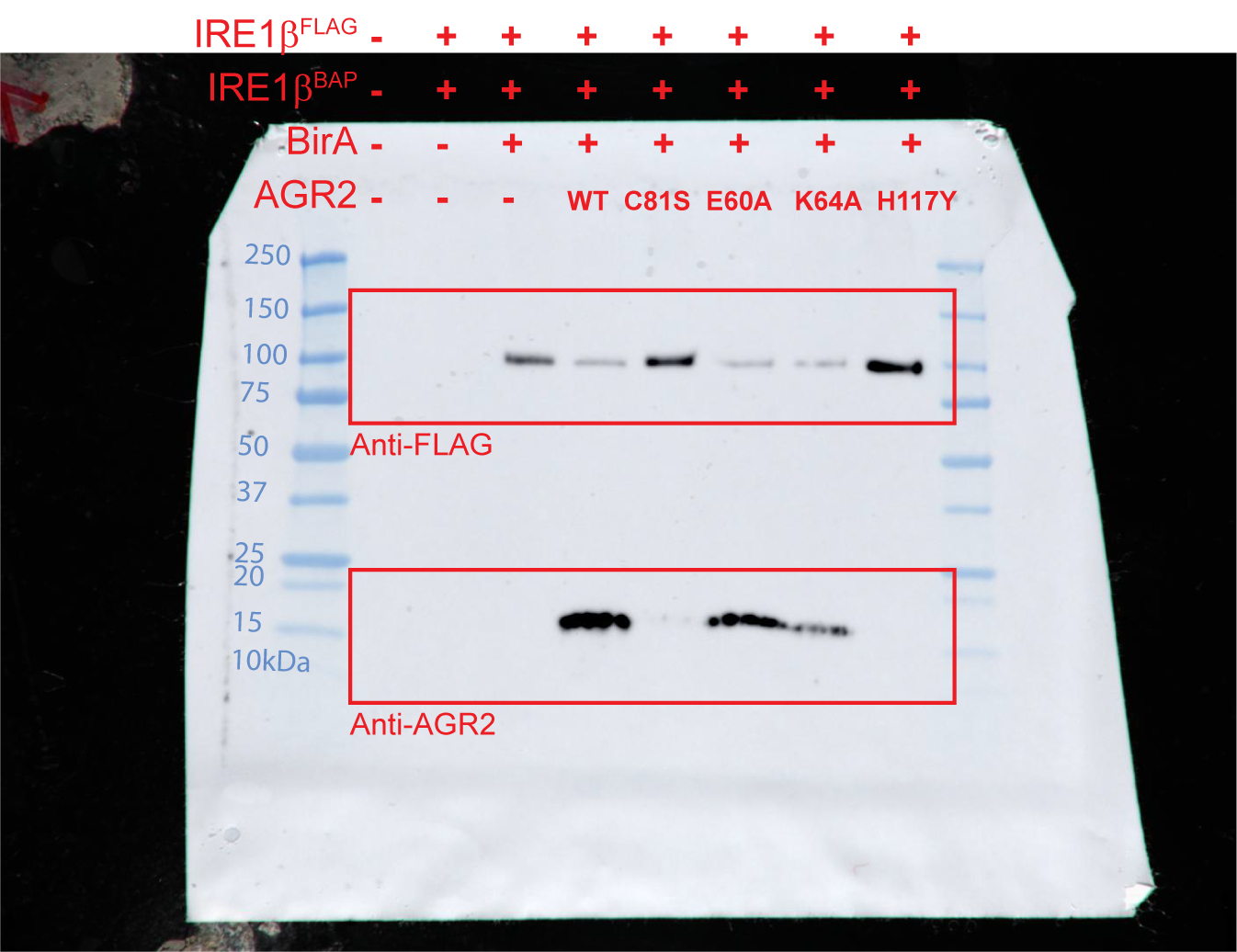

Supplement: Supplementary file 7 — Source Data Fig. 5 [file 44318_2023_15_MOESM7_ESM.zip › Figure 5/5C/Replicates/western FLAG and AGR2 - IP samples repl2.tif]

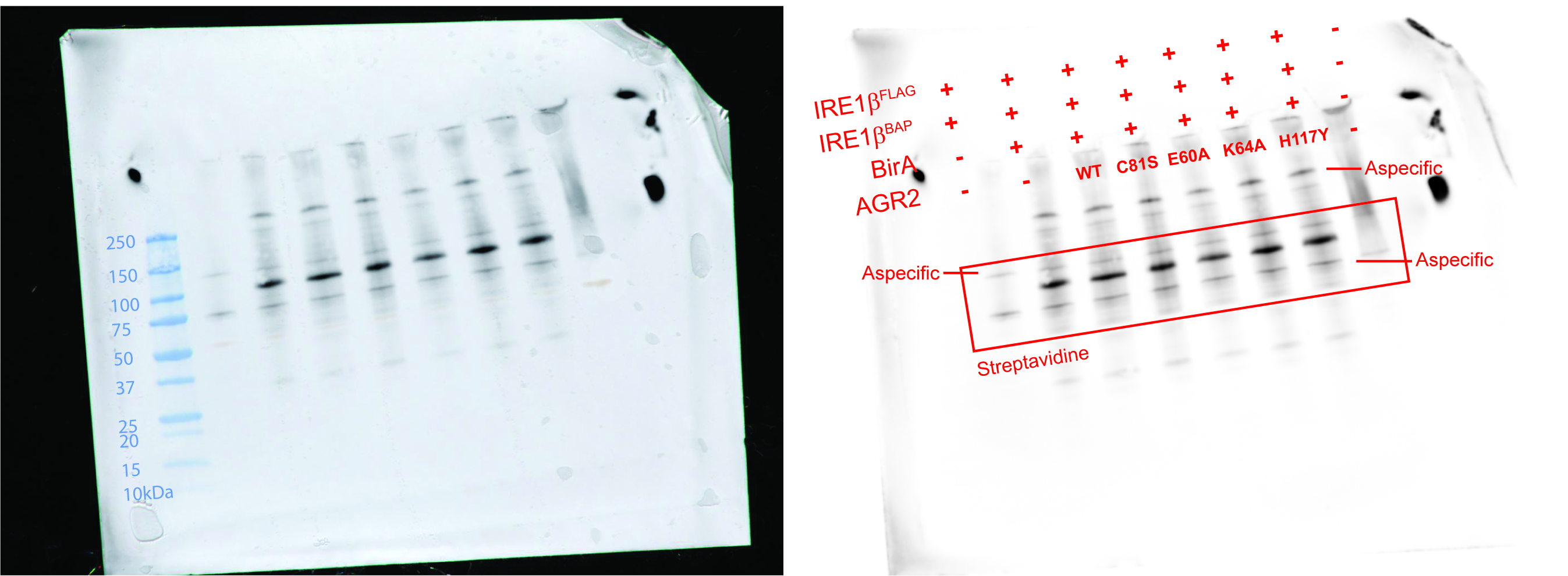

Supplement: Supplementary file 7 — Source Data Fig. 5 [file 44318_2023_15_MOESM7_ESM.zip › Figure 5/5C/Replicates/western streptavidin - input samples repl1.tif]

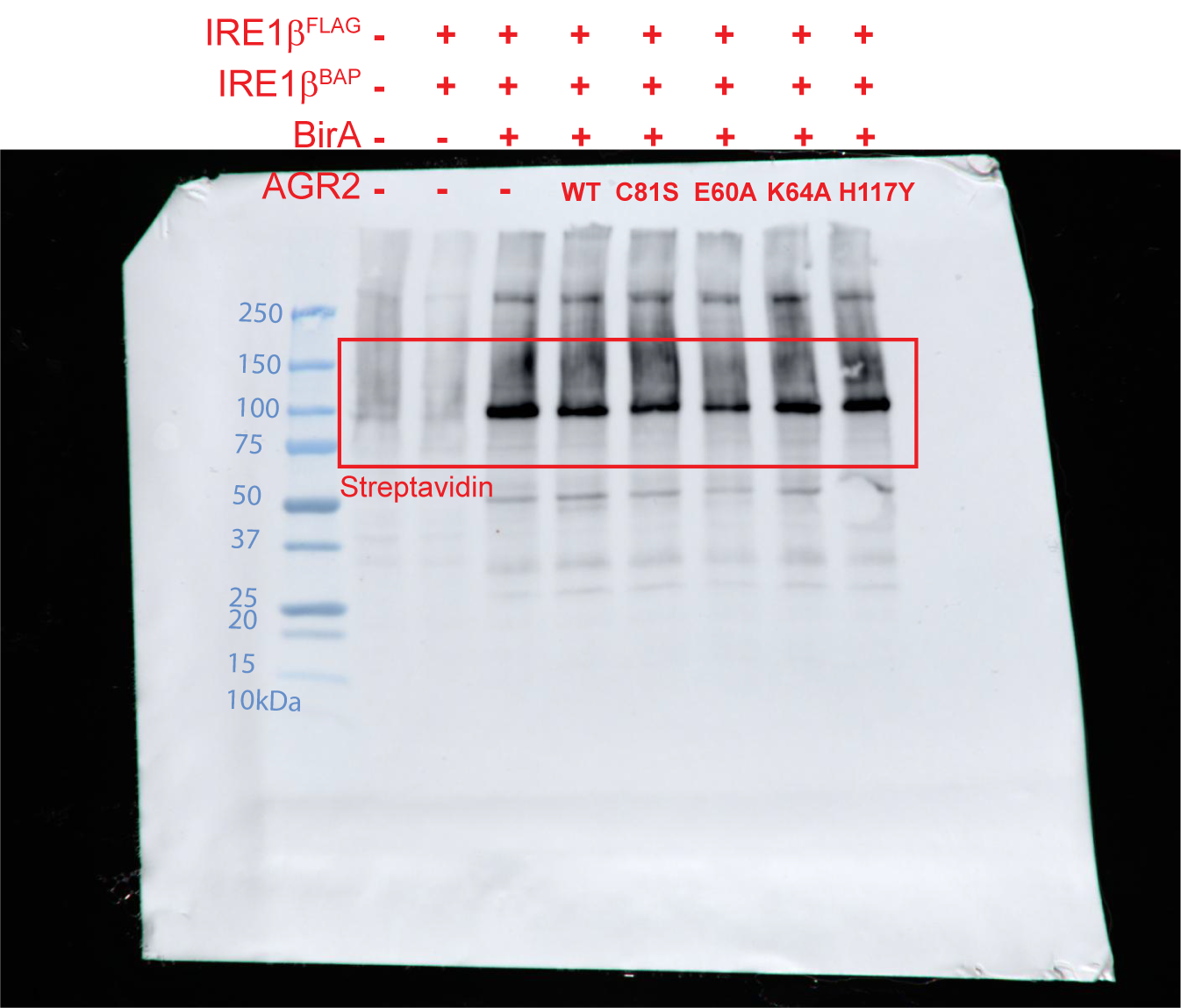

Supplement: Supplementary file 7 — Source Data Fig. 5 [file 44318_2023_15_MOESM7_ESM.zip › Figure 5/5C/Replicates/western streptavidin - input samples repl2.tif]

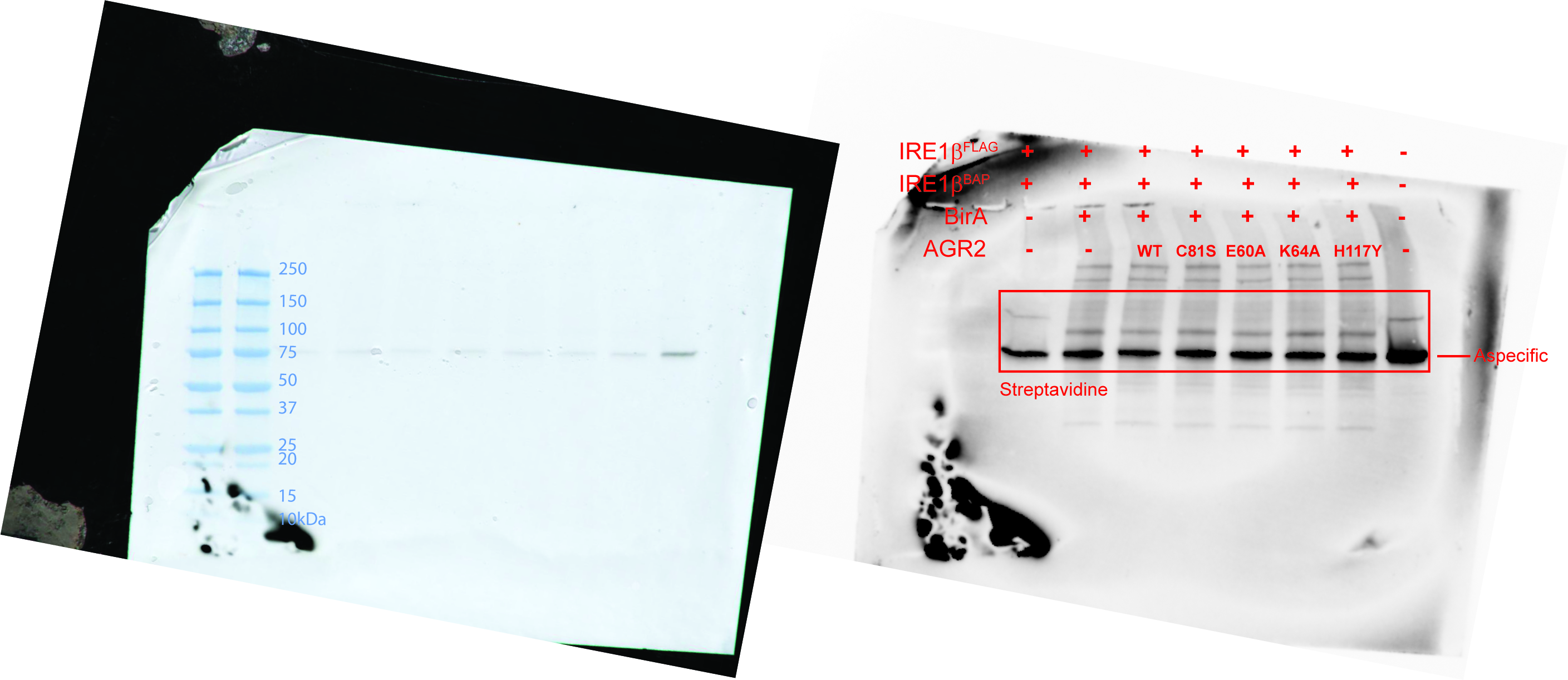

Supplement: Supplementary file 7 — Source Data Fig. 5 [file 44318_2023_15_MOESM7_ESM.zip › Figure 5/5C/Replicates/western streptavidin - IP samples repl1.tif]

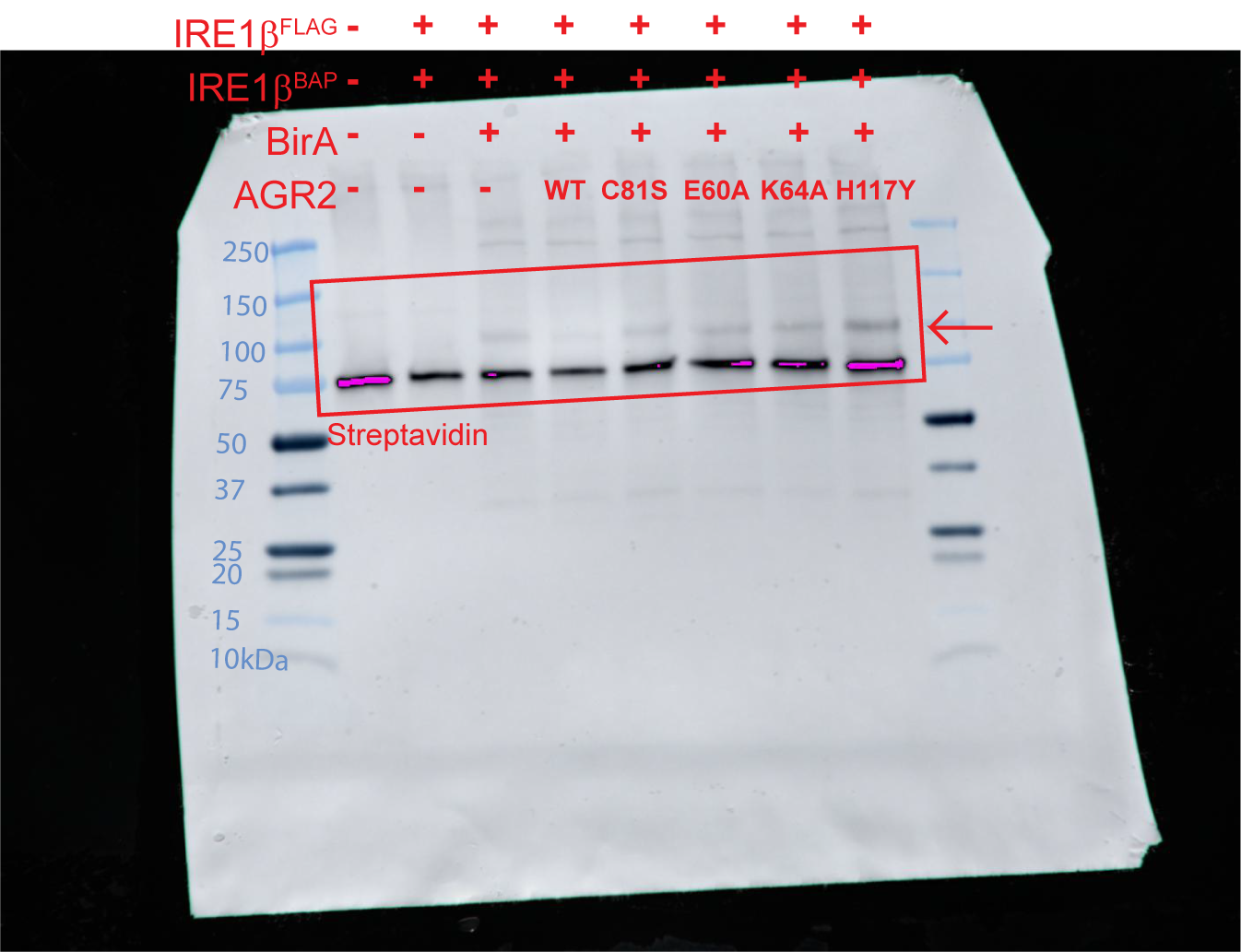

Supplement: Supplementary file 7 — Source Data Fig. 5 [file 44318_2023_15_MOESM7_ESM.zip › Figure 5/5C/Replicates/western streptavidin - IP samples repl2.tif]

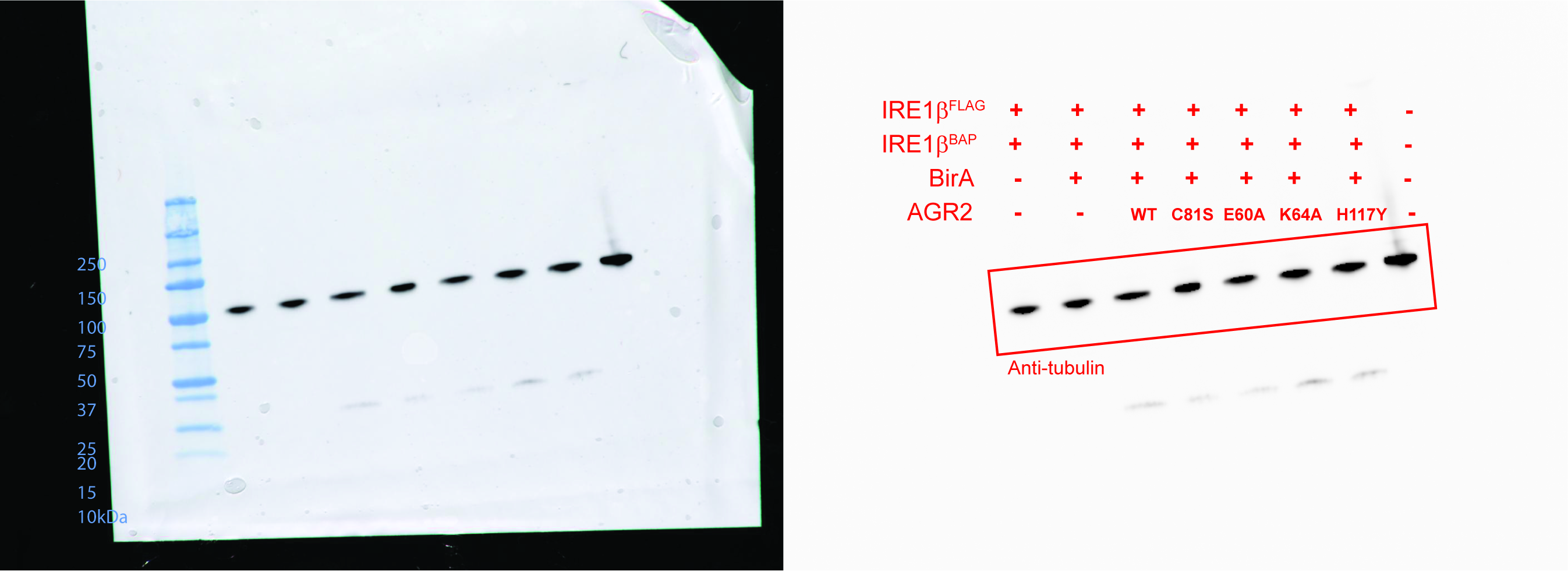

Supplement: Supplementary file 7 — Source Data Fig. 5 [file 44318_2023_15_MOESM7_ESM.zip › Figure 5/5C/Replicates/western tubulin - input samples repl1.tif]

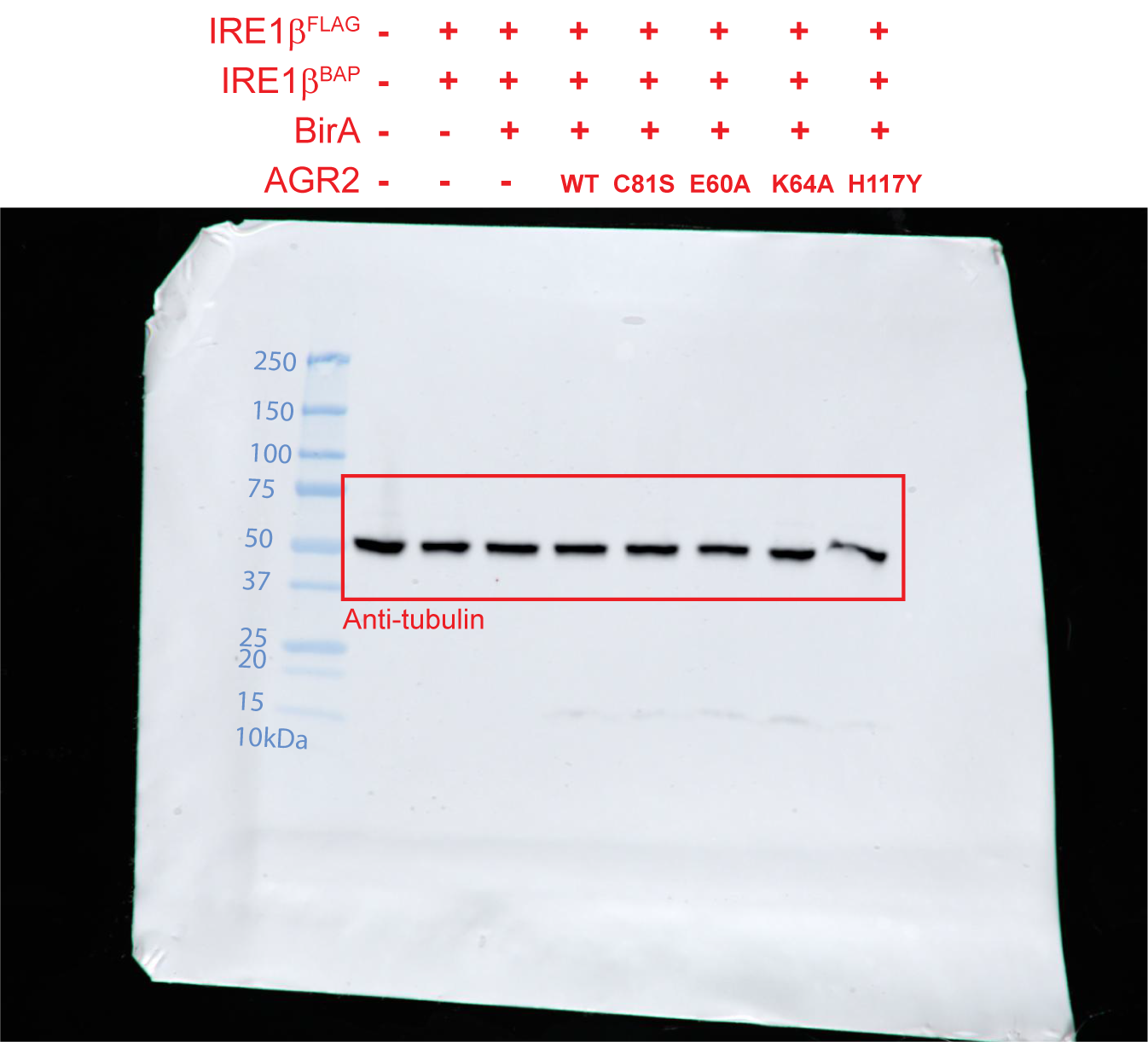

Supplement: Supplementary file 7 — Source Data Fig. 5 [file 44318_2023_15_MOESM7_ESM.zip › Figure 5/5C/Replicates/western tubulin - input samples repl2.tif]

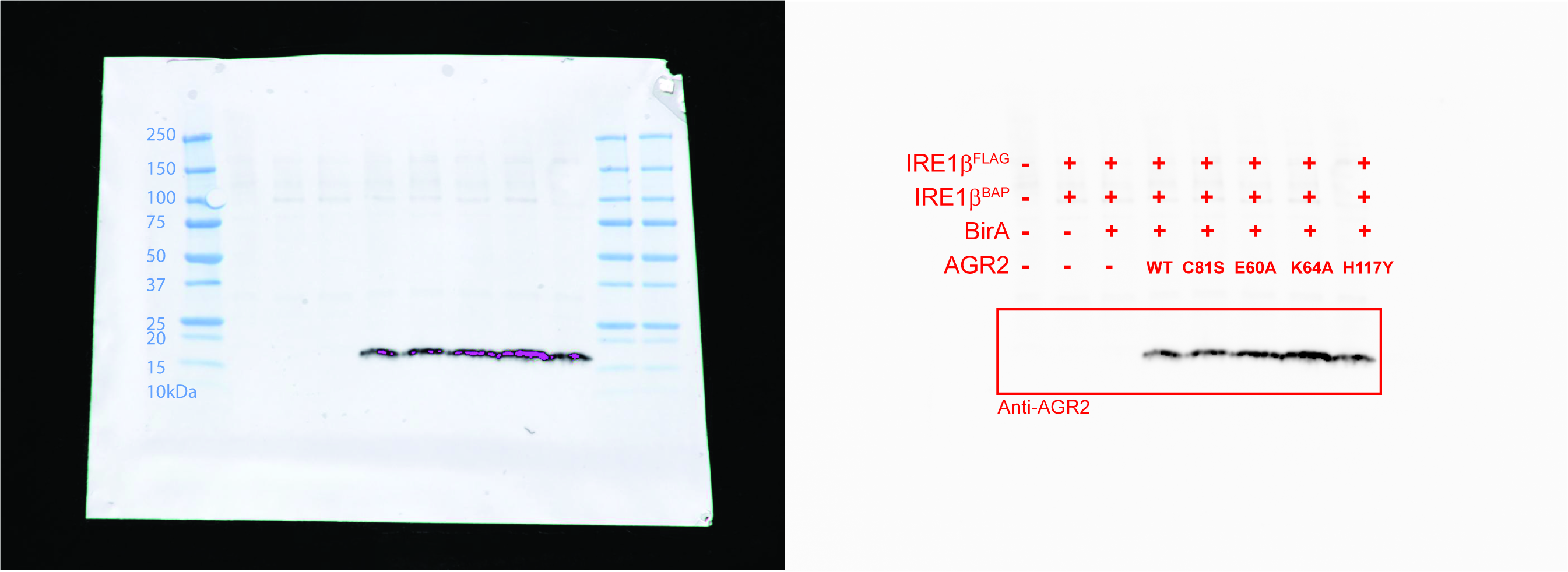

Supplement: Supplementary file 7 — Source Data Fig. 5 [file 44318_2023_15_MOESM7_ESM.zip › Figure 5/5C/western AGR2 - input samples .tif]

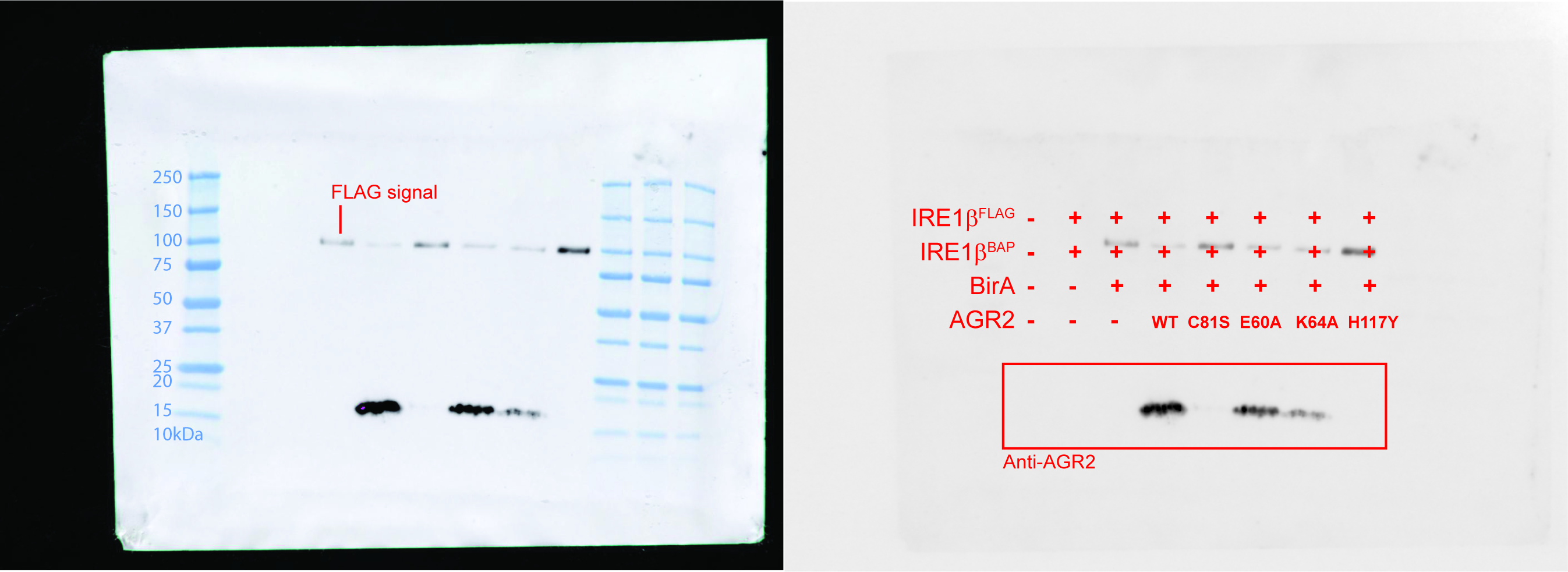

Supplement: Supplementary file 7 — Source Data Fig. 5 [file 44318_2023_15_MOESM7_ESM.zip › Figure 5/5C/western AGR2 - IP samples .tif]

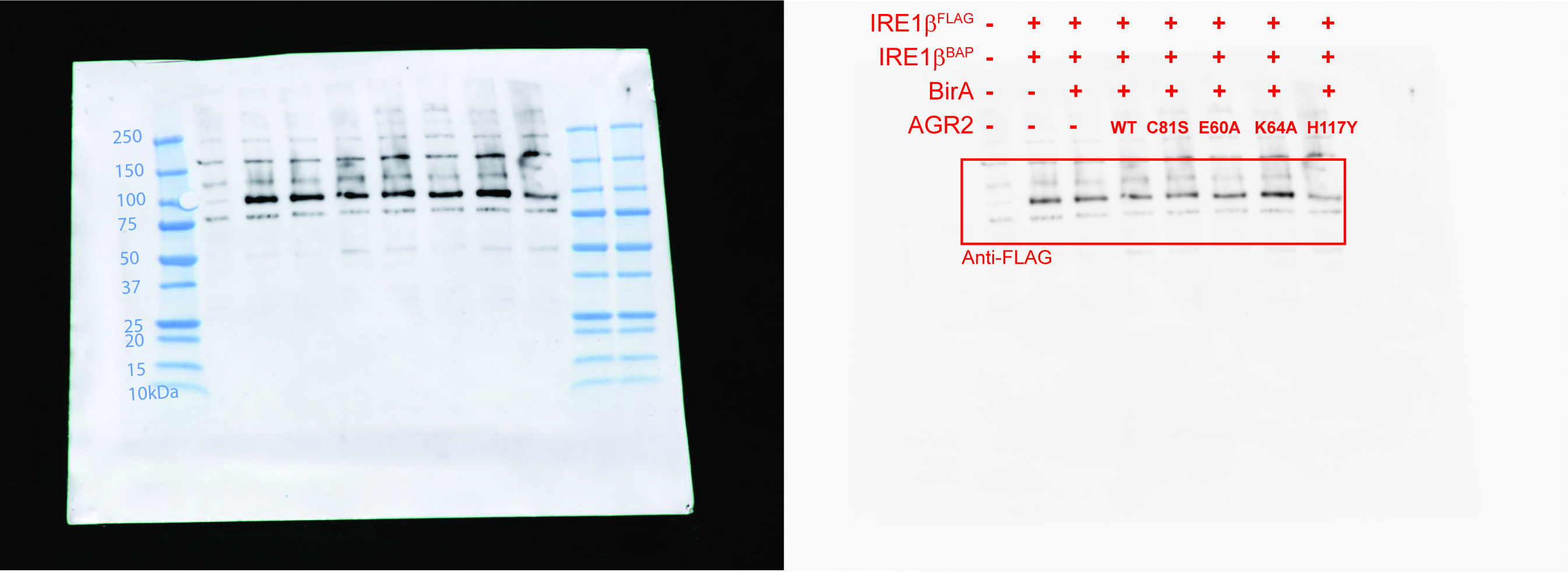

Supplement: Supplementary file 7 — Source Data Fig. 5 [file 44318_2023_15_MOESM7_ESM.zip › Figure 5/5C/western FLAG - input samples .tif]

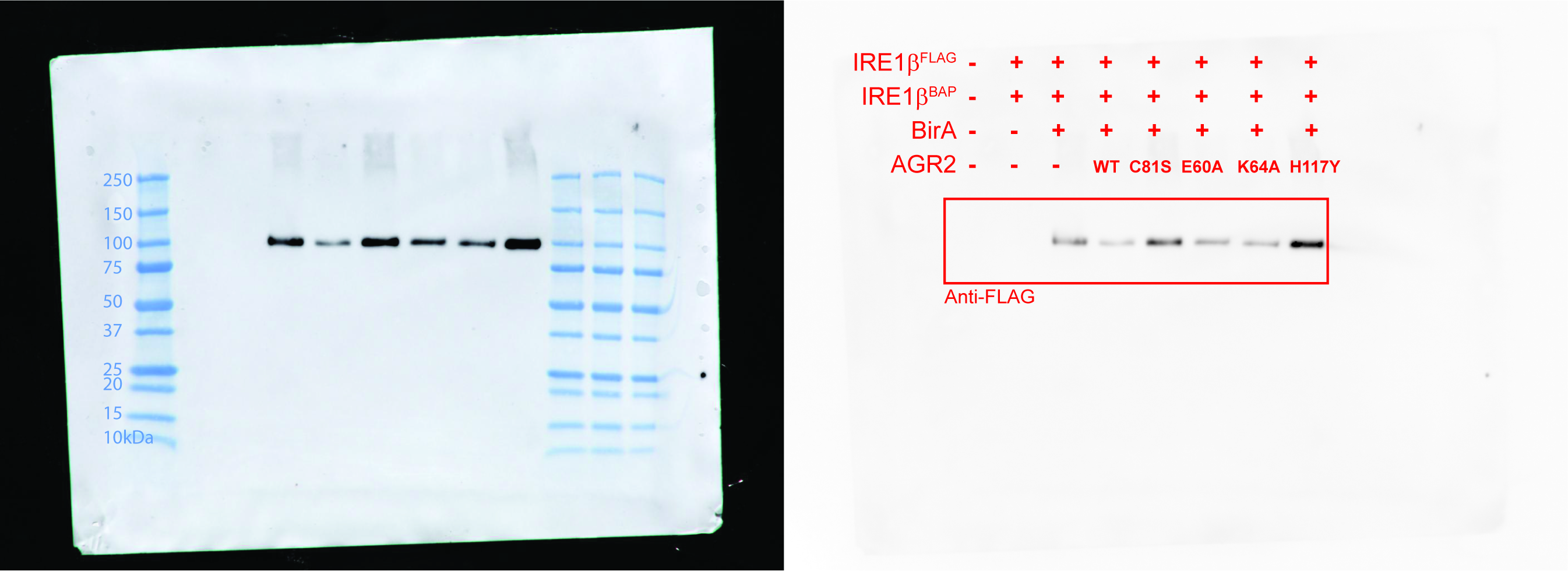

Supplement: Supplementary file 7 — Source Data Fig. 5 [file 44318_2023_15_MOESM7_ESM.zip › Figure 5/5C/western FLAG - IP samples .tif]

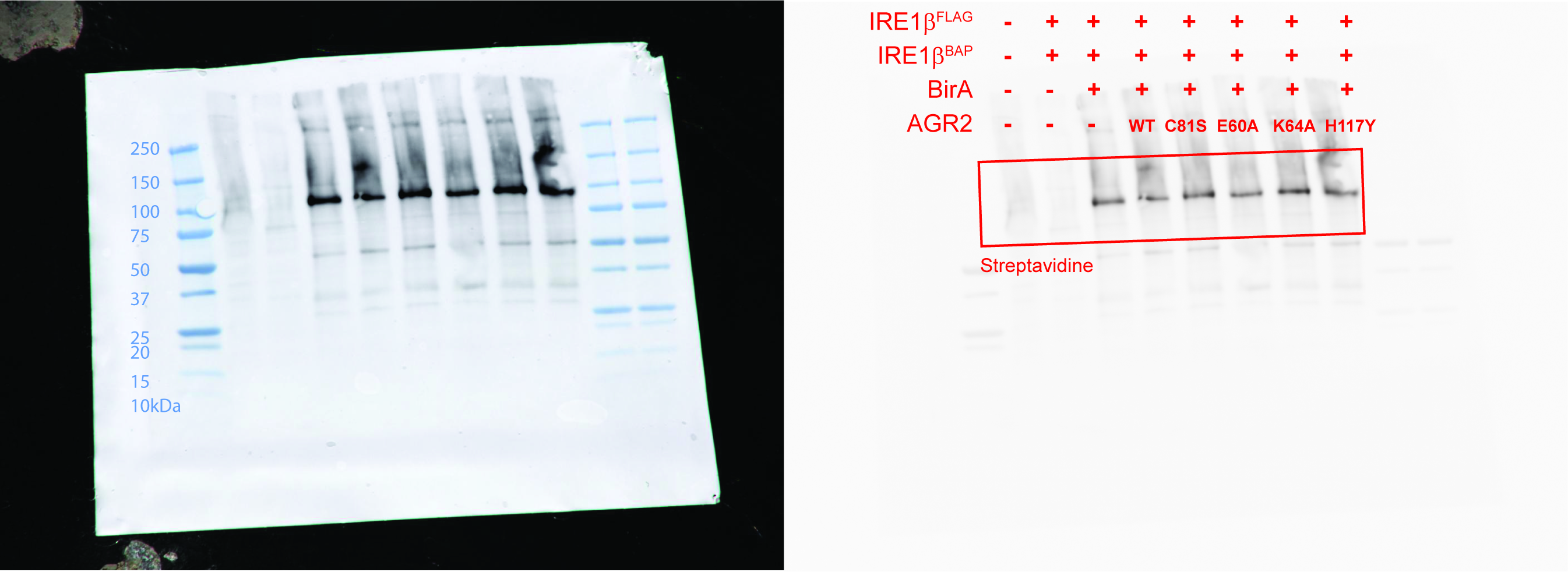

Supplement: Supplementary file 7 — Source Data Fig. 5 [file 44318_2023_15_MOESM7_ESM.zip › Figure 5/5C/western streptavidin - input samples .tif]

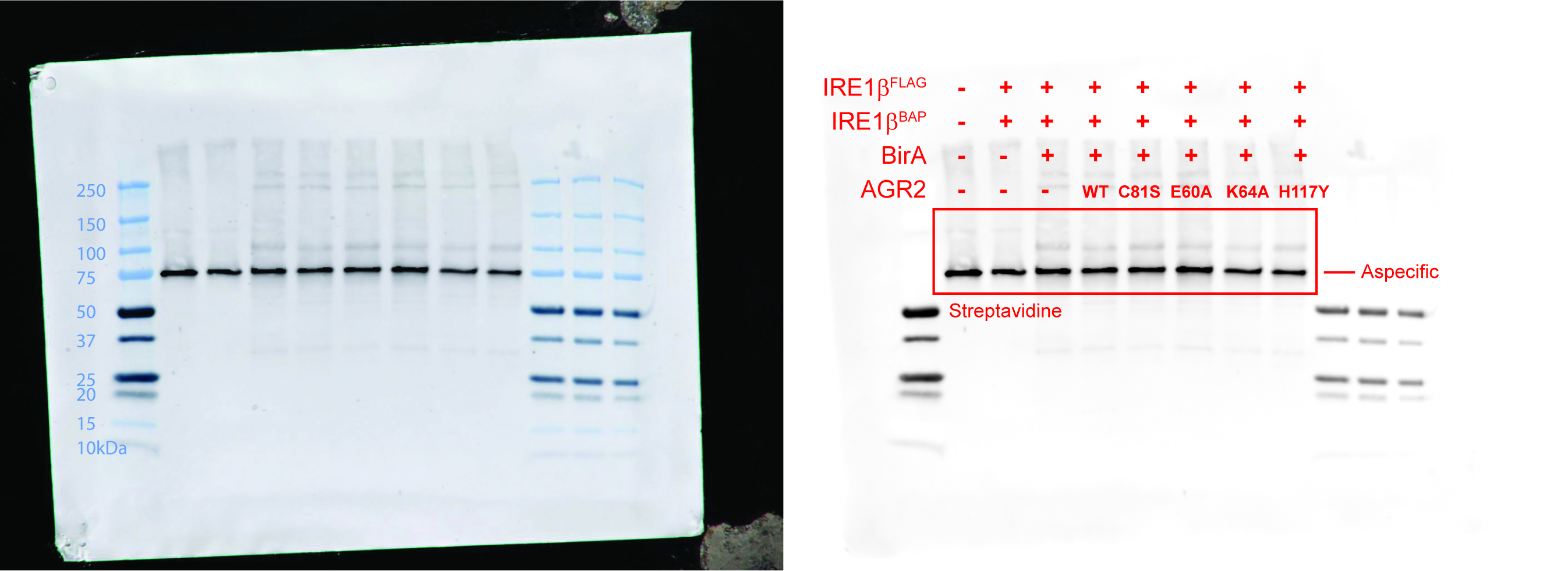

Supplement: Supplementary file 7 — Source Data Fig. 5 [file 44318_2023_15_MOESM7_ESM.zip › Figure 5/5C/western streptavidin - IP samples .tif]

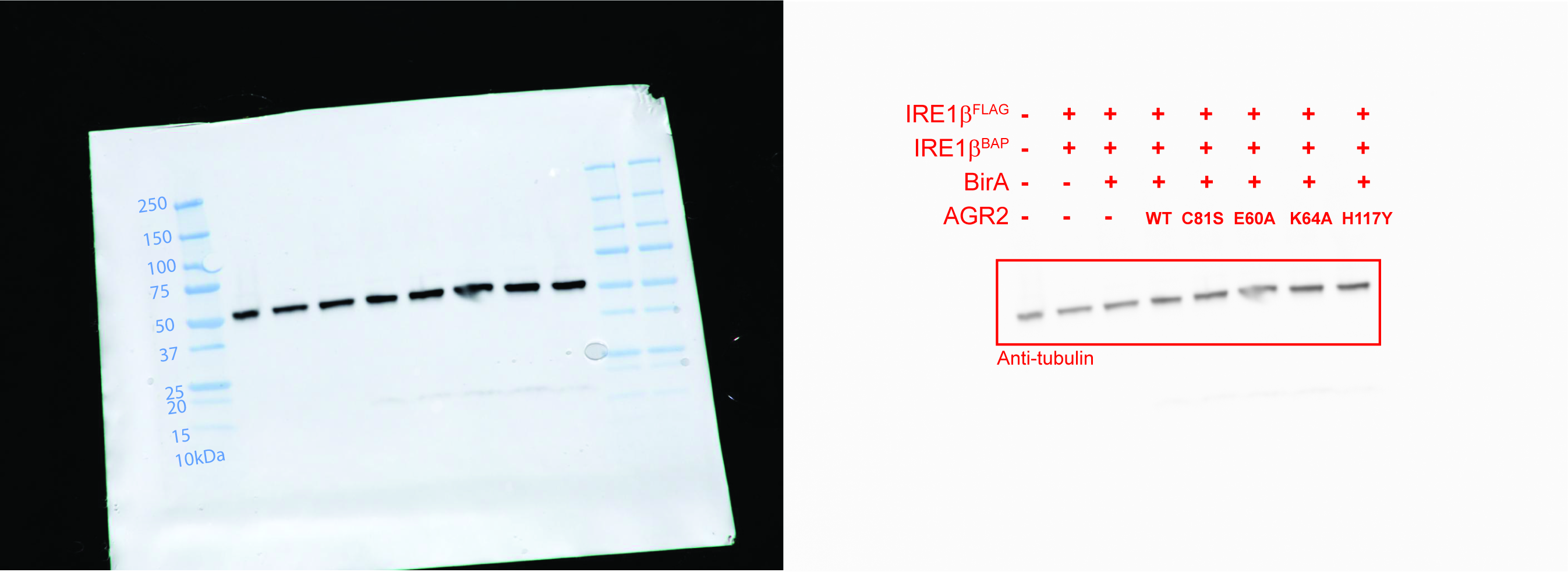

Supplement: Supplementary file 7 — Source Data Fig. 5 [file 44318_2023_15_MOESM7_ESM.zip › Figure 5/5C/western tubulin - input samples .tif]

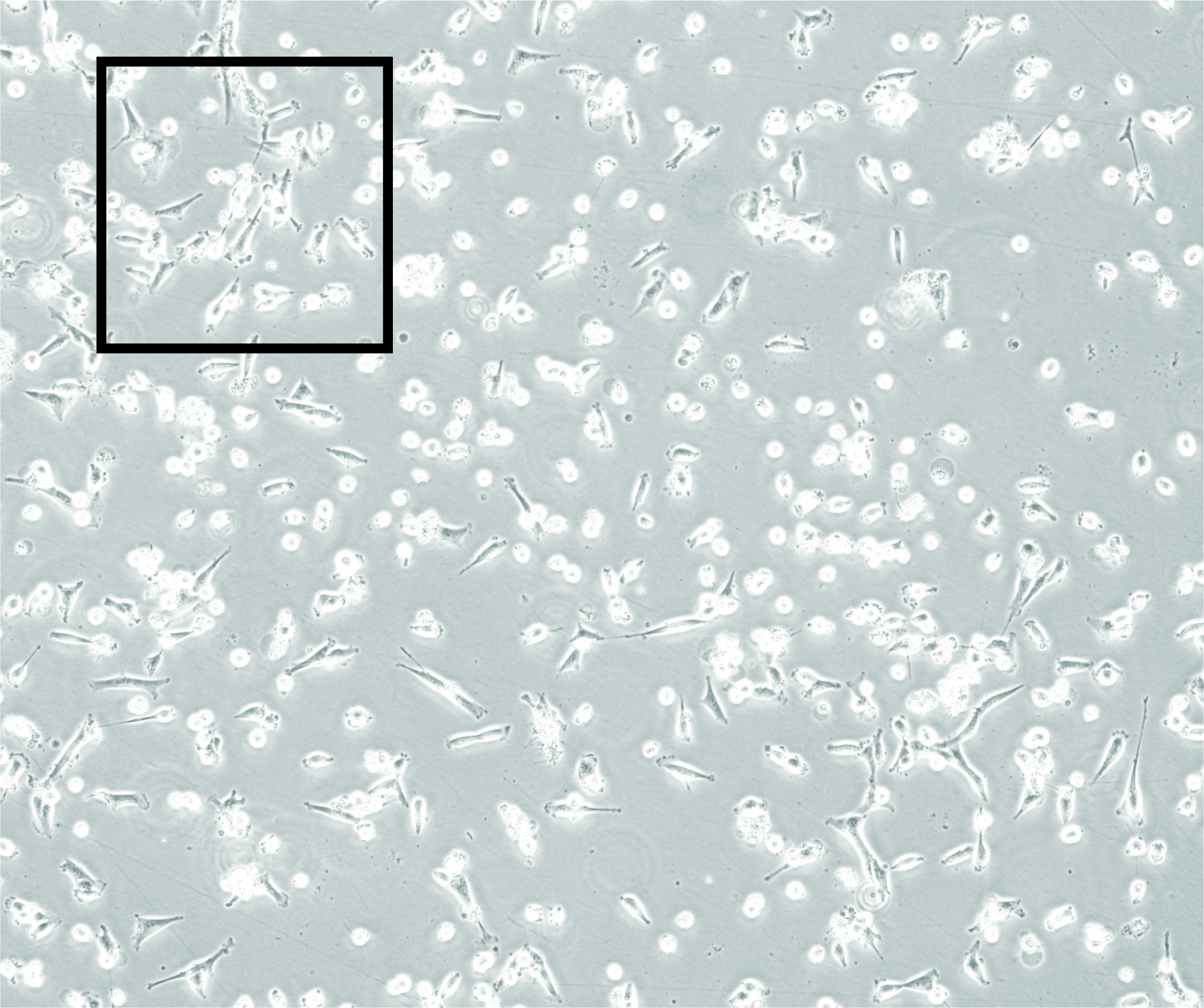

Supplement: Supplementary file 7 — Source Data Fig. 5 [file 44318_2023_15_MOESM7_ESM.zip › Figure 5/5E/C81 - doxycycline.tif]

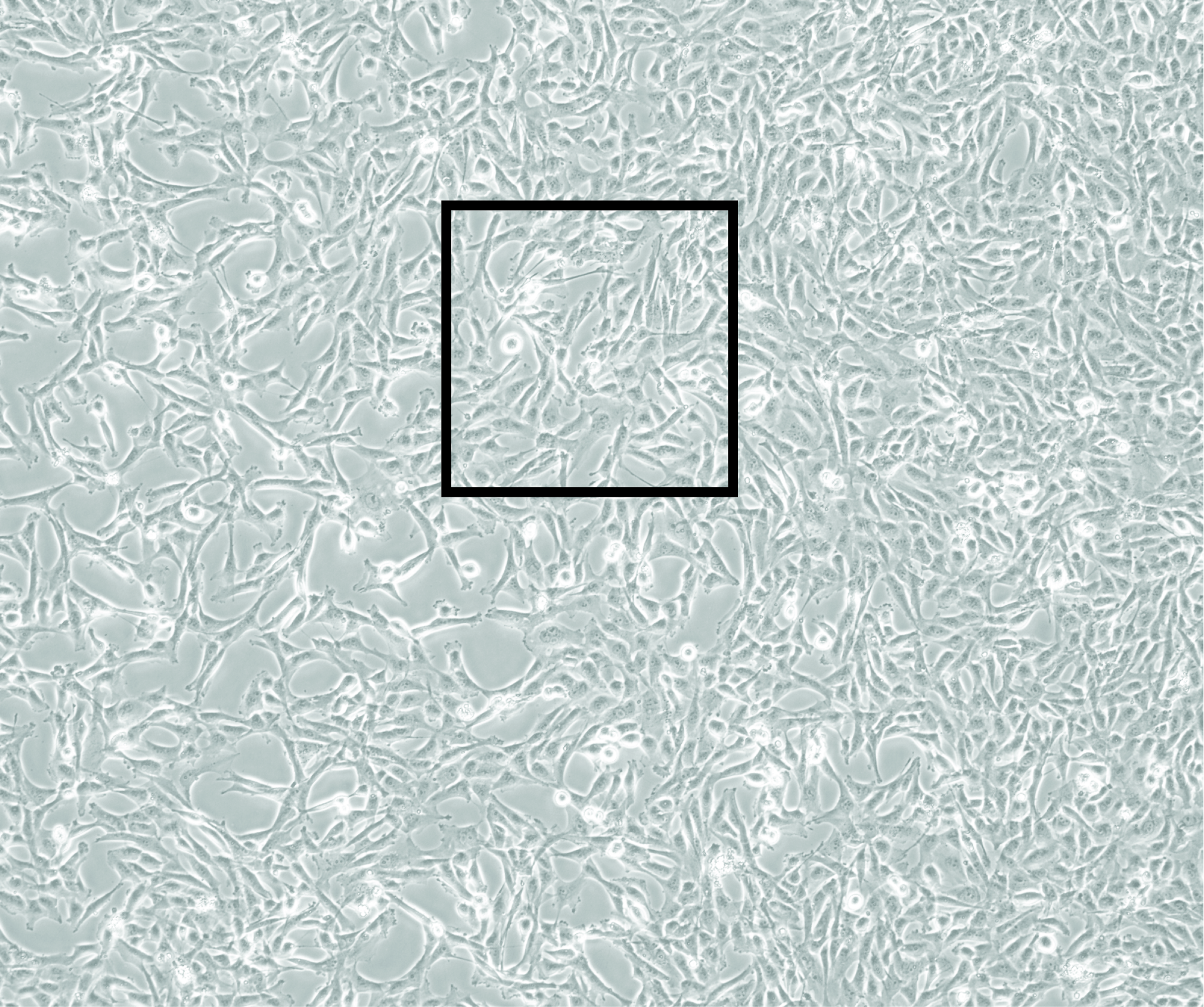

Supplement: Supplementary file 7 — Source Data Fig. 5 [file 44318_2023_15_MOESM7_ESM.zip › Figure 5/5E/C81 - no doxycycline.tif]

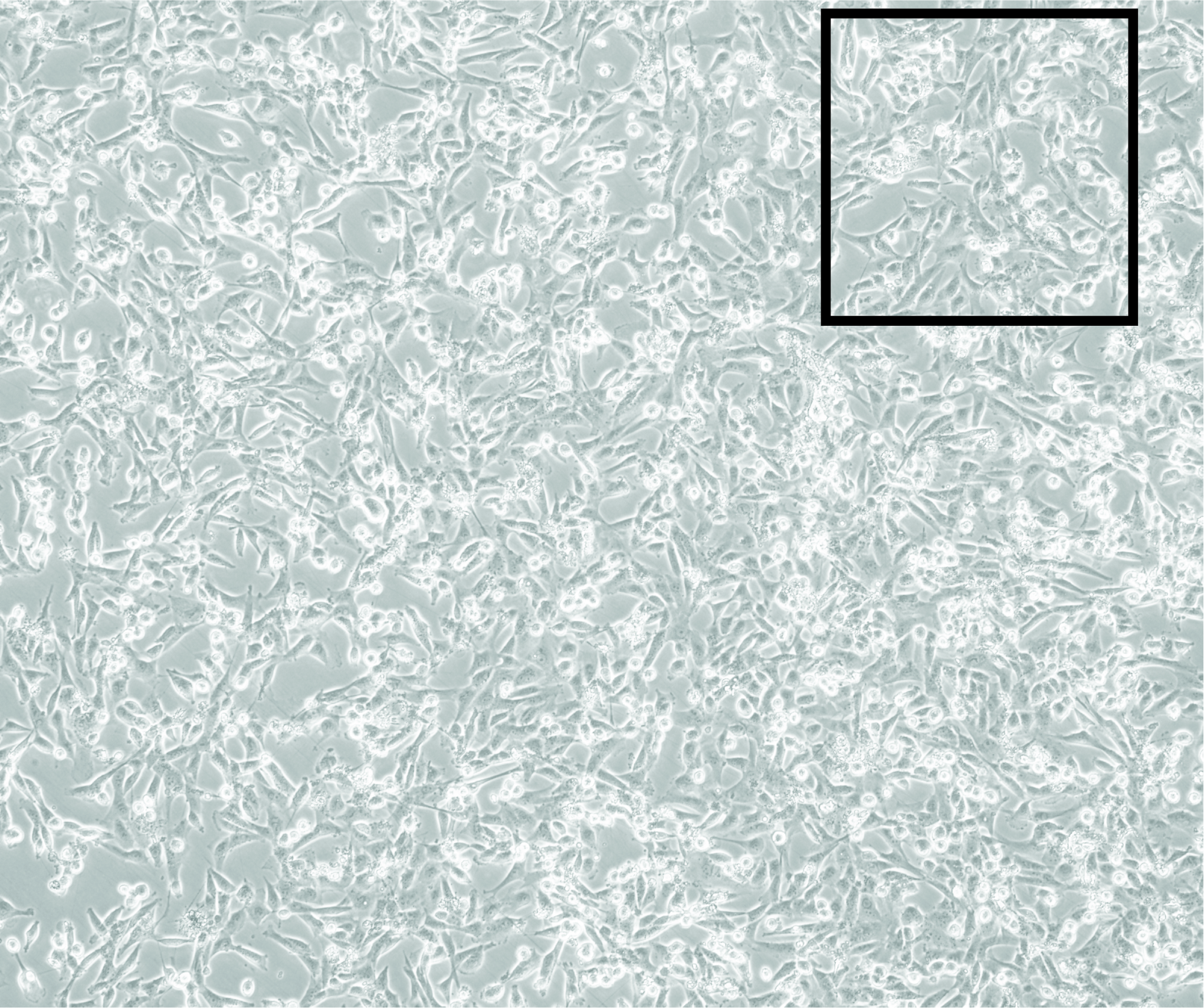

Supplement: Supplementary file 7 — Source Data Fig. 5 [file 44318_2023_15_MOESM7_ESM.zip › Figure 5/5E/E60 - doxycycline.tif]

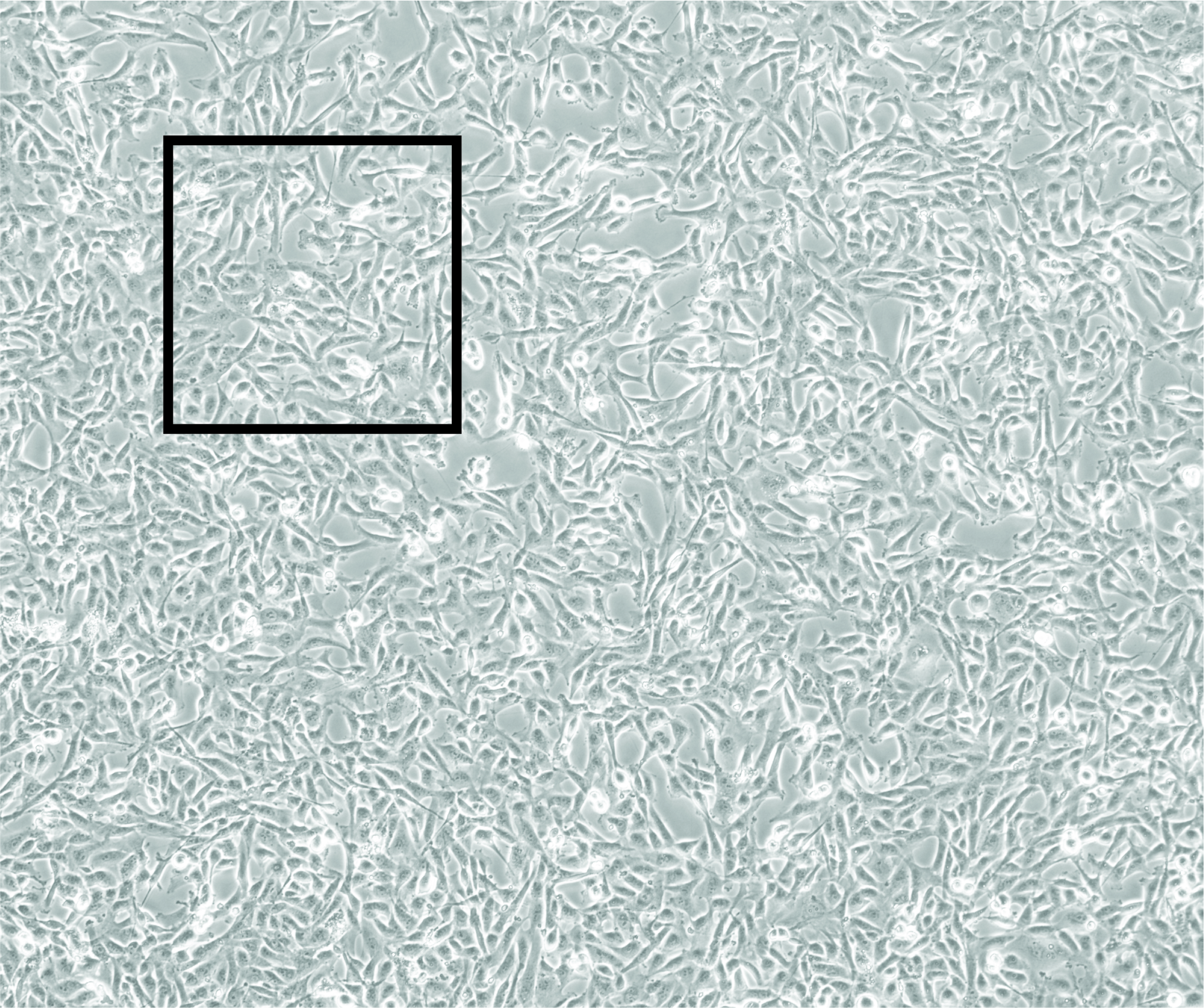

Supplement: Supplementary file 7 — Source Data Fig. 5 [file 44318_2023_15_MOESM7_ESM.zip › Figure 5/5E/E60 - no doxycycline.tif]

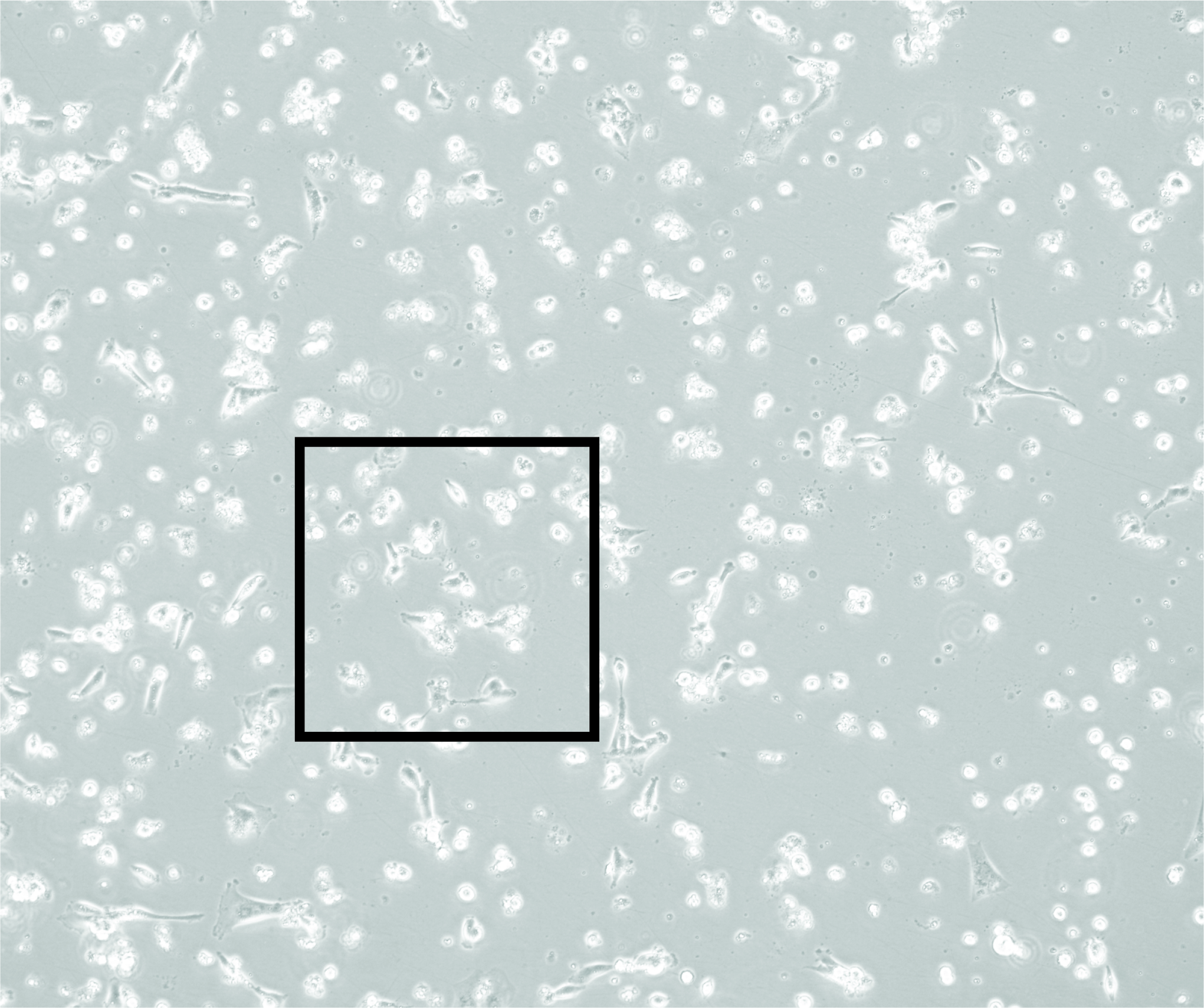

Supplement: Supplementary file 7 — Source Data Fig. 5 [file 44318_2023_15_MOESM7_ESM.zip › Figure 5/5E/H117Y - doxycycline.tif]

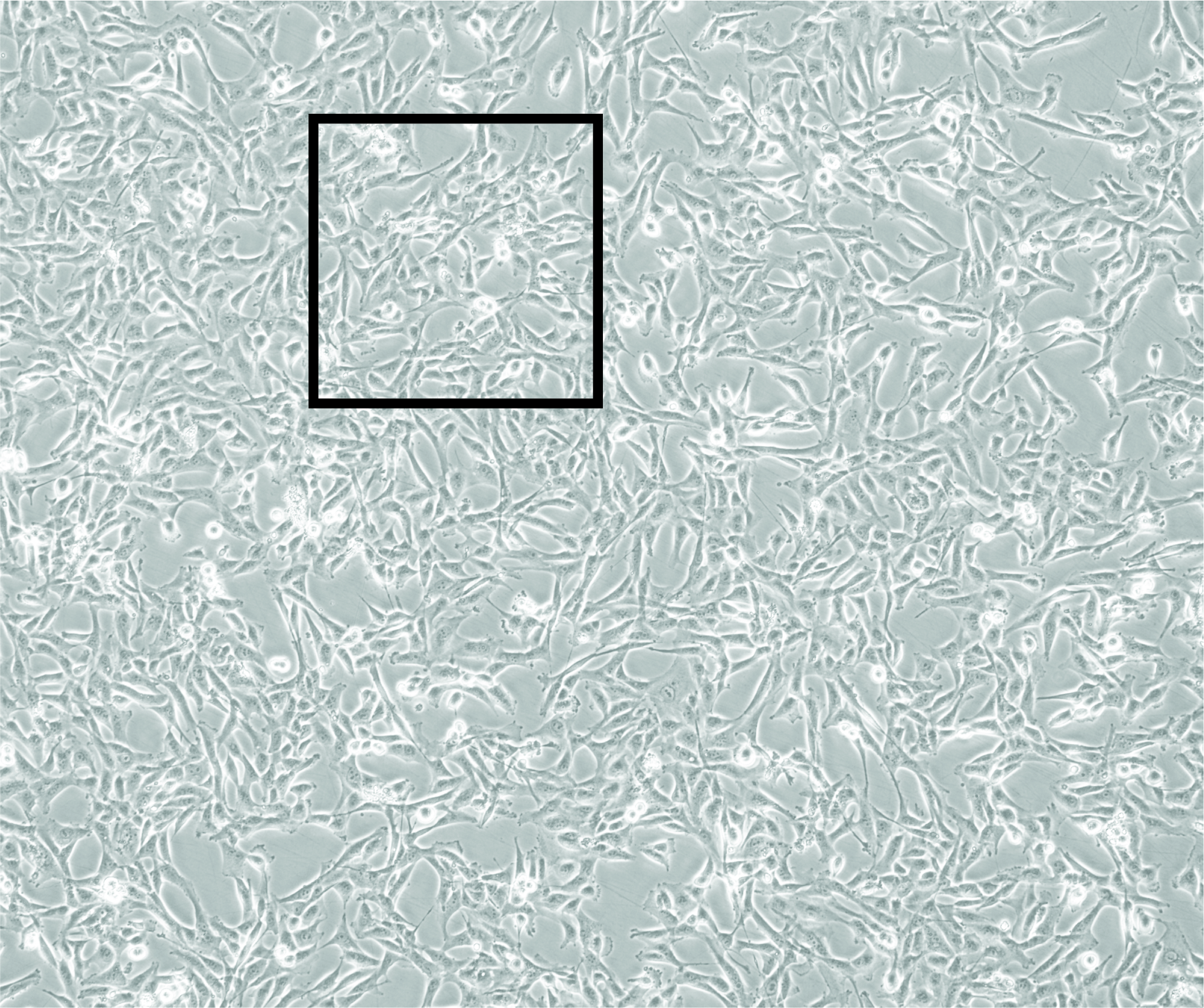

Supplement: Supplementary file 7 — Source Data Fig. 5 [file 44318_2023_15_MOESM7_ESM.zip › Figure 5/5E/H117Y - no doxycycline.tif]

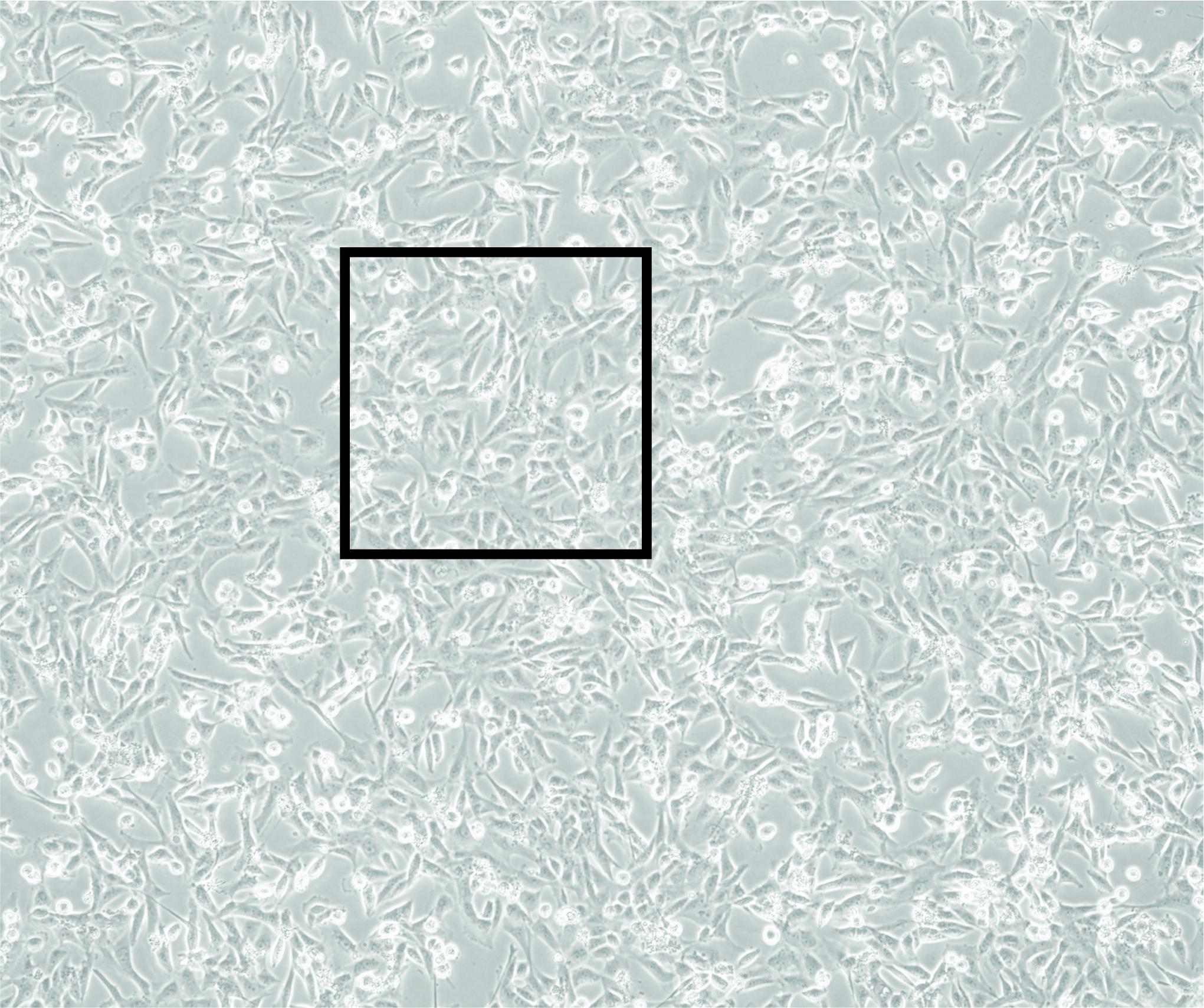

Supplement: Supplementary file 7 — Source Data Fig. 5 [file 44318_2023_15_MOESM7_ESM.zip › Figure 5/5E/K64 - doxycycline.tif]

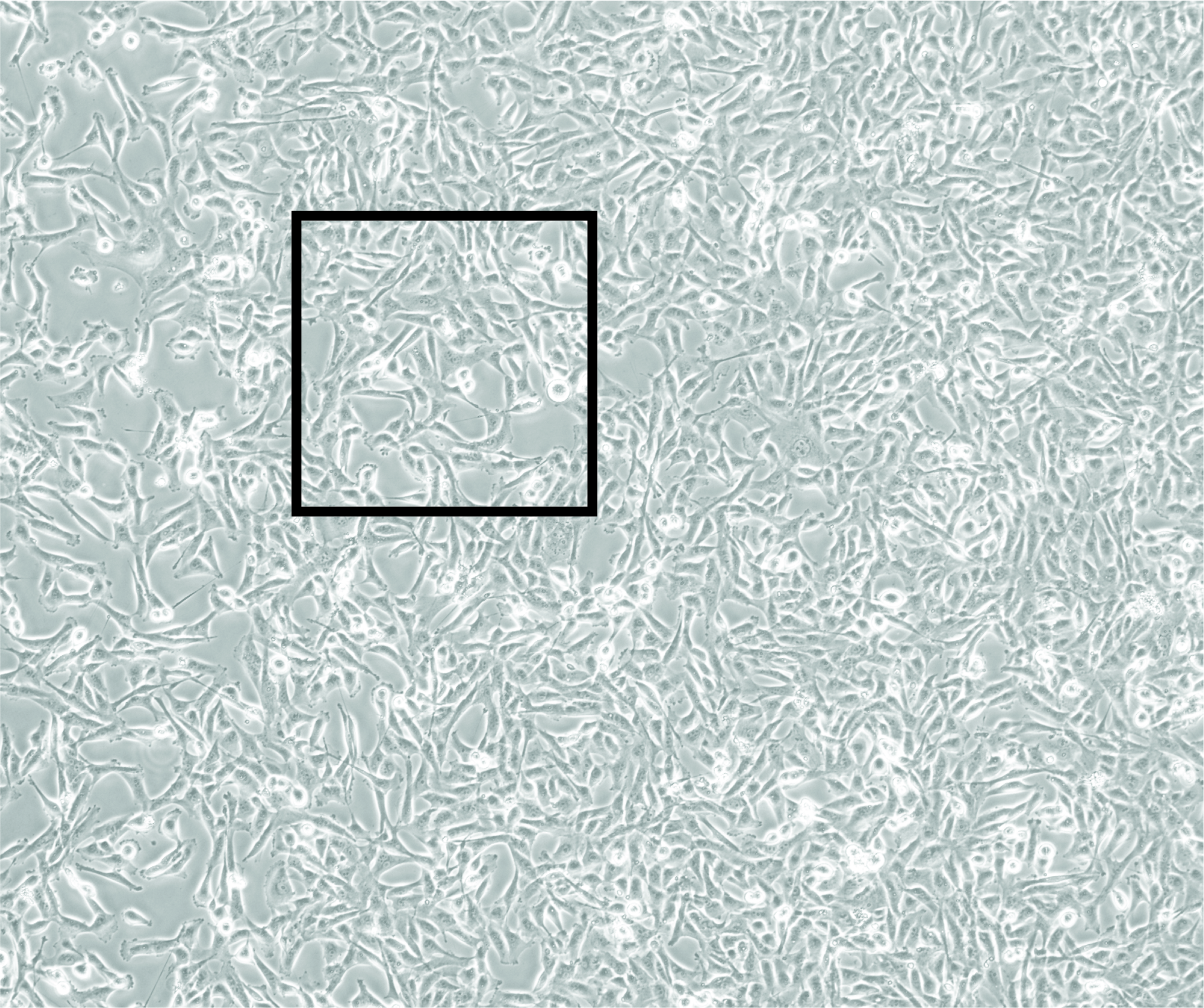

Supplement: Supplementary file 7 — Source Data Fig. 5 [file 44318_2023_15_MOESM7_ESM.zip › Figure 5/5E/K64 - no doxycycline.tif]

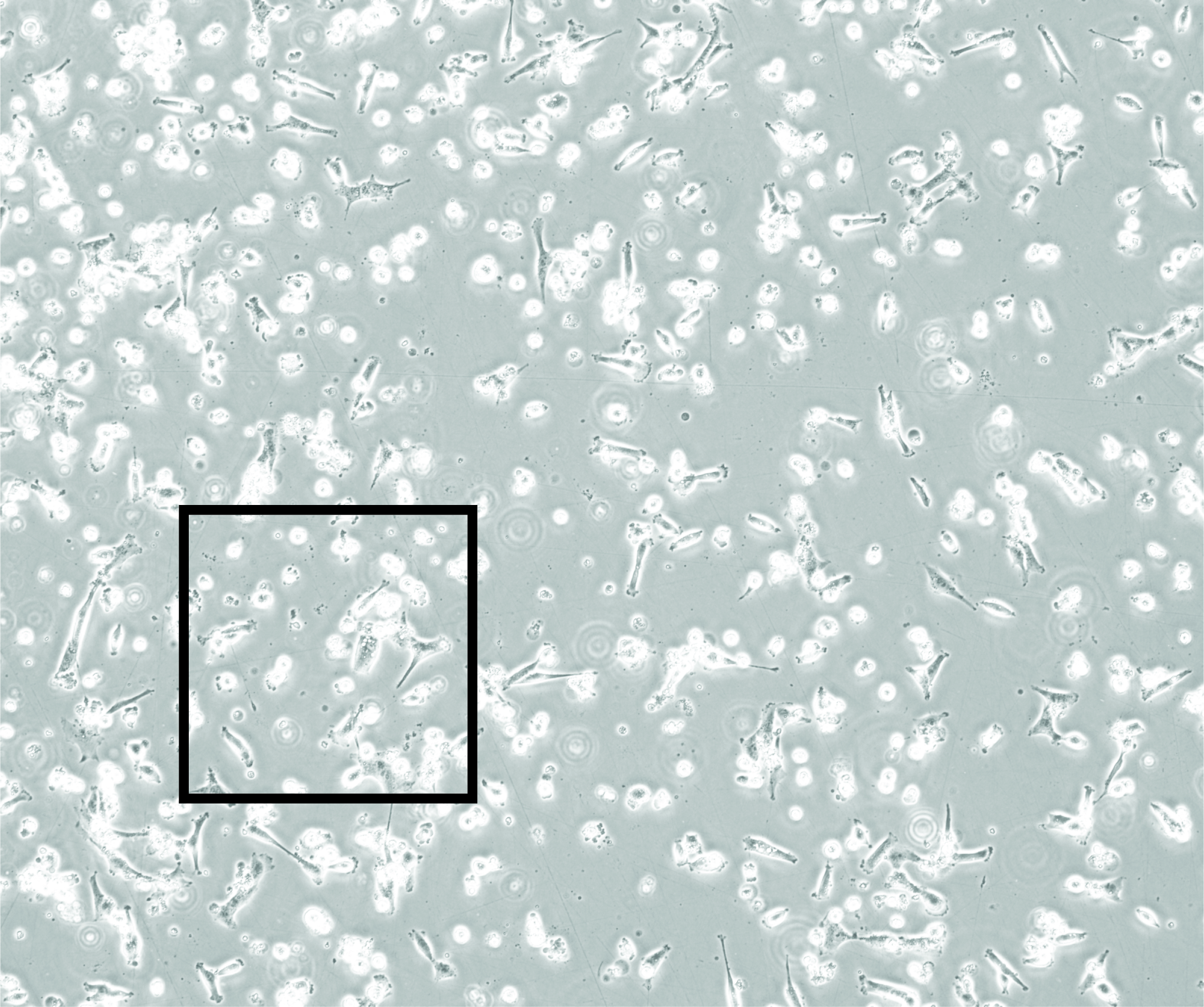

Supplement: Supplementary file 7 — Source Data Fig. 5 [file 44318_2023_15_MOESM7_ESM.zip › Figure 5/5E/No AGR2 - doxycycline.tif]

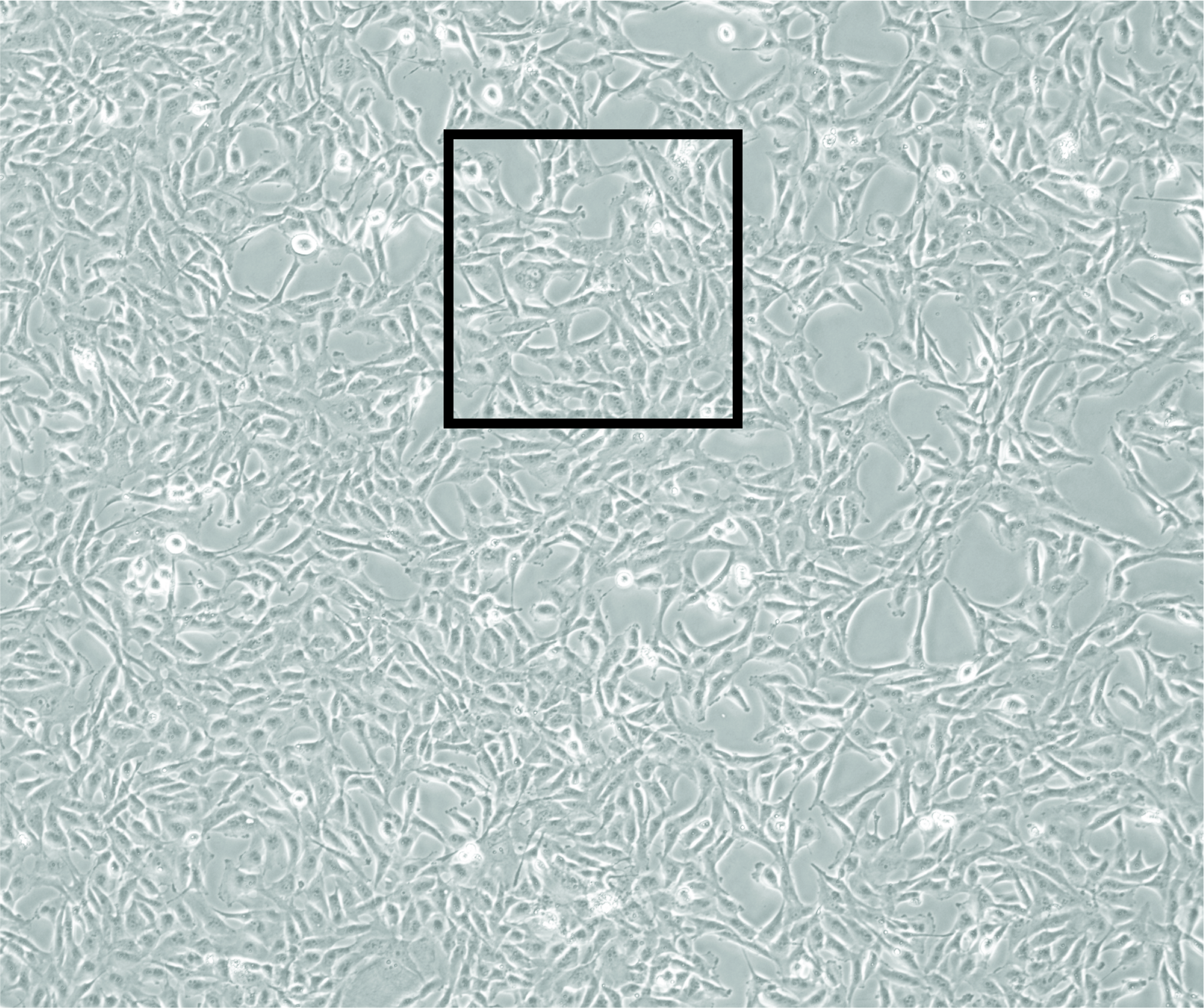

Supplement: Supplementary file 7 — Source Data Fig. 5 [file 44318_2023_15_MOESM7_ESM.zip › Figure 5/5E/No AGR2 - no doxycycline.tif]
