# Supplementary figures and images for: Activation of goblet-cell stress sensor IRE1β is controlled by the mucin chaperone AGR2 (part 2 of 2)
Source: EMBO J. 2023 Dec 20;43(5):3. doi: 10.1038/s44318-023-00015-y (PMC10907643; doi:10.1038/s44318-023-00015-y)

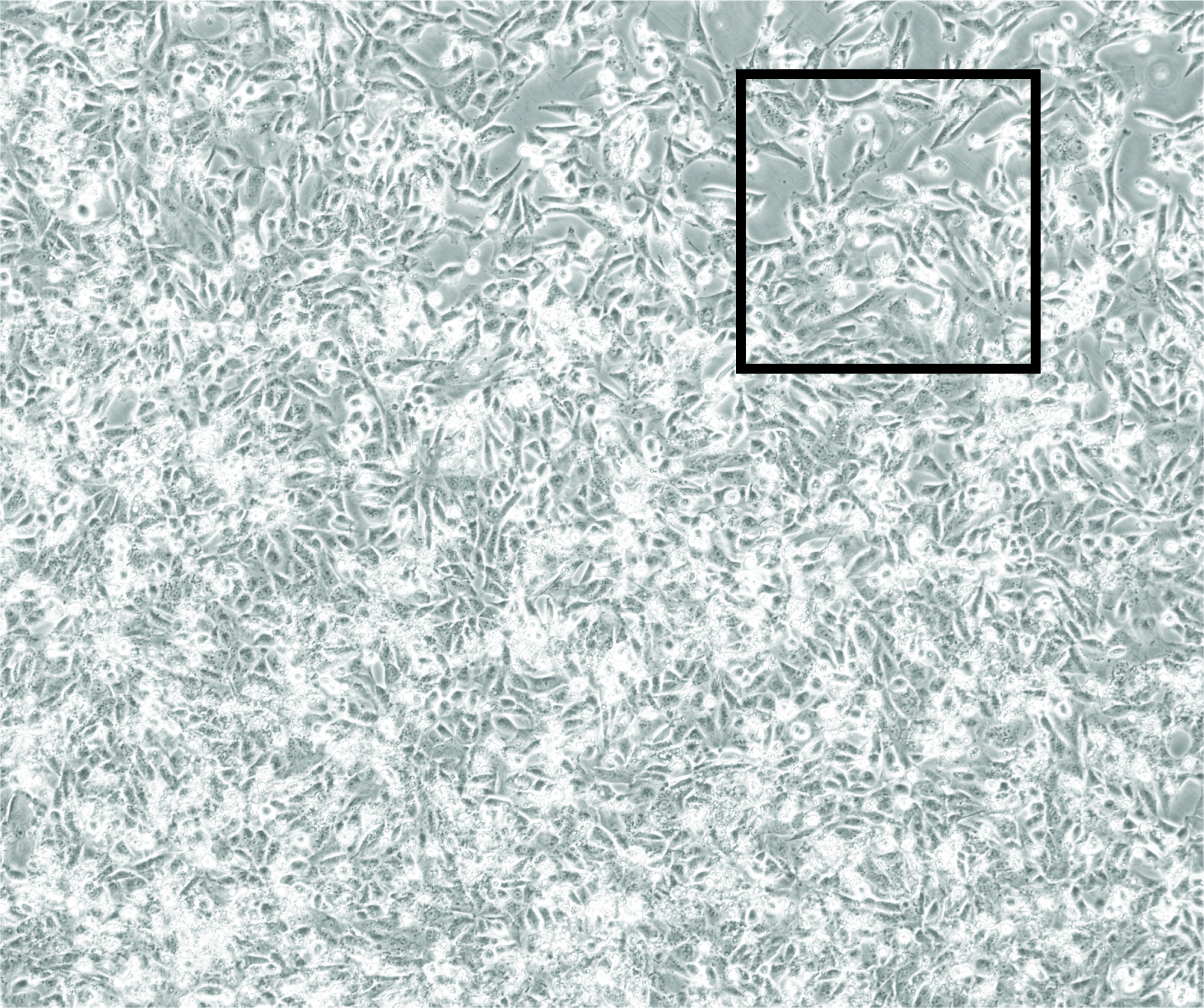

Supplement: Supplementary file 7 — Source Data Fig. 5 [file 44318_2023_15_MOESM7_ESM.zip › Figure 5/5E/WT - doxycycline.tif]

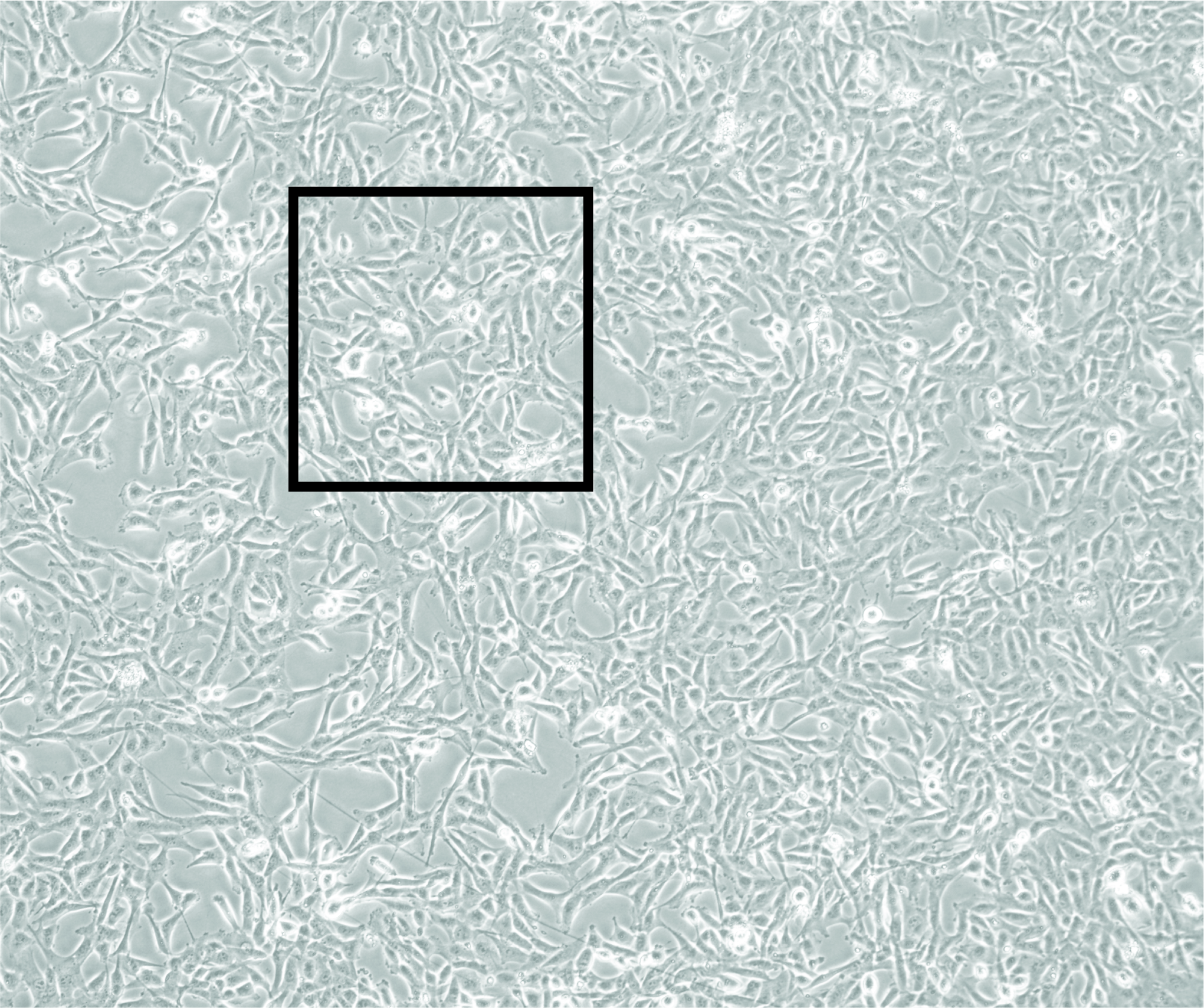

Supplement: Supplementary file 7 — Source Data Fig. 5 [file 44318_2023_15_MOESM7_ESM.zip › Figure 5/5E/WT - no doxycycline.tif]
