# Supplementary material for: Activation of goblet-cell stress sensor IRE1β is controlled by the mucin chaperone AGR2
Source: EMBO J. 2023 Dec 20;43(5):3. doi: 10.1038/s44318-023-00015-y (PMC10907643; doi:10.1038/s44318-023-00015-y)
Supplement: Supplementary file 8 — Expanded View Figures [file 44318_2023_15_MOESM8_ESM.pdf]

Expanded View Figures

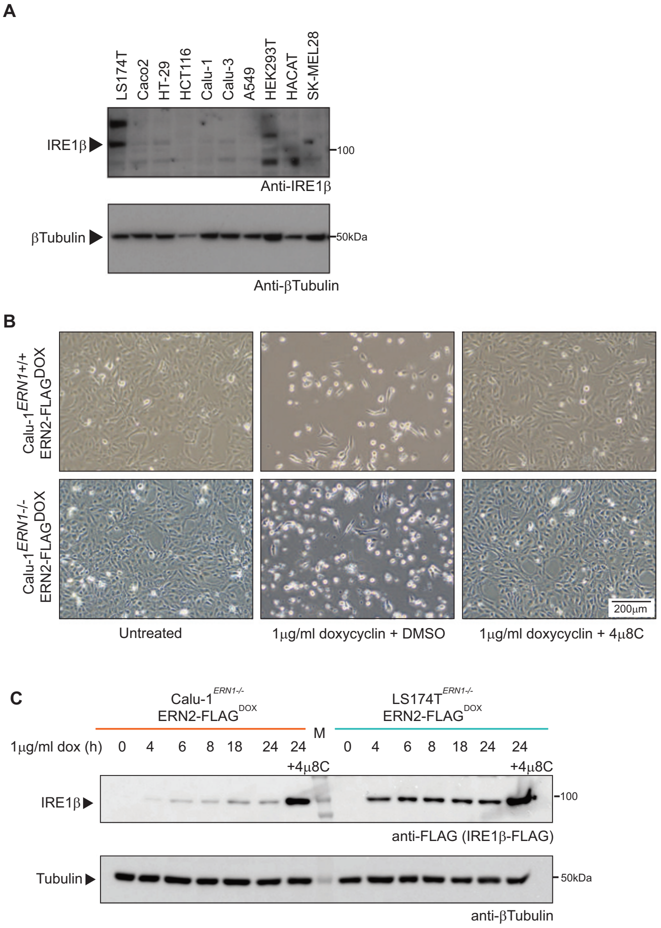

Figure EV1. Validation of LS174T$^{ERN1-/-IRE1βFLAG-DOX}$ and Calu-1 $^{ERN1-/-IRE1βFLAG-DOX}$ model systems.

(**A**) IRE1β expression in cell lines. Proteins were extracted and probed for IRE1β expression via immunoblot. Tubulin was used as a loading control. (**B**) Photographs showing the phenotype of cultures overexpressing IRE1β-FLAG in IRE1α wild-type (*ERN1*$^{+/+}$) and IRE1α deficient (*ERN1*$^{-/-}$) cells. Images of IRE1α deficient (*ERN1*$^{-/}$_) cultures are the same images as shown in Fig. 1C. Left panels show untreated cultures, middle panels show cultures treated with 1 μg/ml doxycycline for 72 hours, right panels show cultures treated with both 1 μg/ml doxycycline and 1 μM IRE1 endonuclease inhibitor 4μ8C. Scale bar represents 200 μm. (**C**) Quantification of IRE1β transgene expression over time in Calu-1$^{ERN1-/-IRE1βFLAG-DOX}$ and LS174T$^{ERN1-/-IRE1βFLAG-DOX}$ cells by western blot. Cultures were treated with 1 μg/ml doxycycline for the indicated times, protein lysates were probed for IRE1β-FLAG expression and tubulin as a loading control.

© The Author(s)

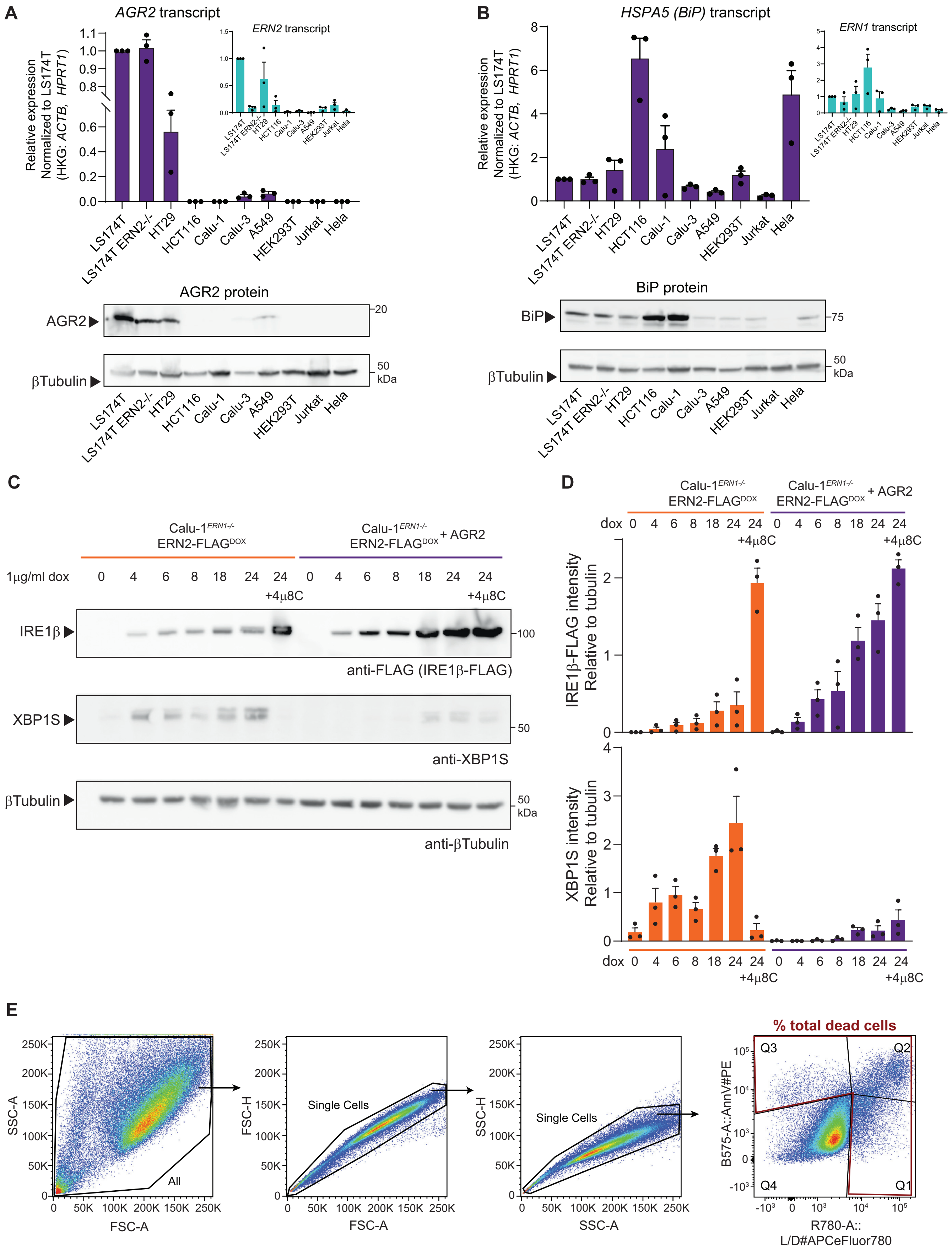
A
AGR2 transcript
Relative expression Normalized to LS174T (HKG: ACTB, HPRT1)
ERN2 transcript
LS174T
LS174T ERN2-/-
HT29
HCT116
Calu-1
Calu-3
A549
HEK293T
Jurkat
Hela
AGR2 protein
AGR2
βTubulin
20
50 kDa
B
HSPA5 (BiP) transcript
ERN1 transcript
BiP protein
BiP
75
C
Calu-1ERN1-/- ERN2-FLAGDOX
Calu-1ERN1-/- ERN2-FLAGDOX + AGR2
1μg/ml dox
+4μ8C
IRE1β
anti-FLAG (IRE1β-FLAG)
100
XBP1S
anti-XBP1S
anti-βTubulin
D
dox
IRE1β-FLAG intensity Relative to tubulin
XBP1S intensity Relative to tubulin
E
% total dead cells
SSC-A
FSC-A
FSC-H
SSC-H
All
Single Cells
B575-A::AnnV#PE
R780-A:: L/D#APCeFluor780
Q1
Q2
Q3
Q4

 © The Author(s)

Figure EV2. AGR2 expression is restricted to colon epithelial cell lines and affects Calu-1$^{ERN1-/-IRE1\beta FLAG-DOX}$ phenotype.

(**A**) *AGR2* (purple) and *ERN2* (blue insert, same data as shown in Fig. 1A) transcript expression in cell lines assayed by RT-qPCR. $N = 3$ culture dishes were sampled and *AGR2* and *ERN2* expression is shown relative to the expression detected in LS174T parental cells. Error bars show SEM. Bottom picture shows protein expression by western blot. Protein lysates were probed for AGR2 and tubulin was used as a loading control. (**B**) *HSPA5* (purple) and *ERN1* (blue insert, same data as shown in Fig. 1A) transcript expression in cell lines assayed by RT-qPCR. $N = 3$ culture dishes were sampled and *HSPA5* and *ERN2* expression is shown relative to the expression detected in LS174T parental cells. Error bars show SEM. Bottom picture shows protein expression by western blot. Protein lysates were probed for BiP and tubulin was used as a loading control. (**C**) IRE1β-FLAG transgene expression over time by western blot in cell lysates derived from Calu-1$^{ERN1-/-IRE1\beta FLAG-DOX}$ co-expressing ER-targeted BirA as a control protein (left, orange), or AGR2 (right, purple). Cells received 1 µg/ml doxycycline to induce expression of IRE1β. Protein lysates were prepared at the indicated times and probed for IRE1β-FLAG expression, XBP1S and tubulin as a loading control. (**D**) Quantification of IRE1β-FLAG and XPP1S protein levels normalized to tubulin in three replicate experiments represented in (**C**). Error bars represent SEM. (**E**) Gating strategy to assess cell death. Doublets were gated out and dead cells were gated on via Annexin V and Live/Dead positive staining. All cells staining positive for a single, or both cell death markers were considered dead (red gate).

© The Author(s)

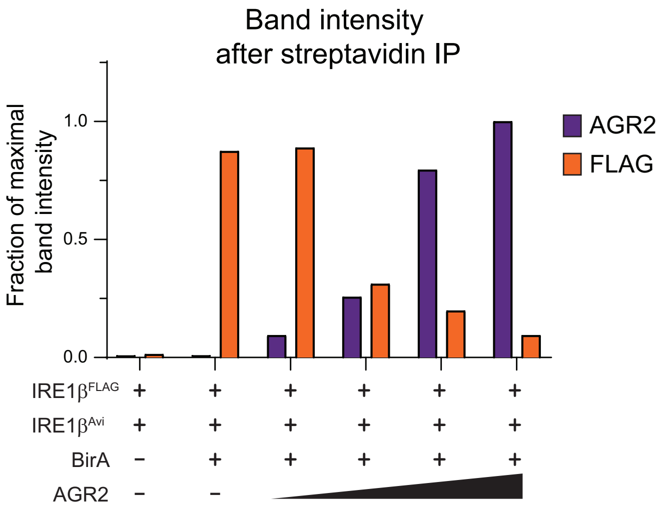

Figure EV3. Inverse relationship between AGR2 IP and IRE1β complex formation.

Quantification of immunoblots in Fig. 4F ($n = 2$). IRE1β-FLAG band intensity was normalized to input expression levels. Band intensity values are expressed as a fraction of the maximal intensity that was obtained in each respective experiment.

© The Author(s)

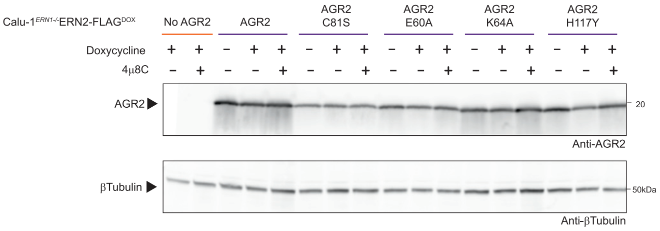

Figure EV4. AGR2 expression in stably transduced cell lines.

AGR2 expression in cultures analyzed in Fig. 5D,E. Tubulin was used as a loading control.

© The Author(s)

A

```
ERN1_human   89 NNEGLTKLPFTIPELVQASPCRSSDGILYMGKKQDIWYVIDLLTGEKQQTLSSAFADSLC 148
ERN2_human   97 KQQGLMKLPFTIPELVHASPCRSSDGVFYTGRKQDAWFVVDPESGETQMTLTTE-----G 151

ERN1_human  149 PSTSLLYLGRTEYTITMYDTKTRELRWNATYFDYAASLPEDDVDYKMSHFVSNGDGLVVT 208
ERN2_human  152 PSTPRLYIGRTQYTVTMHDPRAPALRWNTTYRRYSAPPMDGSPGKYMSHLASCGMGLLLT 211

                ...

ERN1_human  324 VTIGDKGECVITPSTDVKFDPGLKSKNKLNYLRNYWLLIGHHETPLSASTKMLERFPNNL 383
ERN2_human  328 VTLQVSGEREGSPSTAVRYPSGSVA------LPSQWLLIGHHELPPVLHTTMLRVHPTLG 381
```

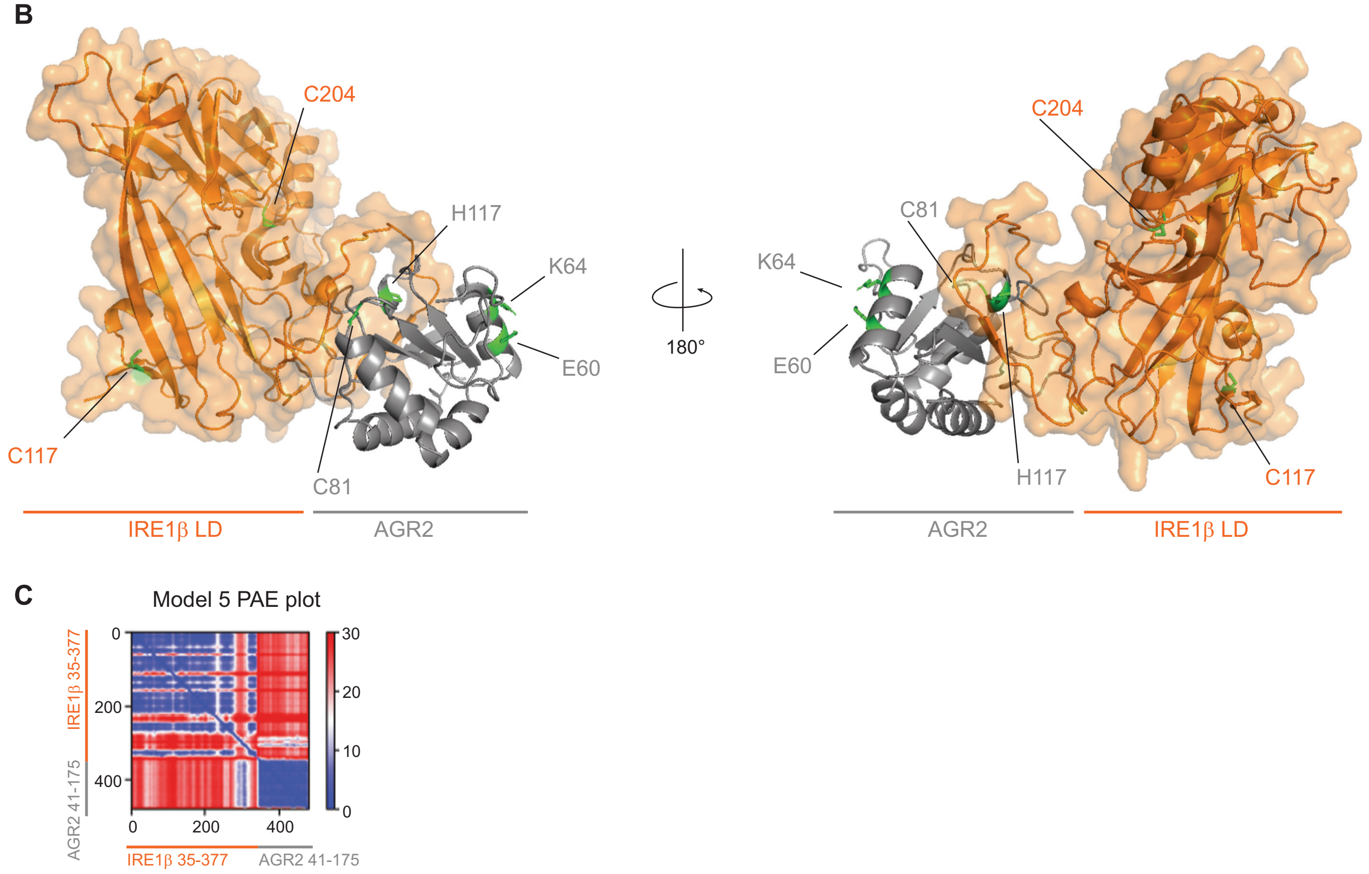

Figure EV5. AGR2 is predicted to bind a flexible loop region in the IRE1β luminal domain.

(**A**) BLAST alignment of the regions containing cysteines in human IRE1α and IRE1β. Green square indicates the sole conserved cysteine in IRE1α and IRE1β luminal domain, orange squares show cysteines present in only one of the paralogues. (**B**) Highest scoring AlphaFold2-Multimer model (pTM score = 0.662), modeled using IRE1β residues 35-377 (Uniprot Q76MJ5) and AGR2 residues 41-175 (Uniprot O95994). The IRE1β luminal domain is shown in orange and AGR2 in grey. Labels indicate the highlighted green residues. (**C**) Predicted aligned error (PAE) plot for the model shown in B.

 © The Author(s)
